# Nasal Delivery of Engineered Exosomes via a Thermo‐Sensitive Hydrogel Depot Reprograms Glial Cells for Spinal Cord Repair

**DOI:** 10.1002/advs.202504486

**Published:** 2025-06-20

**Authors:** Yangyang Wang, Jingsong Liu, Pengfei Li, Zhibin Peng, Yubo Zhang, Yishu Liu, Mi Li, Xuqiang Gong, Daqian Liu, Enze Xu, Hongbo Yang, Yuanliang Sun, Yan Xu, Yansong Wang

**Affiliations:** ^1^ Department of Orthopedic Surgery, The First Affiliated Hospital of Harbin Medical University Harbin Medical University Harbin 150000 P. R. China; ^2^ The Key Laboratory of Myocardial Ischemia, Ministry of Education Harbin Medical University Harbin 150000 P. R. China; ^3^ NHC Key Laboratory of Cell Transplantation Harbin Medical University Harbin 150000 P. R. China; ^4^ Center for Endemic Disease Control Chinese Center for Disease Control and Prevention Harbin Medical University Harbin 150000 P. R. China; ^5^ Key Lab of Etiology and Epidemiology, Education Bureau of Heilongjiang Province & Ministry of Health (23618504) Harbin Medical University Harbin Heilongjiang Province 150000 P. R. China; ^6^ Research Center for Human Tissue and Organs Degeneration Institute of Biomedicine and Biotechnology Shenzhen Institutes of Advanced Technology Chinese Academy of Sciences Shenzhen 518055 P. R. China; ^7^ Department of Orthopedic Surgery, Affiliated Hospital of Chifeng Medical University Harbin Medical University Chifeng 0240005 P. R. China

**Keywords:** astrocyte‐neuron crosstalk, m2c macrophages, reprogrammed exosomes, spinal cord injury, thermosensitive chitosan hydrogel

## Abstract

Spinal cord injury (SCI) presents formidable therapeutic challenges due to its multifaceted pathological complexity. Here, this work reports engineered macrophage‐derived exosomes overexpressing GNA12 and GNA13 (G12G13MExos) that reprogram macrophages toward the M2c anti‐inflammatory phenotype and astrocytes into a neuroprotective phenotype. G12G13MExos enhance astrocyte‐mediated clearance of myelin debris, glutamate homeostasis, and synapse formation while fostering astrocyte‐neuron crosstalk. These effects improve neuronal survival and drove neural stem cell differentiation into V2a neurons, facilitating neural circuit reconstruction. This work develops a chitosan‐based thermosensitive hydrogel that functions as a “nasal exosome intelligent slow‐release depot” to enable efficient and targeted exosome delivery. This delivery system bypasses hepatic and renal sequestration and overcomes the blood‐spinal cord barrier, significantly enhancing therapeutic efficacy. This strategy integrates engineered exosomes with a responsive delivery platform, modulating the inflammatory microenvironment, enhancing cellular crosstalk, and promoting neural repair. This comprehensive approach offers a promising translational avenue for SCI treatment and other central nervous system disorders.

## Graphical abstract

Engineered macrophage‐derived exosomes overexpressing GNA12 and GNA13 reprogram glial cells to promote neuroprotection and neural regeneration following SCI. A chitosan‐based thermosensitive hydrogel enables sustained intranasal exosome delivery, bypassing systemic barriers and enhancing therapeutic efficacy. This strategy modulates the inflammatory microenvironment, promotes astrocyte‐neuron crosstalk, and facilitates neural circuit reconstruction, offering a promising translational approach for SCI treatment.

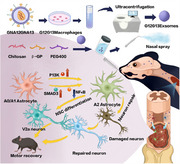

## 1 Introduction

### 1.1 Promoting SCI Repair by Modulating the Glial Microenvironment

Spinal cord injury (SCI) is a severe central nervous system disorder characterized by secondary damage, which is exacerbated by inflammation, oxidative stress, and disruption of the blood‐spinal cord barrier (BSCB)^[^ ^1^ ^]^ Among these factors, the inflammatory response serves as a key driver of secondary injury, orchestrated by the complex interactions between microglia, astrocytes, and infiltrating immune cells.^[^ ^2^ ^]^ Modulating this inflammatory microenvironment is critical for reducing neuronal apoptosis and promoting tissue repair.

Microglia are the earliest responders following SCI, and their phenotypic polarization significantly influences the repair process. The pro‐inflammatory M1 phenotype exacerbates injury by secreting inflammatory cytokines and recruiting infiltrating macrophages, thereby delaying recovery.^[^ ^3^ ^]^ Astrocytes, the most abundant glial cells in the central nervous system, play crucial roles in maintaining metabolic homeostasis and supporting neuronal function.^[^ ^4^ ^]^ After SCI, reactive astrocytes can either aggravate neural damage or facilitate repair, depending on their phenotype.^[^ ^5^ ^]^ Emerging evidence indicates that crosstalk between astrocytes, neurons, and neural stem cells is vital for functional recovery.^[^ ^6^ ^]^ Therefore, promoting the beneficial phenotypic transformation of microglia and astrocytes optimizes the early injury microenvironment and establishes favorable conditions for neural regeneration in later stages.

### 1.2 M2c Macrophage‐Derived Exosomes: A Novel Strategy for Glial Regulation

M2 macrophages have garnered significant attention in SCI treatment due to their anti‐inflammatory properties and tissue repair potential. Among these, the M2c subtype demonstrates distinct advantages in early‐stage repair by secreting high levels of TGF‐β and clearing apoptotic cells.^[^ ^7^ ^]^ GNA12 and GNA13, as pivotal G protein subunits, induce macrophage polarization toward the M2c phenotype by upregulating TGF‐β expression through the activation of the Rho/Rac‐dependent AP‐1 signaling pathway.^[^ ^8^ ^]^

Exosomes, as natural nanoscale carriers, hold great promise for SCI treatment due to their excellent biocompatibility and immunomodulatory properties. However, traditional delivery methods, such as intravenous injection, are often limited by poor targeting efficiency and low bioavailability, which pose significant challenges for therapeutic applications.

### 1.3 Chitosan‐Based Thermosensitive Hydrogel Nasal Spray

**Scheme 1:**
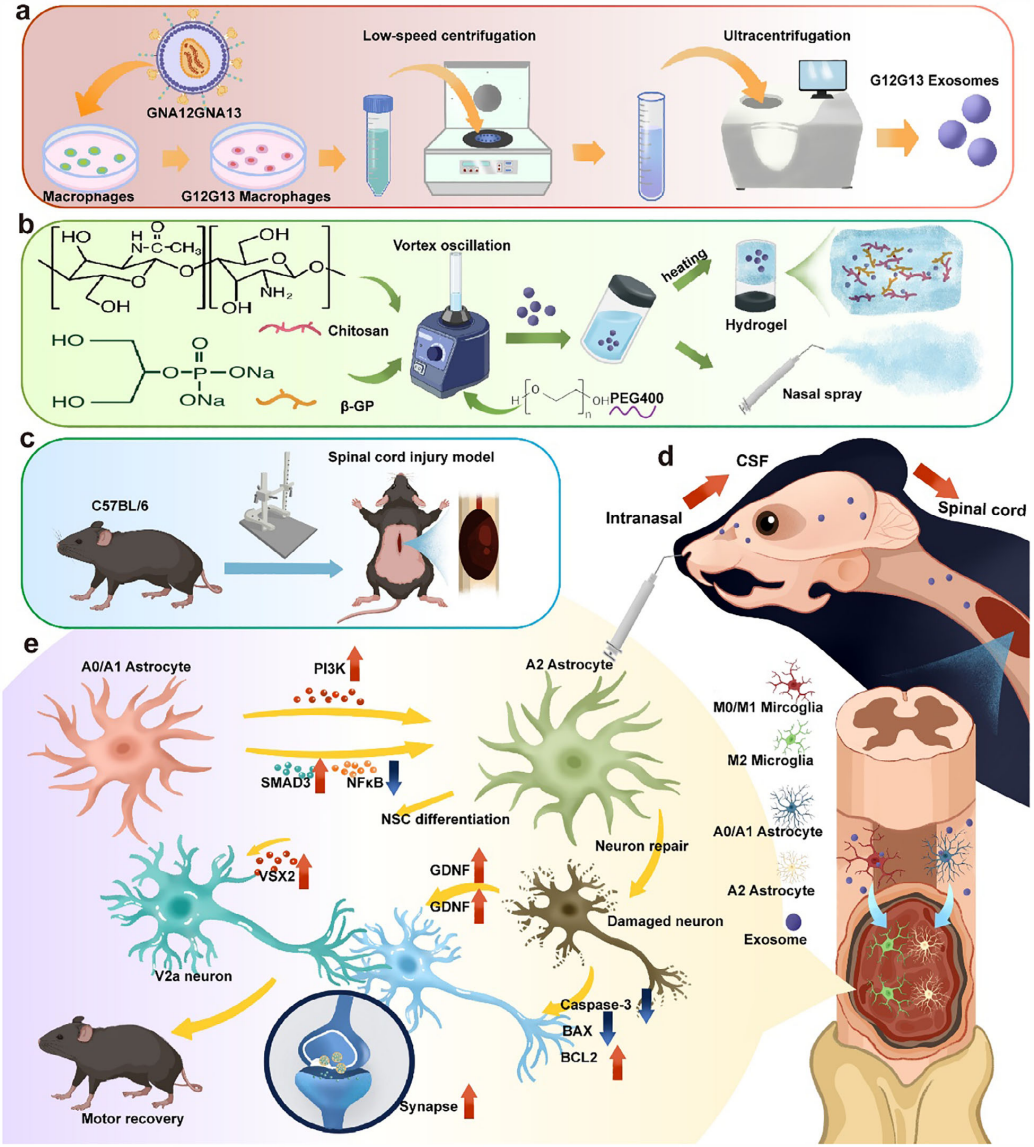
Nasal Delivery of Engineered Exosomes via a Thermo‐Sensitive Hydrogel Depot Reprograms Glial Cells for Spinal Cord Repair a) Isolation of G12G13MExos. b) Fabrication of the thermosensitive hydrogel. c) Establishment of the SCI model. d) Intranasal administration of G12G13MExos‐hydrogel. e) Therapeutic mechanism of G12G13MExos.

Intranasal administration is a non‐invasive approach that bypasses the BSCB, delivering therapeutics directly to the central nervous system via nasal mucosal diffusion and neuronal pathways.^[^ ^9^ ^]^ However, exosome delivery via the nasal route faces challenges, such as poor stability and limited retention time in the nasal cavity.^[^ ^10^ ^]^ This study developed a thermosensitive chitosan‐based hydrogel nasal spray that forms an “intelligent sustained‐release reservoir” on the nasal mucosa to address these issues. This system enables the controlled and sustained release of exosomes derived from GNA12/GNA13‐overexpressing macrophages, ensuring precise delivery (**Scheme** **1**). This strategy significantly enhances the repair of the injury site, offering a novel approach to SCI treatment by precisely modulating the glial microenvironment during the early stages of SCI.

## 2 Results and Discussion

### 2.1 Exosomes from GNA12/GNA13‐Overexpressing Macrophages Promote Neuroprotective Glial Phenotypes

**Figure 1:**
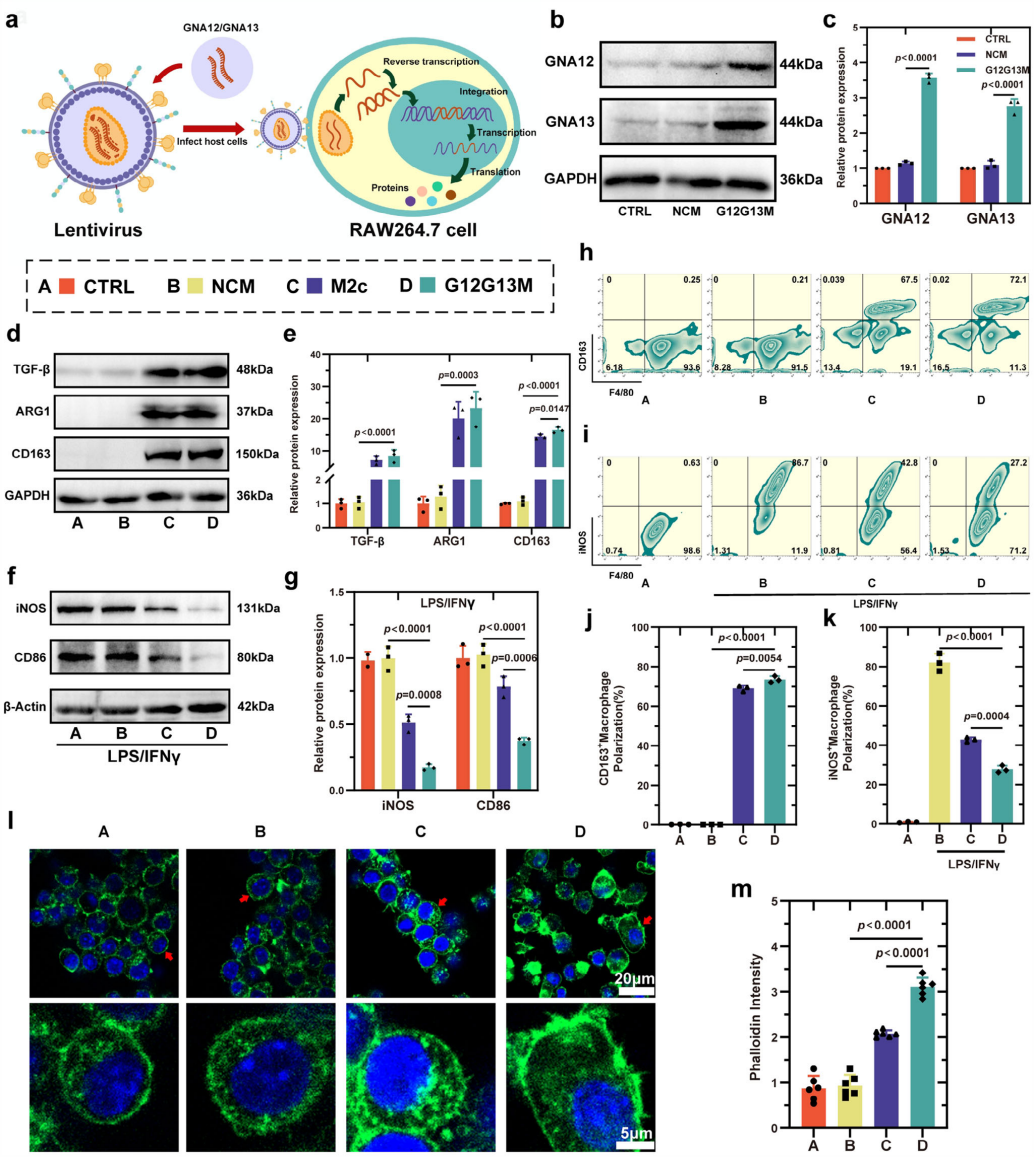
Lentivirus‐mediated GNA12/13 overexpression induces M2c macrophage polarization and cytoskeletal remodeling. a) Schematic illustration of lentiviral transduction of RAW264.7 macrophages with GNA12 and GNA13 to generate engineered M2c‐like G12G13M macrophages. b,c) Western blot and quantification of GNA12 and GNA13 protein expression in different macrophage groups (n = 3). d,e) Expression of M2c markers (TGF‐β, ARG1, CD163) under basal conditions, showing enhanced M2c polarization in G12G13M macrophages (n = 3). f,g) Expression of M1 markers (iNOS, CD86) under LPS/IFN‐γ stimulation, indicating suppressed M1 polarization in the G12G13M group (n = 3). h–k) Flow cytometry analysis and quantification of CD163⁺ M2c macrophages (h, j) and iNOS⁺ M1 macrophages (i, k) (n = 3). l) Immunofluorescence imaging of actin cytoskeleton (phalloidin, green) and nuclei (DAPI, blue), highlighting enhanced cytoskeletal organization in G12G13M macrophages. Scale bars: 20 µm (top), 5 µm (bottom). m) Quantification of phalloidin intensity (n = 6). Data are presented as mean ± SD. Statistical comparisons were performed using one‐way ANOVA followed by Tukey's multiple comparisons test. Exact *p*‐values are shown in the Figure.

The M2c macrophage subtype primarily contributes to suppressing inflammation and promoting tissue repair and can be induced by TGF‐β.^[^ ^11^ ^]^ In this study, RAW264.7 macrophages were transduced with lentiviruses to overexpress GNA12 and GNA13 (G12G13M group). Morphological analysis showed the characteristic features of GNA12GNA13‐overexpressing macrophages (Figure S1, Supporting Information). Successful gene and protein overexpression was confirmed by WB and qPCR analyses (*p* < 0.001) (**Figure** **1**a–c, Figure S2a, Supporting Information). CCK‐8 assays showed no significant differences in cell viability and proliferation between the G12G13M group, the negative control group (NCM), and the blank control group (CTRL), indicating the successful establishment of the overexpression cell line (Figure S2b, Supporting Information).

For polarization profiling, the G12G13M group exhibited significantly higher expression of M2c markers (TGF‐β, ARG1, and CD163) compared to the NCM group (*p* < 0.001) (Figure 1d,e). Following LPS/IFN‐γ stimulation, the G12G13M group showed substantially lower expression of M1 markers (iNOS and CD86) than both the NCM group and the TGF‐β‐induced M2c group (*p* < 0.001) (Figure 1f,g). These findings were further validated by qPCR, which demonstrated significantly upregulated expression of Tgfb1, Arg1, and Cd163 genes and minimal expression of Nos2 and Cd86 in the G12G13M group (*p* < 0.001) (Figure S2c,d, Supporting Information). Flow cytometry and ELISA analyses further confirmed the anti‐inflammatory properties and polarization stability of the G12G13M group (*p* < 0.001) (Figure 1h–k, Figure S2e,f, Supporting Information).

These results indicate that macrophages overexpressing GNA12 and GNA13 exhibit an M2c phenotype with enhanced stability under inflammatory stimulation, surpassing that of M2c macrophages induced solely by TGF‐β. This enhanced stability may be attributed to their autocrine secretion of high levels of TGF‐β, providing a mechanistic basis for their superior role in inflammation regulation.

Previous studies identified Rho and Rac as key downstream effectors of GNA12‐ and GNA13‐mediated AP‐1 activation.^[^ ^8^ ^]^ Rho family GTPases, such as RhoA and RAC1, play critical roles in regulating cytoskeletal rearrangements and growth cone behavior, significantly promoting the formation of macrophage pseudopodia and stress fibers, thereby enhancing their motility and directional migration.^[^ ^12^ ^]^ Notably, in inflammatory microenvironments, activation of the Rho/Rac pathway improves macrophage responsiveness to chemokine signaling, accelerating their migration to inflammatory sites to exert immunomodulatory and reparative functions.

We conducted a series of experiments to assess the migratory capacity and underlying mechanisms of macrophages overexpressing GNA12 and GNA13. In Transwell assays, the number of migrated macrophages in the G12G13M group was significantly higher than in the NCM and M2c groups (*p* < 0.001) (Figure S3a,b, Supporting Information). Scratch assays similarly showed that the migration rate of the G12G13M group was significantly higher than that of the other groups (*p* < 0.001) (Figure S3c,d, Supporting Information). Confocal microscopy revealed that G12G13M macrophages exhibited well‐organized, dense F‐actin fibers and prominent pseudopodia formation, with cytoskeletal features significantly enhanced compared to the NCM and M2c groups (*p* < 0.001) (Figure 1l,m). These results confirm the enhanced migratory capacity of G12G13M macrophages at both functional and structural levels, providing a foundation for investigating their roles in complex physiological environments.

Exosomes were extracted using ultracentrifugation and subsequently characterized. The results demonstrated that exosomes from all groups exhibited standard morphological features, with a particle size of 147.9 ± 4.45 nm and a zeta potential of −10.73 ± 0.29 mV. These characteristics showed high uniformity and stability (Figure S4a–c, Supporting Information). WB and Coomassie brilliant blue staining further confirmed the presence of exosomal markers and protein composition (Figure S4d,e, Supporting Information). Since the biological activity of exosomes is closely tied to their source cells, exosomes from different groups may carry distinct molecules that exert specific regulatory effects on recipient cells. WB analysis revealed that TGF‐β expression in G12G13MExos was significantly higher than that in NCMExos (*p* < 0.001), confirming the effective loading of overexpressed proteins into exosomes (Figure S5a,b, Supporting Information).

**Figure 2:**
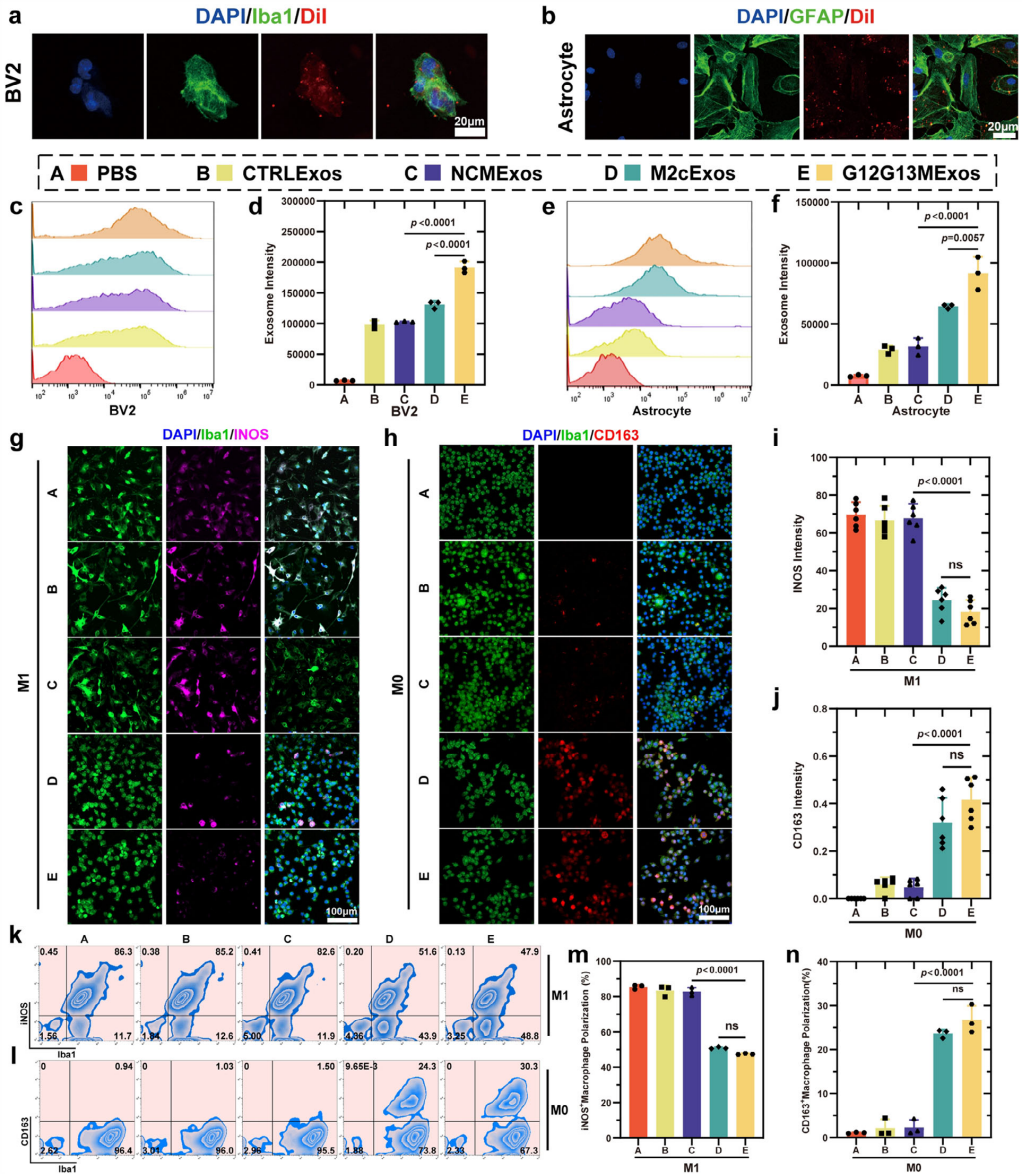
G12G13MExos are efficiently internalized by glial cells and reprogram microglial phenotypes. a, b) Confocal imaging of DiI‐labeled exosomes (red) taken up by BV2 microglia (a) and primary astrocytes (b). Microglia were co‐stained with Iba1 (green) and DAPI (blue); astrocytes were stained with GFAP (green) and DAPI. Scale bars: 20 µm. c–f) Flow cytometry analysis and quantification of exosome uptake in BV2 microglia (c, d) and astrocytes (e, f). The G12G13MExos group showed significantly enhanced internalization efficiency (n = 3). g,h) Immunofluorescence staining of microglia under M1‐polarizing (g) and M0 (baseline) conditions (h). M1 polarization was assessed via iNOS (magenta), and M2c polarization via CD163 (red). Iba1 (green) and DAPI (blue) were used for microglial identification and nuclear staining. Scale bars: 100 µm. i,j) Quantification of iNOS (i) and CD163 (j) fluorescence intensity in microglia, indicating that G12G13MExos inhibit M1 polarization and promote M2c transition (n = 6). k,l) Flow cytometry plots showing the percentage of iNOS⁺ (k) and CD163⁺ (l) microglia across different treatment groups. m,n) Quantification of M1 (iNOS⁺) and M2c (CD163⁺) microglial populations from flow cytometry analysis (n = 3), confirming the modulatory effect of G12G13MExos on microglial phenotypes. Data are presented as mean ± SD. Statistical analysis was performed using one‐way ANOVA followed by Tukey's multiple comparisons test. Exact *p*‐values are shown in the Figure ns, not significant.

Astrocytes isolated from the cerebral cortex of C57BL/6 mice were characterized using the astrocyte marker GFAP (Figure S6, Supporting Information). Confocal laser scanning microscopy demonstrated that both BV2 microglial cells and primary astrocytes effectively internalized exosomes (**Figure** **2**a,b). Flow cytometry analysis revealed that exosome uptake by BV2 cells and astrocytes was significantly higher in the G12G13M group than in the NCM group (*p* < 0.001), with the G12G13M exosomes exhibiting superior phagocytic efficiency over other experimental groups (Figure 2c–f). These findings indicate enhanced cellular targeting specificity of G12G13M exosomes.

To further investigate the mechanisms underlying this enhanced uptake, we examined the role of RhoA signaling, given its established function in regulating cytoskeletal dynamics and vesicle internalization. Confocal imaging revealed that treatment with the RhoA‐specific inhibitor Rhosin markedly disrupted F‐actin organization in astrocytes, reducing cytoskeletal integrity (*p* < 0.001) (Figure S7a,b, Supporting Information). Flow cytometry analysis further confirmed that Rhosin significantly decreased the internalization of G12G13MExos by astrocytes (*p* < 0.001) (Figure S7c,d, Supporting Information), suggesting that the elevated uptake of these engineered exosomes is at least partially mediated by RhoA‐dependent endocytosis.

These results support the notion that GNA12 and GNA13 proteins, which were enriched within G12G13MExos and delivered to recipient cells, may activate intracellular RhoA signaling, thereby promoting actin remodeling and facilitating exosome uptake. This “delivery–activation–reuptake” feedback mechanism represents a distinctive functional enhancement of engineered exosomes, contributing to their superior cellular internalization and therapeutic potential.

Transwell co‐culture experiments were conducted to assess the inflammatory tropism of exosomes (Figure S8b, Supporting Information). The fluorescence intensity of Dil‐labeled exosomes was significantly higher in both BV2 cells and astrocytes in the G12G13M group than in the NCM group (*p* < 0.001). Additionally, the fluorescence intensity in astrocytes was significantly higher than that in the M2c group (*p* < 0.001). However, in BV2 cells, it was marginally higher than in the M2c group (*p* = 0.0147) (Figure S8a,c,d, Supporting Information). These results indicate that G12G13M exosomes exhibit stronger inflammatory tropism, with faster and more robust responses to inflammatory stimuli.

The ability of exosomes to regulate target cell phenotypes by delivering bioactive factors has been widely documented, with microglial/macrophage polarization being a typical example.^[^ ^13^, ^14^ ^]^ In this study, WB analysis showed that G12G13M exosomes significantly enhanced the reversal of the M1 phenotype (*p* < 0.001) and promoted the transition of the M0 phenotype to the M2c phenotype (*p* < 0.001) (Figure S9, Supporting Information). Immunofluorescence and flow cytometry further confirmed the regulatory effects of G12G13M exosomes, demonstrating a marked reduction in iNOS protein fluorescence intensity (*p* < 0.001) and a significant increase in CD163 protein fluorescence intensity (*p* < 0.001) alongside a reduction in reactive oxygen species levels (*p* < 0.001) (Figure 2g–n, Figure S10, Supporting Information). Given the high expression of TGF‐β in G12G13M exosomes and its established role in inducing M2c polarization, we hypothesize that G12G13M exosomes promote M2c polarization of microglia via TGF‐β transfer.^[^ ^15^ ^]^

**Figure 3:**
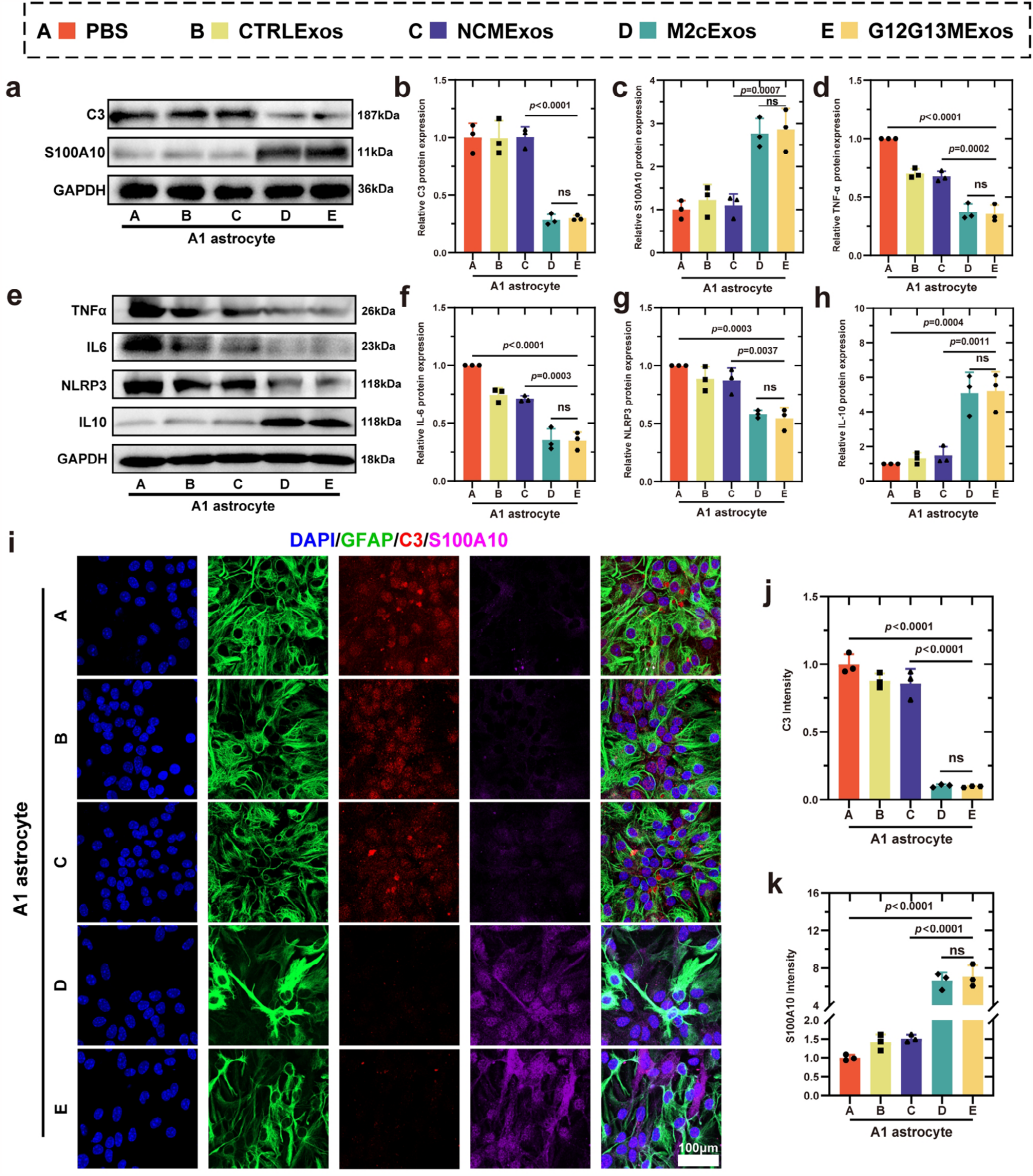
G12G13MExos reverse the neurotoxic A1 astrocyte phenotype and promote a neuroprotective state. a–c) Western blot analysis and quantification of C3 (A1 neurotoxic marker) and S100A10 (neuroprotective marker) in A1 astrocytes treated with different exosome groups. G12G13MExos treatment significantly suppressed C3 and upregulated S100A10 expression (n = 3). d–h) Western blot analysis and quantification of inflammatory mediators (TNF‐α, IL‐6, NLRP3) and the anti‐inflammatory cytokine IL‐10 in A1 astrocytes. G12G13MExos markedly attenuated pro‐inflammatory signaling while enhancing IL‐10 expression (n = 3). i) Immunofluorescence staining of A1 astrocytes showing co‐localization of GFAP (green), C3 (red), and S100A10 (magenta). Nuclei were labeled with DAPI (blue). G12G13MExos markedly reduced C3 expression and enhanced S100A10 accumulation. Scale bar: 100 µm. j,k) Quantification of C3 (j) and S100A10 (k) fluorescence intensity confirmed that G12G13MExos effectively reprogrammed A1 astrocytes toward a neuroprotective phenotype (n = 3). Data are presented as mean ± SD. Statistical analysis was performed using one‐way ANOVA followed by Tukey's multiple comparisons test. Exact *p*‐values are shown in the Figure ns, not significant.

G12G13M exosomes significantly promote the phenotypic transition of neurotoxic astrocytes to a neuroprotective phenotype. In an inflammation‐induced neurotoxic astrocyte model, C3 protein expression was markedly upregulated (*p* < 0.001), confirming the transition to the A1 phenotype (Figure S11a,b, Supporting Information).^[^ ^16^ ^]^ WB analysis revealed that G12G13M exosomes significantly reduced C3 protein levels (*p* < 0.001) while increasing S100A10 protein expression (*p* < 0.001), demonstrating a superior effect in promoting the transition from the A1 to the A2 phenotype (**Figure** **3**a–c). These findings were further corroborated by immunofluorescence analysis (*p* < 0.001) (Figure 3i–k). Regarding the inflammatory microenvironment, G12G13M exosomes significantly reduced the expression of pro‐inflammatory factors TNF‐α, IL‐6, and NLRP3 (*p* < 0.001) while increasing the expression of the anti‐inflammatory cytokine IL‐10 (*p* < 0.001) (Figure 3d–h). ELISA results confirmed a decrease in inflammatory protein secretion (*p* < 0.001) (Figure S11c–e, Supporting Information). Additionally, ROS assays demonstrated a significant reduction in oxidative stress levels following treatment with G12G13M exosomes (*p* < 0.001) (Figure S11f,g, Supporting Information). Functional experiments showed that G12G13M exosomes enhanced the migratory capacity of astrocytes (*p* < 0.001) (Figure S12, Supporting Information), providing further evidence of their role in supporting neural repair and remodeling.

**Figure 4:**
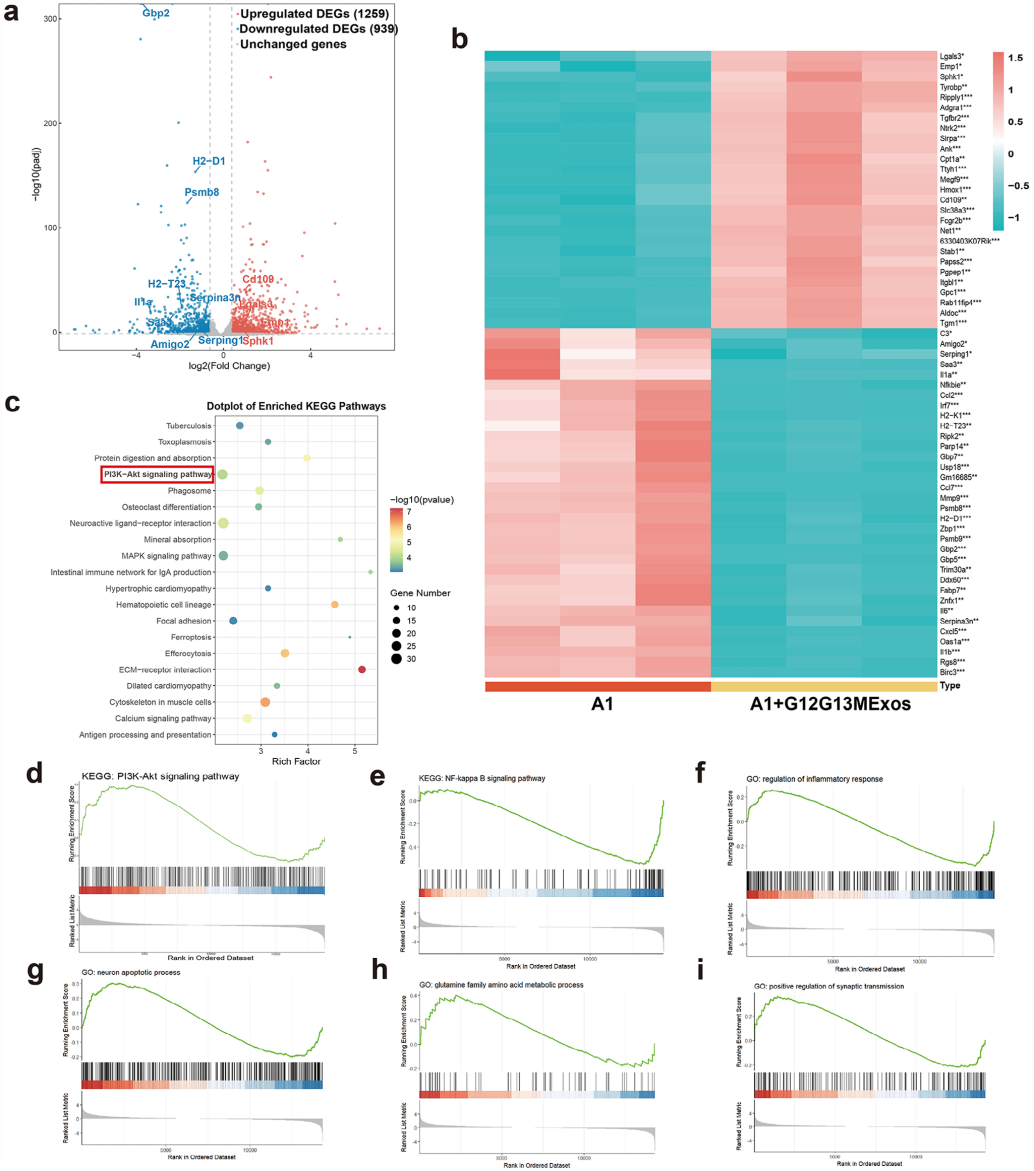
Transcriptomic analysis reveals that G12G13MExos modulate A1 astrocyte polarization via PI3K‐AKT and NF‐κB signaling pathways. a) Volcano plot showing DEGs between untreated A1 astrocytes and those treated with G12G13MExos. Red: upregulated genes; blue: downregulated genes; gray: unchanged genes. b) Heatmap of representative DEGs involved in inflammation, neuroprotection, and metabolism, demonstrating transcriptomic reprogramming in G12G13MExos‐treated astrocytes. c) KEGG enrichment analysis showing significant pathway activation in G12G13MExos‐treated astrocytes. PI3K‐AKT signaling was prominently enriched (highlighted in red). d–i) GSEA further confirmed activation of: (d) PI3K‐AKT signaling pathway, (e) NF‐κB signaling pathway, (f) Regulation of inflammatory response, (g) Neuronal apoptotic process, (h) Glutamine metabolic process, and (i) Synaptic transmission regulation. These data support the neuroprotective and anti‐inflammatory effects of G12G13MExos in A1 astrocyte modulation.

Through transcriptomic analysis and mechanistic validation, we uncovered the molecular mechanisms by which G12G13M exosomes induce the phenotypic transition of neurotoxic astrocytes to a neuroprotective phenotype. Exosome treatment significantly altered gene expression profiles and signaling pathway activities in neurotoxic astrocytes. A volcano plot highlighted the distribution of differentially expressed genes (DEGs). In contrast, KEGG analysis revealed that upregulated genes were significantly enriched in the PI3K‐AKT signaling pathway, and downregulated genes were enriched in the NF‐κB signaling pathway. These findings suggest that exosomes promote phenotypic transition by activating repair and anti‐inflammatory pathways while suppressing inflammatory signaling (**Figure** **4**a,b, Figure S13a, Supporting Information). GSEA further demonstrated robust activation of the PI3K‐AKT pathway, enhancing cell proliferation and survival (Figure 4d), as well as significant downregulation of the NF‐κB pathway, suppressing inflammatory signals (Figure 4e). Exosomes also enhanced anti‐inflammatory regulatory functions (Figure 4f) and inhibited neuronal apoptosis (Figure 4g). Metabolic pathway analysis showed significant activation of glutamate metabolism and myelin lipid metabolism (Figure 4h, Figure S13b, Supporting Information), along with upregulation of pathways related to synaptic transmission and neural progenitor cell proliferation (Figure 4i, Figure S13c, Supporting Information). Collectively, these results demonstrate that G12G13M exosomes improve the transcriptomic characteristics of neurotoxic astrocytes by regulating anti‐inflammatory, neuroprotective, metabolic, and repair‐associated pathways, providing critical evidence for their application in alleviating neuroinflammation.

The TGF‐β/SMAD and PI3K‐AKT signaling pathways play pivotal roles in astrocyte phenotypic transition. WB analysis showed that the TGF‐β pathway inhibitor SB‐431542 significantly reversed astrocyte phenotypic transition, evidenced by decreased p‐PI3K p110β levels (*p* = 0.01) and increased p‐NF‐κB p65 levels (p = 0.0048) (Figure S14a–g, Supporting Information). These findings suggest that G12G13M exosomes facilitate the phenotypic transition through activation of the TGF‐β pathway, which is dependent on the coordinated regulation of PI3K and NF‐κB signaling. Further validation showed that siRNA‐mediated inhibition of the PI3K‐AKT pathway (p110β siRNA) reversed the expression of C3 and S100A10 proteins and significantly reduced p‐PI3K p110β and p‐AKT1 levels (*p* < 0.001) (Figure S14h–k, Supporting Information), confirming the central role of the PI3K‐AKT pathway in phenotypic transition. Inhibition of the NF‐κB pathway using p65 siRNA significantly decreased C3 expression (*p* = 0.0082) while increasing S100A10 expression (*p* = 0.001) (Figure S15a–d, Supporting Information). Moreover, suppression of the TGF‐β/SMAD pathway via SMAD3 siRNA markedly reversed the phenotypic transition, as shown by increased C3 expression (*p* = 0.0002), decreased S100A10 expression (*p* = 0.0092), and restored levels of IκBα and p‐NF‐κB p65 (*p* < 0.0001) (Figure S15e–i, Supporting Information). These results indicate that G12G13M exosomes suppress NF‐κB signaling by activating the TGF‐β/SMAD pathway, a process dependent on SMAD3 phosphorylation. Additionally, the cooperative regulation of PI3K‐AKT and NF‐κB pathways plays a critical role in the phenotypic transition, providing a molecular basis for the exosome‐mediated modulation of astrocyte phenotypes.

To further evaluate the sustained regulatory potential of G12G13MExos, we treated astrocytes with equal amounts of G12G13MExos or TGFβ⁺M2cExos (derived from TGF‐β–enriched M2c macrophages), and monitored key signaling molecules over time. At Day 1 (24 h post‐treatment), both groups exhibited comparable levels of TGF‐β expression and Smad3 phosphorylation. However, by Days 3 and 5, astrocytes exposed to G12G13MExos maintained significantly higher levels of both TGF‐β and p‐Smad3 compared to the TGFβ⁺M2cExos group (*p* = 0.0184 and *p* = 0.005) (Figure S16a–c, Supporting Information), indicating a more sustained activation of the TGF‐β/Smad signaling axis.

In addition, the expression of RhoA—a downstream effector in the Rho/Rac signaling pathway—was markedly upregulated in the G12G13MExos group (*p* < 0.0001) (Figure S16d,e, Supporting Information). Considering the confirmed enrichment of GNA12 and GNA13 proteins in these exosome, we propose that G12G13MExos not only deliver extracellular TGF‐β but also introduce intracellular signaling mediators capable of persistently activating endogenous signaling cascades within recipient astrocytes. This dual‐modality mechanism promotes a self‐sustained loop of TGF‐β production and pathway activation, supporting prolonged phenotypic modulation and establishing a foundation for advanced exosome‐based reprogramming strategies.

### 2.2 Astrocyte‐Neuron Crosstalk Facilitated by Exosome Therapy Enhances Synaptic Formation

The crosstalk between astrocytes and neurons is critical for maintaining central nervous system (CNS) function and facilitating repair, particularly after SCI, where balance is essential for microenvironmental regulation and functional recovery.^[^ ^11^ ^]^ Under normal conditions, astrocytes support neuronal survival by secreting neurotrophic factors such as BDNF and GDNF and regulating synaptic homeostasis and plasticity to promote neural network remodeling.^[^ ^12^ ^]^ Following SCI, neurotoxic astrocyte activation causes the excessive release of inflammatory factors such as complement C3 and TNF‐α, exacerbating neuronal apoptosis and synaptic damage, which hinders functional recovery. Investigating the role of G12G13M exosome‐treated astrocytes in astrocyte‐neuron crosstalk may elucidate their molecular mechanisms in SCI repair.

**Figure 5:**
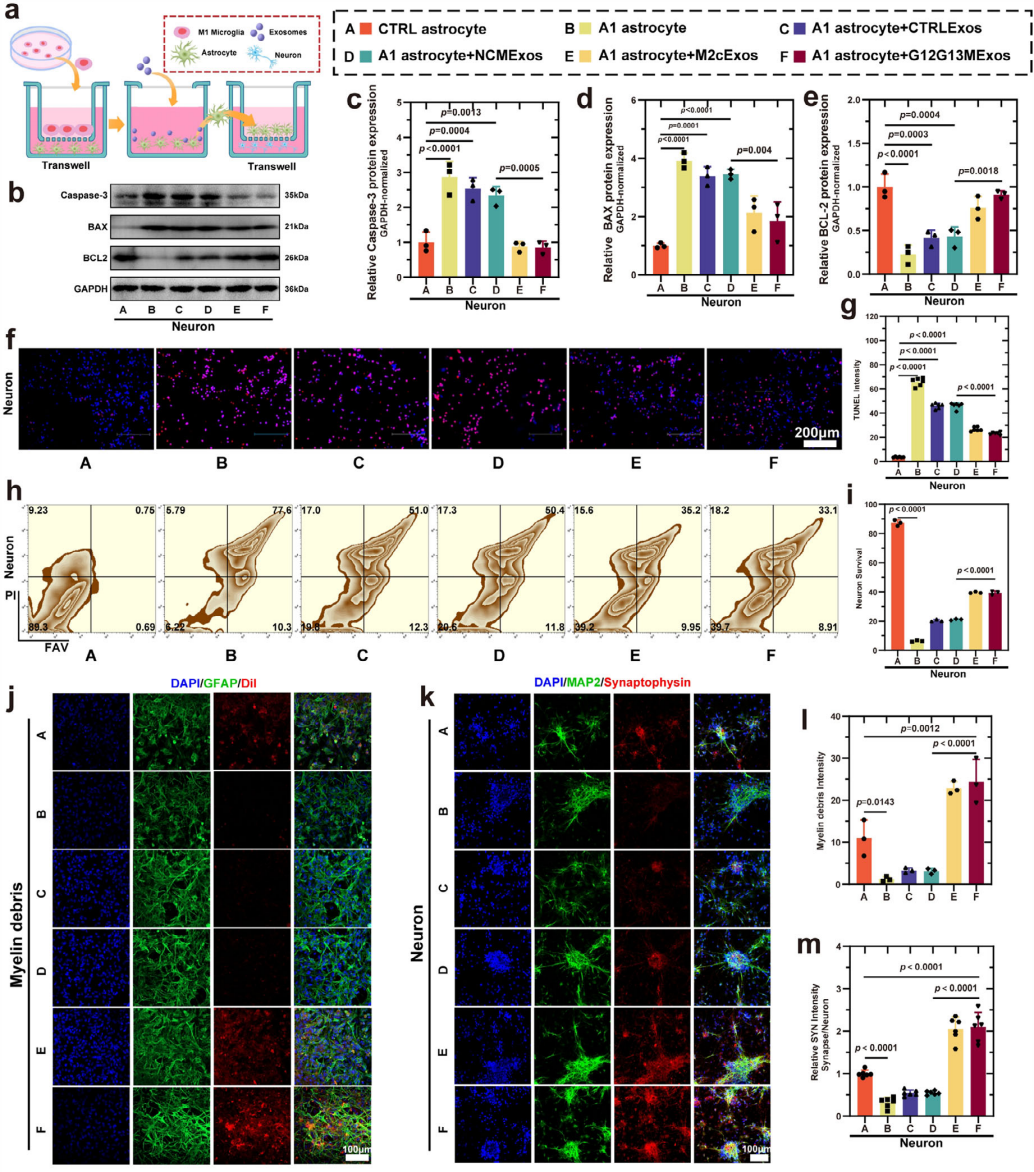
G12G13MExos‐pretreated A1 astrocytes promote neuronal survival and synaptic restoration via paracrine signaling. a) Schematic diagram of the Transwell co‐culture system used to model astrocyte–neuron communication under different exosome treatments. b) Western blot analysis of apoptosis‐related proteins (Caspase‐3, BAX, BCL2) in neurons co‐cultured with A1 astrocytes pretreated by different exosomes. c–e) Quantification of Caspase‐3, BAX, and BCL2 levels shows that neurons co‐cultured with G12G13MExos‐pretreated A1 astrocytes (Group F) exhibit significantly reduced apoptosis markers and increased anti‐apoptotic BCL2 (n = 3). f) Representative TUNEL staining of neurons showing reduced apoptosis in the G12G13MExos group. Scale bar: 200 µm. g) Quantification of TUNEL‐positive cells confirms reduced neuronal apoptosis upon co‐culture with G12G13MExos‐pretreated astrocytes (n = 6). h) Flow cytometry analysis of neuronal apoptosis under co‐culture with differently treated astrocytes. i) Quantification of apoptotic neuronal populations, with the lowest rate observed in the G12G13MExos group (n = 3). j) Immunofluorescence staining of astrocytes showing internalized myelin debris (red, DiI‐labeled), GFAP (green), and nuclei (blue). Astrocytes pretreated with G12G13MExos show enhanced debris phagocytosis. Scale bar: 100 µm. k) Immunofluorescence staining of neurons for MAP2 (green, dendrites) and synaptophysin (red, presynaptic terminals) after co‐culture with treated astrocytes. G12G13MExos‐pretreated astrocytes preserved neuronal structure and synaptic markers. Scale bar: 100 µm. l) Quantification of DiI‐labeled myelin debris within astrocytes (n = 3). m) Quantification of synaptophysin intensity in neurons, demonstrating enhanced synaptic integrity after co‐culture with G12G13MExos‐pretreated astrocytes (n = 6). Data are presented as mean ± SD. Statistical analysis was performed using one‐way ANOVA followed by Tukey's multiple comparisons test. Exact *p*‐values are shown in the Figure.

Using a Transwell co‐culture system (**Figure** **5**a), neurons isolated from the cerebral cortex of C57BL/6 mice were characterized using NEUN and MAP2 markers (Figure S17, Supporting Information). Under simulated secondary inflammatory conditions post‐SCI, WB analysis revealed that neurotoxic astrocytes induced significant neuronal apoptosis. However, treatment of neurotoxic astrocytes with G12G13M exosomes markedly reduced the expression of Caspase‐3 (*p* < 0.001) and BAX (*p* = 0.004), while upregulating BCL2 expression (*p* = 0.0018) (Figure 5b–e), indicating that G12G13M exosome‐treated astrocytes significantly attenuated neuronal apoptosis. This conclusion was further supported by TUNEL staining (Figure 5f,g) and Annexin V‐FITC/PI flow cytometry analysis (*p* < 0.001) (Figure 5h,i, Figure S18, Supporting Information).

ELISA results demonstrated that G12G13M exosome treatment significantly increased the secretion of BDNF and GDNF by astrocytes (*p* < 0.001), effectively restoring the neurotrophic factor expression impaired in neurotoxic astrocytes (Figure S19a,b, Supporting Information). BDNF and GDNF are well‐documented regulators of neuronal apoptosis through activation of the GDNF/GFRα1/RET and PI3K/AKT/GSK3β signaling pathways.^[^ ^13^, ^14^ ^]^ This study further confirms that G12G13M exosomes improve the pathological characteristics of astrocytes and restore their neurotrophic function, providing anti‐apoptotic support for neurons.

Excessive glutamate release following SCI leads to neuronal overstimulation, calcium influx, and reactive oxygen species generation, ultimately resulting in neuronal death and exacerbated injury.^[^ ^15^ ^]^ Reducing glutamate‐induced excitotoxicity is, therefore, a key focus of neuroprotection research. This study revealed that the glutamate clearance capacity of neurotoxic astrocytes (A1 astrocyte group) was significantly reduced (*p* < 0.001). In contrast, treatment with G12G13M exosomes significantly enhanced this capacity, with a marked improvement compared to the A1 astrocyte+NCMExos group (*p* < 0.0001) (Figure S19c, Supporting Information). Mechanistic analysis showed that this effect was closely associated with the upregulation of EAAT1 and EAAT2 expression (*p* < 0.0001) (Figure S19d–f, Supporting Information). EAAT1 and EAAT2 play critical roles in clearing extracellular glutamate, maintaining homeostasis, and protecting neurons from excitotoxic damage.^[^ ^16^ ^]^ Inflammatory environments are known to suppress EAAT1 and EAAT2 expression, thereby exacerbating neuronal injury.^[^ ^17^ ^]^ We hypothesize that G12G13M exosomes enhance the neuroprotective capacity of astrocytes by activating the PI3K/AKT signaling pathway to upregulate EAAT1 and EAAT2.^[^ ^18^ ^]^ These findings provide new insights into the pathological mechanisms of SCI and establish a theoretical basis for the application of exosomes in neuroprotection.

Contusive SCI results in significant demyelination and the generation of large amounts of myelin debris, which hinders axonal regeneration and exacerbates the inflammatory response.^[^ ^19^ ^]^ Timely clearance of myelin debris is crucial for regulating the inflammatory microenvironment, promoting axonal elongation, and facilitating remyelination. Astrocytes clear myelin debris through the endolysosomal system, with receptors such as LRP1, MEGF10, Mertk, ABCA1, and GULP1 playing critical roles.^[^ ^20^, ^21^, ^22^ ^]^ This study demonstrated that the myelin debris clearance capacity of neurotoxic astrocytes was significantly impaired. However, treatment with G12G13M exosomes restored and significantly enhanced this capacity (*p* < 0.0001) (Figure S20a, Supporting Information). Immunofluorescence analysis further validated these findings, showing a significant increase in astrocytic phagocytic activity following exosome treatment (*p* < 0.0001) (Figure 5j,l). Additionally, previous studies have shown that astrocytes and microglia can collaborate to enhance debris clearance efficiency.^[^ ^23^ ^]^ These results highlight the role of G12G13M exosomes in promoting myelin debris clearance and emphasize the importance of debris removal in SCI repair.

Astrocytes play a critical role in regulating the synaptic microenvironment and spinal cord neuronal synaptic transmission. As an integral part of the “tripartite synapse,” astrocytes maintain synaptic homeostasis by releasing or absorbing neurotransmitters and ions, and they contribute to synaptic formation and repair by secreting regulatory factors.^[^ ^24^, ^25^ ^]^ This study found that neurotoxic astrocytes significantly suppressed the expression of synaptogenesis‐related genes Thbs1 and Gpc6 (*p* < 0.0001) and reduced the expression of neuronal synaptic markers Synaptophysin and SYN1.^[^ ^26^ ^]^ However, treatment with G12G13M exosomes restored gene and protein expression levels (*p* < 0.001) (Figure S20b–e). Immunofluorescence analysis further confirmed the significant role of exosomes in promoting synapse formation (*p* < 0.001) (Figure 5k,m). These findings suggest that astrocytes treated with G12G13M exosomes play an important role in synaptogenesis and neural circuit reconstruction.

We hypothesize that G12G13M exosomes regulate synapse formation and neuronal connectivity through TGF‐β secreted by astrocytes. As a key factor in synaptogenesis, TGF‐β promotes both excitatory and inhibitory synapse formation and enhances synaptic stability and function by modulating the maturation and integration of pre‐ and postsynaptic components.^[^ ^27^, ^28^ ^]^ TGF‐β1 has been shown to significantly promote synapse formation, optimize dendritic spine morphology and density, and improve synaptic transmission efficiency between neurons.^[^ ^29^ ^]^ These effects are likely mediated through activation of the Smad signaling pathway upon binding to TGF‐β receptors, thereby regulating synaptic gene expression and protein function. These findings underscore the critical role of TGF‐β secreted by astrocytes in synaptic plasticity and neural repair, providing an important mechanism for maintaining neural network function.

### 2.3 Exosome‐Induced Astrocyte Modulation Promotes V2a Neuronal Differentiation

Neural circuit plasticity forms the foundation for functional recovery following SCI. SCI‐induced axonal transection and neuronal loss have led traditional repair strategies to focus primarily on promoting corticospinal tract (CST) axon regeneration and elongation. However, the restoration of distal connections remains a significant challenge due to the limited regenerative capacity of axons, the inhibitory microenvironment, and the long distances involved.^[^ ^30^ ^]^ The concept of a “neural bridge” offers a novel approach to SCI repair by leveraging newly generated intermediate neurons to establish long‐distance connections, linking the proximal ends of damaged axons to their targets. New neurons can be derived through exogenous transplantation or endogenous differentiation, with endogenous neural stem/progenitor cells (eNSPCs) emerging as promising candidates due to their safety and non‐invasive properties.^[^ ^31^ ^]^ eNSPCs, primarily originating from ependymal cells (EpCs), are rapidly activated and proliferate following SCI. However, under pathological conditions, ≈95% of eNSPCs differentiate into astrocytes, ≈5% into oligodendrocytes, and almost none into neurons.^[^ ^32^, ^33^ ^]^ Therefore, effectively inducing neuronal differentiation of eNSPCs is critical for neural circuit reconstruction.

**Figure 6:**
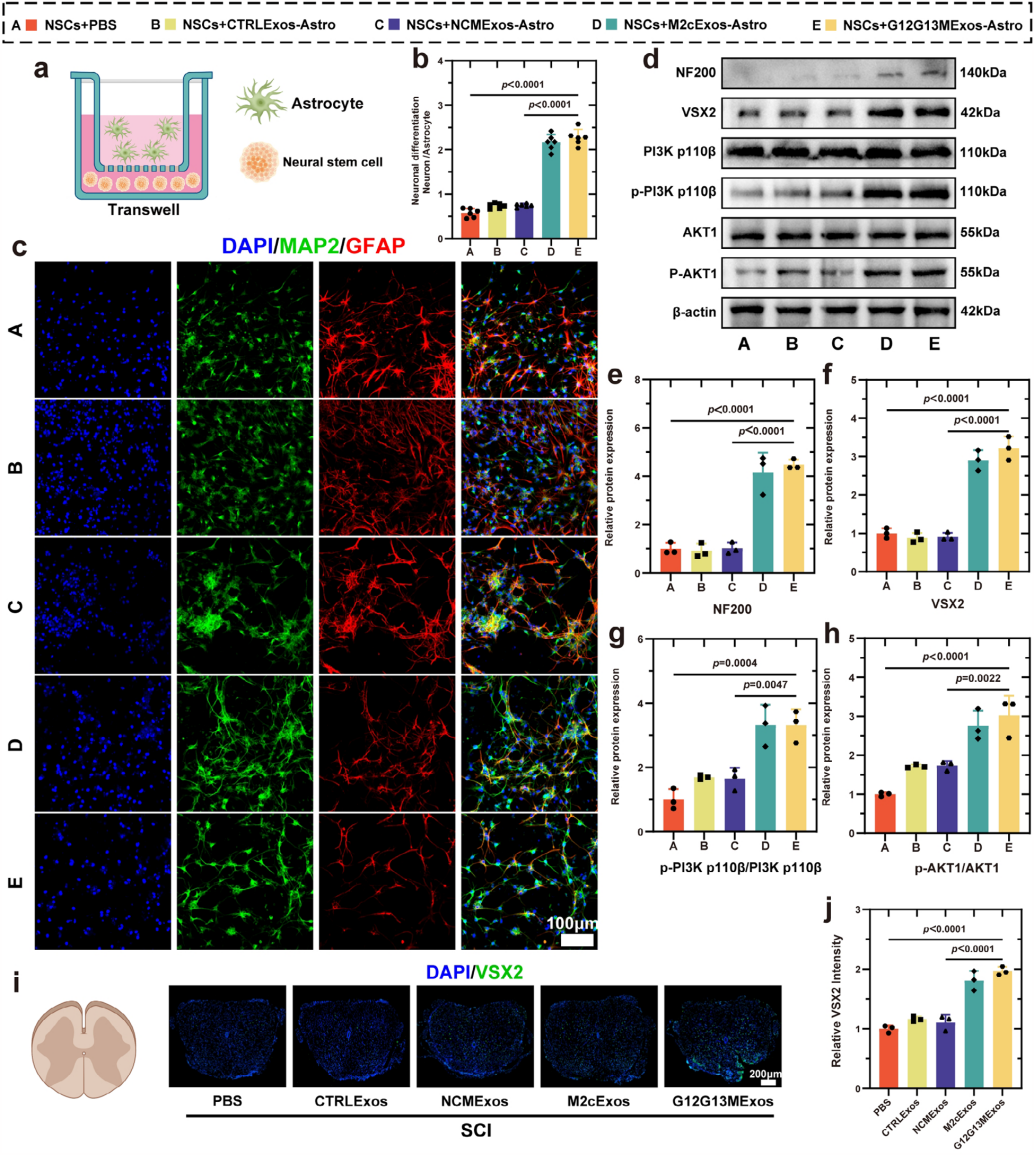
G12G13MExos promote NSC differentiation via activation of the PI3K‐AKT signaling pathway. a) Schematic illustration of the Transwell co‐culture system used to evaluate the influence of astrocytes treated with different exosome types on NSC differentiation. b) Quantification of neuronal differentiation efficiency, showing significantly enhanced differentiation in the G12G13MExos‐treated group (Group E) (n = 6). c) Representative immunofluorescence images of differentiated NSCs, stained for MAP2 (green, neuronal marker), GFAP (red, astrocyte marker), and DAPI (blue, nuclei). NSCs co‐cultured with G12G13MExos‐treated astrocytes exhibited increased neuronal differentiation. Scale bar: 100 µm. d) Western blot analysis of neuronal differentiation markers (NF200, VSX2) and key proteins in the PI3K‐AKT signaling cascade (PI3K p110β, p‐PI3K p110β, AKT1, p‐AKT1) across all groups. e–h) Quantification of Western blot results (n = 3). (e) NF200 expression. (f) VSX2 expression. (g) Phosphorylated PI3K p110β relative to total PI3K p110β. (h) Phosphorylated AKT1 relative to total AKT1. i) Representative immunofluorescence images of spinal cord sections collected on day 28 post‐SCI, stained for VSX2 (green, neural progenitor marker) and DAPI (blue). G12G13MExos treatment significantly enhanced VSX2 expression in vivo. Scale bar: 200 µm. j) Quantification of VSX2 fluorescence intensity in spinal cord sections, confirming increased NSC activation in the G12G13MExos‐treated group (n = 3). Data are presented as mean ± SD. Statistical analysis was performed using one‐way ANOVA followed by Tukey's multiple comparisons test. Exact *p*‐values are shown in the Figure.

The effect of G12G13M exosome‐treated astrocytes (G12G13MExos‐Astro) on neural stem cells was investigated using a Transwell co‐culture system along with EdU and CCK‐8 assays to assess neural stem cell proliferation (**Figure** **6**a). Neural stem cells were characterized using SOX2 and NESTIN markers (Figure S21, Supporting Information). Results demonstrated that G12G13M exosomes treatment significantly promoted neural stem cell proliferation and viability (*p* < 0.0001) (Figure S22a–c, Supporting Information). Under differentiation conditions, exosome treatment induced neural stem cells to differentiate into neurons while inhibiting astrocytic differentiation (*p* < 0.0001) (Figure 6b,c). These findings propose a novel strategy for neural circuit reconstruction by regulating neural stem cells via exosomes.

WB analysis further revealed that G12G13M exosome treatment significantly upregulated the expression of neuronal marker NF200 and V2a neuron‐specific marker VSX2 (*p* < 0.0001) (Figure 6d–h). V2a neurons, as glutamatergic intermediate neurons, transmit motor commands to motor neurons, thereby controlling somatic movement.^[^ ^34^ ^]^ G12G13M exosome‐treated astrocytes significantly promoted the differentiation of neural stem cells into V2a neurons through activation of the PI3K‐AKT signaling pathway (*p* = 0.0047 and *p* = 0.0022).

It is noteworthy that functional recovery post‐SCI relies on neural circuit reconstruction via descending axons and intermediate neurons for information transmission rather than precise axonal reconnection.^[^ ^35^ ^]^ Even when severed neurons successfully establish new connections, motor function recovery may remain limited.^[^ ^36^ ^]^ Recent work further demonstrated that thoracic Vsx2⁺ V2a interneurons with long‐projecting axons serve as critical relay elements bridging supraspinal input to lumbar circuits, enabling functional recovery even after complete SCI. Ablation of these neurons abolished spontaneous improvement, underscoring their essential role in locomotor repair.^[^ ^34^ ^]^

Additionally, Chx10⁺ V2a interneurons located in lamina VII have been shown to form glutamatergic excitatory connections with motor neurons, V0 inhibitory interneurons, and other relay neurons.^[^ ^37^ ^]^ These neurons also participate in central pattern generators (CPGs) that regulate gait rhythm and limb coordination. Their absence or impaired differentiation severely limits circuit reorganization and motor recovery.

Taken together, our findings suggest that the ability of G12G13M exosome‐treated astrocytes to enhance V2a neuron generation may represent a key mechanism by which this intervention promotes functional improvement after SCI. This cell‐type–specific circuit reconstruction adds mechanistic depth to the therapeutic potential of exosome‐mediated astrocytic modulation.

### 2.4 Optimized Hydrogel Delivery Overcomes Systemic Barriers

A contusive SCI model was successfully established in C57BL/6 mice using Allen's impact method (Figure S23, Supporting Information). Post‐injury, the mice exhibited hematomas, tissue depression, and complete hindlimb motor function loss, consistent with characteristics of severe SCI. These findings validated the reliability of the model and provided a stable foundation for subsequent research.

Currently, exosomes are predominantly delivered via intravenous (IV) or local injection. In this study, mice received either IV or intranasal administration of exosomes at a dose of 100 µg per day for three consecutive days following SCI. It was found that intranasal administration achieved superior targeting to the SCI site compared to IV injection (Figure 8a,b). IV injection faces limitations such as low bioavailability, poor targeting, and BSCB obstruction. In contrast, intranasal delivery bypasses the BSCB, achieving efficient targeting via diffusion through the nasal epithelium or transport along neuronal pathways.^[^ ^38^, ^39^ ^]^ Despite these advantages, intranasal delivery is constrained by limited nasal surface area and mucosal absorption efficiency. This study addresses these challenges by innovatively designing a “nasal exosome intelligent sustained‐release reservoir,” significantly enhancing exosome delivery efficiency and expanding its therapeutic potential for SCI repair.

**Figure 7:**
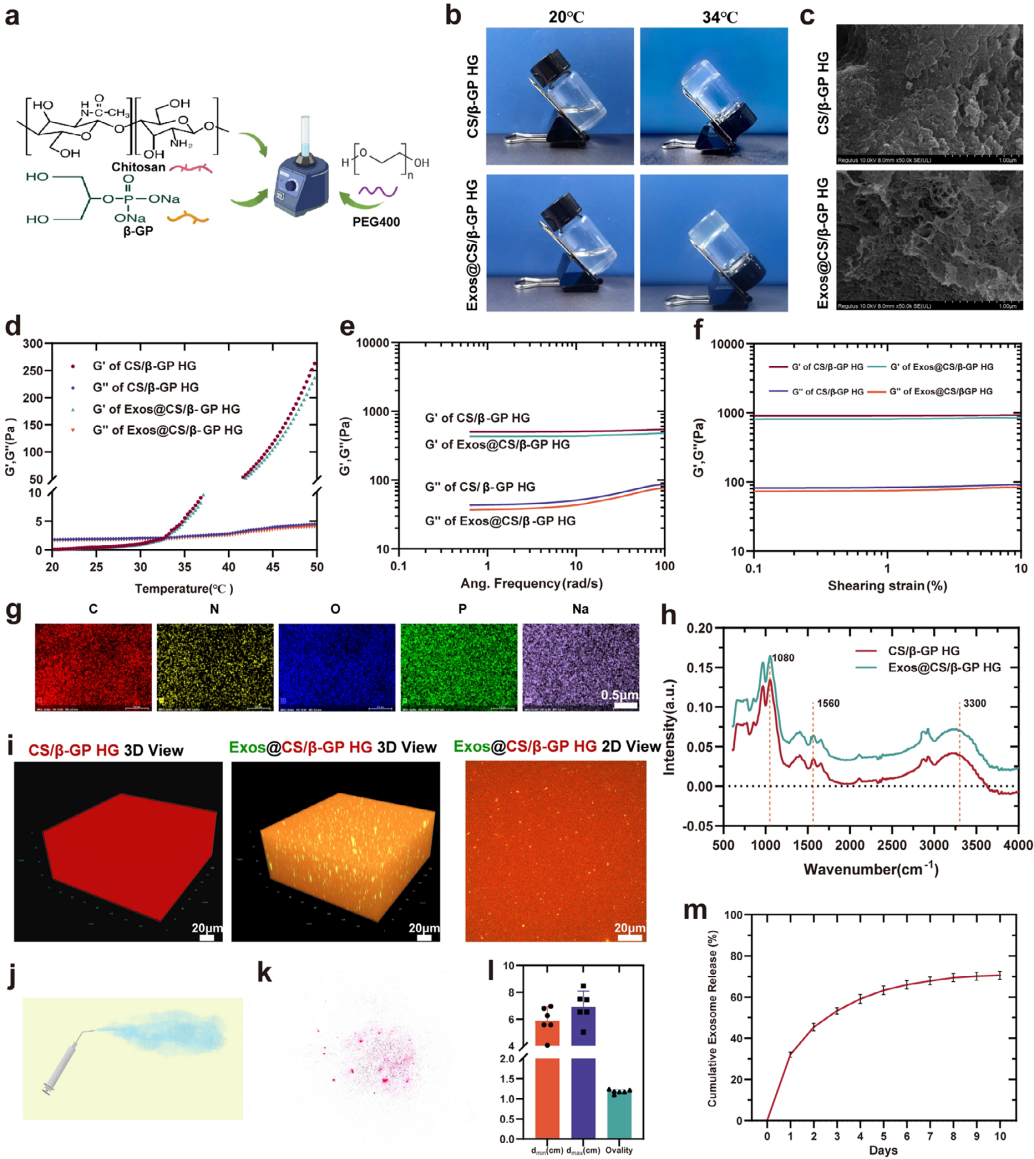
Characterization of PEG400‐modified CS/β‐GP thermosensitive hydrogel and its exosome encapsulation and sustained‐release performance. a) Schematic illustration of hydrogel synthesis via thermosensitive gelation of chitosan (CS) and β‐glycerophosphate (β‐GP), modified with PEG400 to enhance fluidity and atomization. b) Photographs of the hydrogel at 20 °C and 34 °C demonstrating temperature‐induced sol–gel transition. c) SEM images of lyophilized CS/β‐GP and Exos@CS/β‐GP hydrogels, showing uniform porous microstructures. Scale bar: 1 µm. d–f) Rheological analysis comparing CS/β‐GP and Exos@CS/β‐GP hydrogels (n = 3): (d) Temperature sweep curves showing sharp increases in storage (G′) and loss (G″) moduli upon reaching gelation temperature. (e) Frequency sweep indicates stable mechanical properties under oscillatory shear. (f) Shear strain sweep demonstrates good viscoelastic behavior across a wide strain range. g) Elemental mapping (C, N, O, P, Na) of Exos@CS/β‐GP confirms uniform elemental distribution throughout the hydrogel matrix. Scale bar: 0.5 µm. h) FTIR spectra reveal characteristic peaks of hydrogen bonding (≈3300 cm⁻¹) and phosphate interactions (≈1080, 1560 cm⁻¹), supporting hydrogel network formation. i) Confocal 3D and 2D fluorescence imaging of DiO‐labeled exosomes within the hydrogel, indicating uniform distribution and effective encapsulation. Scale bar: 20 µm. j) Schematic of nasal administration illustrating smooth extrusion of the hydrogel formulation through a microspray device. k) Representative atomization image of sprayed hydrogel droplets, showing fine dispersion. l) Quantitative analysis of droplet maximum diameter (dmax), minimum diameter (dmin), and ovality before and after exosome incorporation (n = 6). m) Cumulative release profile of exosomes under simulated nasal cavity conditions (pH 6.0, 34 °C), demonstrating sustained exosome release over 7 days (n = 3). Data are presented as mean ± SD.

The chitosan/β‐glycerophosphate (CS/β‐GP) thermosensitive hydrogel developed in this study was optimized by incorporating 0.1% polyethylene glycol 400 (PEG400), exhibiting excellent physicochemical properties. The hydrogel rapidly gelled at 34 °C, and rheological analysis showed a significant increase in storage modulus (G′) with rising temperature, indicating favorable thermosensitivity and mechanical stability (**Figure** **7**a,b,d–f). Scanning electron microscopy revealed a uniform porous structure in the lyophilized hydrogel, facilitating exosome encapsulation and sustained release (Figure 7c). FTIR analysis revealed an enhanced and broadened O–H/N–H stretching band (≈3350 cm⁻¹), suggesting strengthening of the hydrogen‐bond network during gelation. A shift or attenuation of the N–H peak (≈1560 cm⁻¹) may be attributed to the protonation of amino groups (–NH₂) to –NH₃⁺, forming ionic interactions with phosphate groups (–PO₄^3^⁻). The overlap or shift of P–O (≈1080 cm⁻¹) and C–O (≈1070 cm⁻¹) peaks further indicated potential physical or chemical interactions between chitosan and β‐GP (Figure 7h), supporting the formation of a stable 3D hydrogel network.

To assess the suitability of the hydrogel for exosome delivery, we further compared its physicochemical properties before and after loading G12G13MExos. The results showed no significant differences in gelation temperature, porous morphology, mechanical properties (G′), or FTIR spectral features following exosome incorporation, suggesting that the hydrogel retained its thermosensitive behavior and structural stability upon drug loading (Figure 7b–f,h). Elemental mapping revealed uniform distribution of chemical elements throughout the hydrogel matrix (Figure 7g), confirming the homogeneity of the formulation. Confocal microscopy further demonstrated that DiO‐labeled exosomes were evenly distributed within the hydrogel without visible aggregation (Figure 7i), supporting the matrix's encapsulation efficiency and compatibility. Moreover, the incorporation of PEG400 significantly reduced the surface tension and viscosity of the precursor solution, thereby improving atomization efficiency and spray uniformity (Figure 7j–l). Release kinetics analysis was performed under simulated nasal mucosal conditions (pH 6.0, 34 °C),^[^ ^40^, ^41^, ^42^ ^]^ revealing a sustained release profile with cumulative exosome release exceeding 70% over 10 days (Figure 7m).

To evaluate the biodegradability of the hydrogel, both in vitro and in vivo degradation studies were conducted. For in vitro assessment, hydrogel samples were incubated in PBS containing 1 mg mL^−1^ lysozyme (pH 6.0, 34 °C),^[^ ^40^, ^41^, ^42^ ^]^ simulating the enzymatic environment of the nasal cavity. Complete degradation was observed within ≈7 days (Figure S24a, Supporting Information). For in vivo analysis, two methods were applied: first, the residual hydrogel in the nasal cavity was collected and weighed after freeze‐drying at predetermined time points, revealing nearly complete degradation by day 4 (Figure S24b, Supporting Information); second, nasal lavage was performed and the fluorescence signal intensity was monitored, showing consistent degradation kinetics (Figure S24c,d, Extended Data Video materials S1, Supporting Information). It is worth noting that the apparent discrepancy between in vitro and in vivo degradation times may be partially attributed to experimental limitations, such as incomplete recovery of residual gel during nasal dissection or suboptimal flushing efficiency during lavage, due to the narrow anatomical space of the mouse nasal cavity. Despite these differences, both sets of results consistently demonstrate that the PEG400‐modified CS/β‐GP hydrogel possesses favorable and complete biodegradability under physiological conditions.

Collectively, these results demonstrate that the PEG400‐modified CS/β‐GP thermosensitive hydrogel exhibits rapid gelation, stable mechanical properties, excellent biodegradability, and sustained‐release capability, providing a robust and biocompatible platform for intranasal exosome delivery.

**Figure 8:**
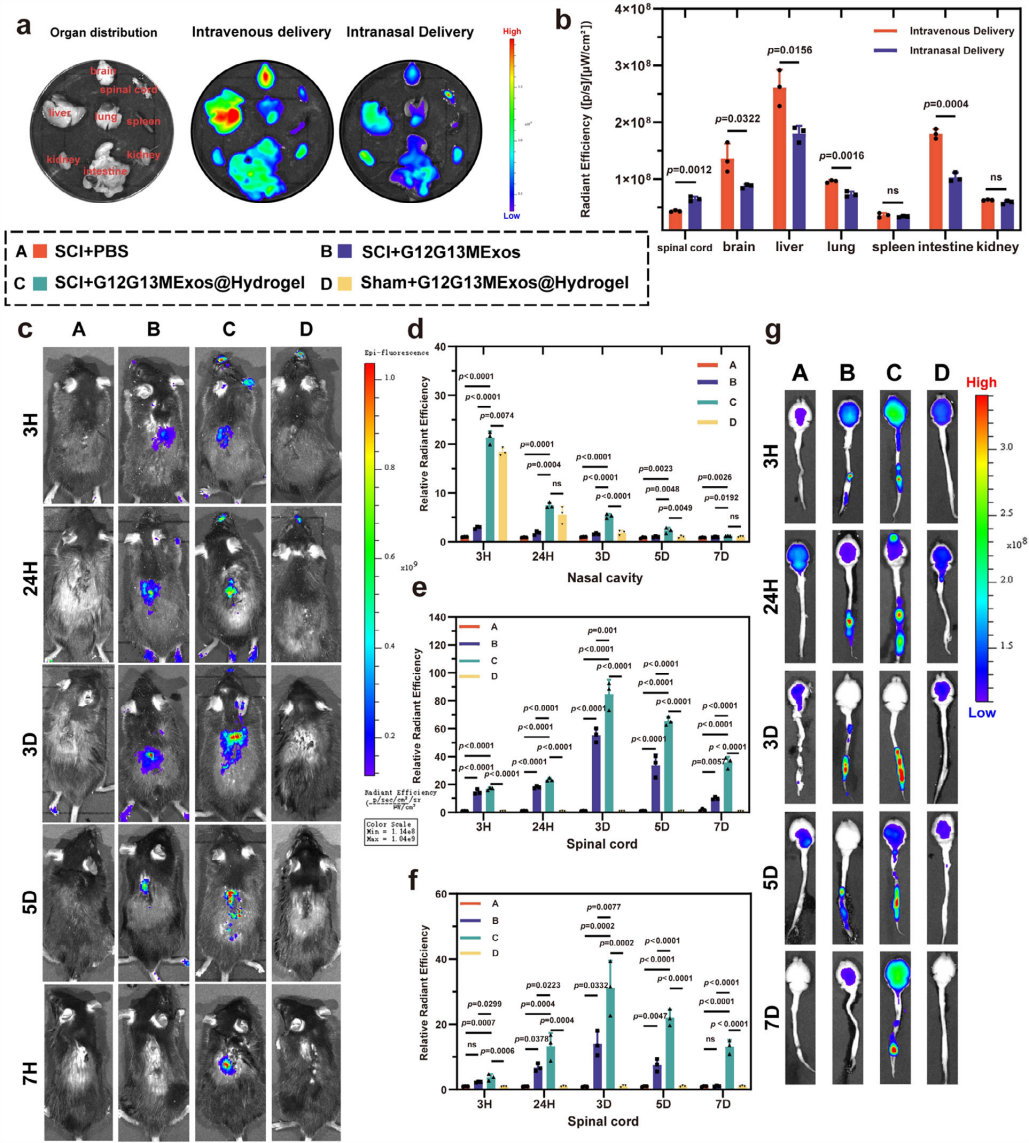
In vivo biodistribution and spinal targeting efficiency of G12G13MExos delivered via intravenous and intranasal hydrogel systems. a) Ex vivo fluorescence imaging of major organs (spinal cord, brain, liver, lung, spleen, intestine, kidney) on day 7 after three consecutive daily doses of G12G13MExos delivered via intravenous or intranasal routes. The intranasal group showed preferential spinal cord accumulation. b) Quantification of radiant efficiency in each organ. Intranasal delivery significantly enhanced accumulation in the spinal cord and brain while reducing off‐target liver and kidney signals (n = 3). c) In vivo fluorescence imaging of mice at 3 h, 1, 3, 5, and 7 days post‐administration. Groups included: SCI mice treated with PBS (A), G12G13MExos solution (B), or G12G13MExos@Hydrogel (C), and Sham‐operated mice receiving G12G13MExos@Hydrogel (D). The G12G13MExos@Hydrogel group showed the strongest and most sustained spinal fluorescence signal, while the Sham group exhibited negligible spinal accumulation, indicating injury‐dependent targeting. d,e) Quantitative analysis of relative radiant efficiency in the nasal cavity (d) and spinal cord (e) at the indicated time points. G12G13MExos@Hydrogel enabled rapid transport and prolonged retention in both regions (n = 3). f) Ex vivo imaging of isolated spinal cords at matched time points, supporting sustained accumulation of exosomes when encapsulated in hydrogel. g) Quantification of radiant efficiency in ex vivo spinal cords confirms significantly enhanced retention in the G12G13MExos@Hydrogel group up to 7 days post‐administration (n = 3). Data are presented as mean ± SD. Statistical analysis was performed using one‐way ANOVA followed by Tukey's multiple comparisons test. Exact *p*‐values are shown in the Figure ns, not significant.

Longitudinal imaging revealed that G12G13MExos@Hydrogel exhibited significantly stronger and more sustained fluorescence in the spinal cord region, with detectable signal from 3 h through day 7 post‐administration (*p* < 0.0001) (**Figure** **8**c,e), indicating enhanced targeting efficiency and prolonged retention in vivo. Meanwhile, analysis of nasal fluorescence (Figure 8b) revealed strong early signals in hydrogel‐treated groups (*p* < 0.0001), which gradually declined by day 5, confirming mucosal retention and controlled exosome release.

To eliminate potential artifacts from hair or surgical incisions, ex vivo fluorescence imaging of isolated spinal cords was additionally performed (Figure 8f,g). These results mirrored in vivo findings, with the G12G13MExos@Hydrogel group displaying the most prominent and durable spinal fluorescence. Furthermore, uninjured control mice receiving the same treatment exhibited negligible spinal signal, reinforcing that exosome accumulation is driven by injury‐site pathology rather than passive systemic circulation.

To further verify cellular‐level uptake and targeting specificity, we performed immunofluorescence staining of spinal cord tissues to assess co‐localization of DiD‐labeled G12G13MExos with specific cell types. Strong exosome uptake signals were observed in Iba1⁺ microglia and GFAP⁺ astrocytes, particularly around the lesion core, while NeuN⁺ neurons exhibited minimal colocalization (Figure S25, Supporting Information). These findings indicate that glial cells are the primary recipients of G12G13MExos in vivo and support our proposed mechanism whereby exosome‐mediated regulation of glial phenotypes indirectly facilitates neuronal repair through glia–neuron crosstalk.

Spinal cord immunofluorescence further confirmed rapid, targeted transport in the G12G13MExos@Hydrogel group, with exosome quantity increasing significantly over time and showing improved targeting and utilization (*p* < 0.001) (Figure S26, Supporting Information).

Compared to traditional injection methods, the “nasal exosome intelligent sustained‐release reservoir” significantly enhances delivery efficiency and addresses the limitations of IV and local injection. IV injection suffers from low bioavailability due to hepatic and renal clearance and BSCB obstruction, while plain intranasal delivery, although bypassing the BSCB, is hindered by limited absorption efficiency and rapid exosome clearance. The CS/β‐GP hydrogel, with its rapid gelation and sustained‐release properties, forms a thin film on the nasal mucosa, enabling continuous exosome release and substantially improving targeted delivery efficiency and stability.

To further elucidate the delivery mechanism, we collected cerebrospinal fluid (CSF) from mice after intranasal administration of DiD‐labeled exosomes. Fluorescence signal analysis revealed significantly elevated DiD intensity in the CSF of treated animals compared to PBS controls, supporting the hypothesis that exosomes cross the nasal epithelium and enter the CSF compartment (Figure S27, Supporting Information). This finding confirms that the nasal epithelium–CSF–spinal cord route serves as a key pathway by which exosomes reach the injury site,^[^ ^43^, ^44^, ^45^, ^46^ ^]^ enhancing the biological plausibility of our nasal delivery system.

In vitro cytocompatibility assays were first conducted to evaluate the biocompatibility of the CS/β‐GP hydrogel. Live/dead staining and CCK‐8 proliferation assays using A549 epithelial cells demonstrated that the hydrogel maintained good cell viability and proliferation at concentrations up to 5 mg mL^−1^. A slight reduction in cell activity was observed only at 10 mg mL^−1^, a concentration substantially higher than that used in vivo (Figure S28, Supporting Information). These results confirmed that the hydrogel exhibited favorable cytocompatibility and was well‐suited for nasal mucosal applications.

To further verify its biosafety in vivo, systematic evaluations were performed in SCI mouse models. Blood chemistry analysis showed no significant differences in ALT, AST, Cr, or UREA levels after 14 days of nasal hydrogel application (Figure S29a, Supporting Information). Histological examination of nasal tissue via HE staining revealed no morphological abnormalities (Figure S29b, Supporting Information), and HE staining of major organs (heart, lung, liver, kidney, spleen) at 28 days post‐administration showed no signs of inflammation or structural damage (Figure S29c, Supporting Information). Together, these findings demonstrate the excellent biocompatibility and tissue safety of the CS/β‐GP hydrogel both in vitro and in vivo.

In summary, the “nasal exosome intelligent sustained‐release reservoir” not only significantly improves exosome delivery efficiency and targeting but also demonstrates excellent safety. Its thermosensitive gelation and sustained‐release capabilities provide a reliable foundation for efficient exosome delivery, offering strong support for the clinical translation of exosome therapy in SCI repair.

### 2.5 Enhanced Functional Recovery with GNA12/GNA13‐Mediated Exosome Therapy

**Figure 9:**
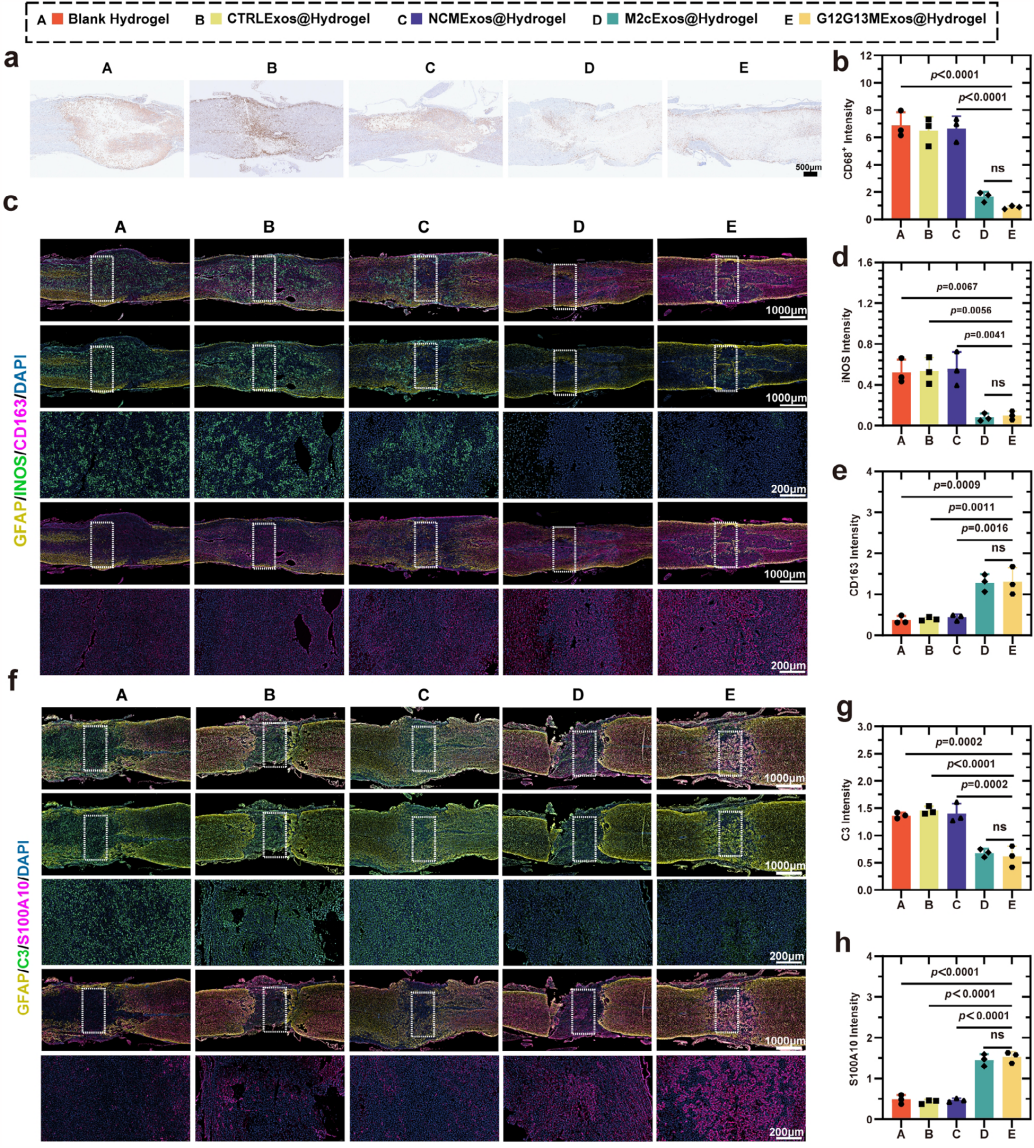
G12G13MExos@Hydrogel modulates glial activation and reshapes the neuroinflammatory microenvironment after SCI. a) Representative immunohistochemical staining of CD68⁺ macrophages in spinal cord sections from Blank Hydrogel (A), CTRLExos@Hydrogel (B), NCMExos@Hydrogel (C), M2cExos@Hydrogel (D), and G12G13MExos@Hydrogel (E) groups. Scale bar: 500 µm. b) Quantification of CD68⁺ signal intensity. G12G13MExos@Hydrogel treatment significantly reduced macrophage infiltration (n = 3). c–e) Immunofluorescence staining of spinal cord sections showing glial co‐labeling with GFAP (yellow), iNOS (green, M1 microglia marker), CD163 (magenta, M2c microglia marker), and DAPI (blue). G12G13MExos@Hydrogel treatment suppressed iNOS and enhanced CD163 expression. Scale bars: 1000 µm (overview), 200 µm (zoom‐in). d,e) Quantification of iNOS⁺ and CD163⁺ signal intensity confirms M1‐to‐M2c phenotypic shift in the G12G13MExos@Hydrogel group (n = 3). f–h) Immunofluorescence staining of astrocytes showing GFAP (yellow), C3 (green, A1 marker), S100A10 (magenta, A2 marker), and DAPI (blue). G12G13MExos@Hydrogel treatment decreased neurotoxic C3⁺ astrocytes and increased S100A10⁺ astrocytes. Scale bars: 1000 µm (overview), 200 µm (zoom‐in). g,h) Quantification of C3⁺ and S100A10⁺ fluorescence intensity, demonstrating that G12G13MExos@Hydrogel shifted astrocyte phenotypes from A1 to A2 (n = 3). Data are presented as mean ± SD. Statistical analysis was performed using one‐way ANOVA followed by Tukey's multiple comparisons test. Exact *p*‐values are shown in the Figure ns, not significant.

In the SCI model established in C57BL/6 mice, hydrogels loaded with G12G13M exosomes (G12G13MExos@Hydrogel) significantly improved the inflammatory microenvironment at the injury site. Immunohistochemistry revealed a marked reduction in infiltrating hematogenous macrophages by day 7 post‐injury (*p* < 0.0001), alleviating the prolonged inflammation caused by M1 macrophage dominance (**Figure** **9**a,b).^[^ ^47^ ^]^ This effect may be attributed to the exosomes’ ability to regulate the expression of chemokines, such as CCL2 and CXCL10, thereby reducing macrophage migration to the injury site.^[^ ^48^ ^]^ Immunofluorescence further demonstrated that, compared to the NCMExos@Hydrogel group, G12G13MExos@Hydrogel significantly inhibited the polarization of microglia into the M1 phenotype (*p* = 0.0041) while enhancing their transition to the anti‐inflammatory M2 phenotype (*p* < 0.0016) (Figure 9c–e). This effect may involve the synergistic action of TGF‐β and IL‐10 carried by the exosomes, which activate the Smad signaling pathway. ELISA results also showed significant upregulation of anti‐inflammatory cytokines IL‐10 and TGF‐β by day 3 post‐injury (*p* = 0.0298 and *p* = 0.0104, respectively), with sustained increases over time (Figure S30a,b, Supporting Information). These dynamic changes indicate that G12G13MExos contributes to SCI tissue repair and functional recovery by reducing hematogenous macrophage infiltration, regulating microglial phenotypes, and maintaining elevated levels of anti‐inflammatory cytokines.

To examine astrocyte distribution and gene expression changes in the SCI lesion, we analyzed a single‐cell RNA sequencing dataset from Li et al.,^[^ ^4^ ^]^ enabling systematic characterization of astrocyte subtype dynamics across different injury stages (Figure S31, Supporting Information). The results showed a significant time‐dependent increase in the expression of C3, a marker of neurotoxic astrocytes, indicating their progressive dominance in the inflammatory microenvironment (Figure S31b, Supporting Information). Conversely, the expression of S100A10, associated with neuroprotective astrocytes, slightly increased at early stages (day 3) but rapidly declined thereafter (Figure S31c, Supporting Information). Immunofluorescence and qPCR analyses confirmed these trends, with neurotoxic astrocytes progressively increasing and neuroprotective astrocytes markedly decreasing (Figure S32, Supporting Information). This shift may be linked to hypoxia‐ischemia stress and pro‐inflammatory cytokines (e.g., TNF‐α) within the inflammatory microenvironment post‐SCI.^[^ ^49^ ^]^ These findings underscore the critical importance of modulating reactive astrocytes to transition into neuroprotective phenotypes during the first week post‐SCI.^[^ ^50^ ^]^ G12G13MExos@Hydrogel significantly inhibited the activation of neurotoxic astrocytes (*p* = 0.0002) and enhanced the proportion of neuroprotective astrocytes by day 7 post‐SCI (*p* < 0.0001) (Figure 9f–h). Consistent with in vitro findings, these results confirm that G12G13MExos@Hydrogel ameliorates the inflammatory microenvironment and facilitates neurorepair. Compared to conventional approaches, G12G13MExos@Hydrogel offers distinct advantages in modulating astrocyte phenotypes, establishing a solid foundation for the development of astrocyte‐targeted therapies. This study not only delineates the dynamic pathological evolution of astrocytes following SCI, but also highlights the therapeutic potential and translational value of G12G13MExos@Hydrogel in SCI treatment.

**Figure 10:**
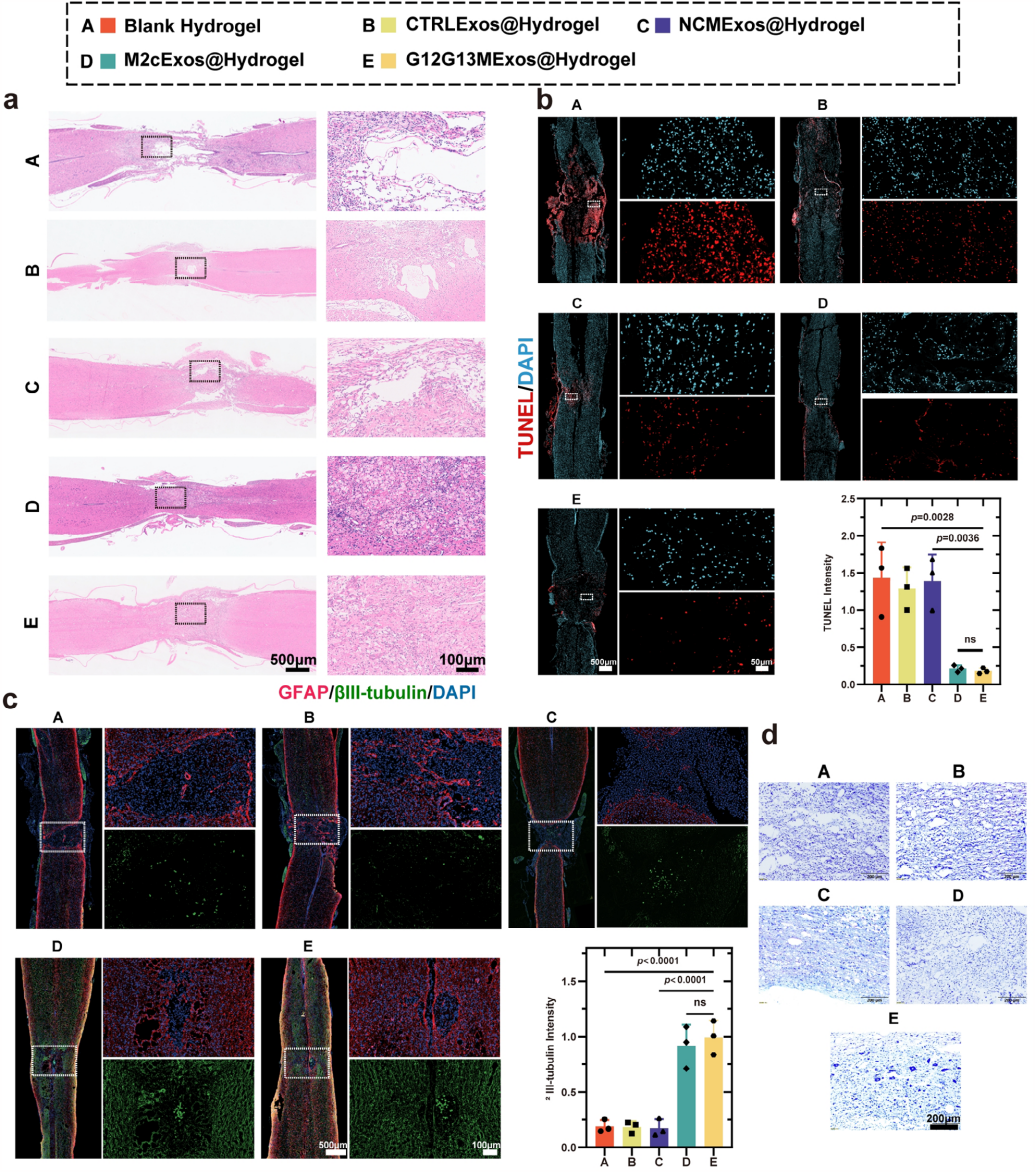
G12G13MExos@Hydrogel promotes neuronal survival, axonal regeneration, and functional recovery after SCI. a) Representative H&E staining of spinal cord tissue at 28 days post‐SCI across five groups: Blank Hydrogel (A), CTRLExos@Hydrogel (B), NCMExos@Hydrogel (C), M2cExos@Hydrogel (D), and G12G13MExos@Hydrogel (E). G12G13MExos@Hydrogel group showed preserved spinal cord continuity and reduced cavitation (n = 3). Scale bars: 500 µm (left), 100 µm (right). b) Representative TUNEL staining of spinal cord sections on day 3 post‐injury, showing apoptotic cells (red) and nuclei (blue). G12G13MExos@Hydrogel markedly reduced apoptosis (n = 3). Scale bar: 100 µm. c) Immunofluorescence staining of βIII‐tubulin (green, neuronal marker) and GFAP (red, astrocyte marker) in spinal cord sections at day 28 post‐injury. G12G13MExos@Hydrogel group exhibited the highest neuronal survival and axonal integrity (n = 3). Scale bars: 500 µm (left), 100 µm (right). d) Nissl staining at day 7 post‐SCI showing neuronal preservation. The G12G13MExos@Hydrogel group exhibited clearer, denser Nissl bodies compared to other treatments (n = 3). Scale bar: 200 µm. Data are presented as mean ± SD. Statistical analysis was performed using one‐way ANOVA followed by Tukey's multiple comparisons test. Exact *p*‐values are shown in the Figure ns, not significant.

Macroscopically, G12G13MExos@Hydrogel markedly alleviated swelling in the SCI lesion area. The tissue surface appeared smoother, and its coloration more closely resembled healthy tissue, suggesting reduced inflammation and structural damage (Figure S33a, Supporting Information). HE staining further confirmed that by day 28 post‐SCI, the lesion cavity area was significantly reduced in the G12G13MExos@Hydrogel group, with restored spinal cord continuity, decreased inflammatory cell infiltration, and minimal glial fibrosis (**Figure** **10**a). These results indicate that G12G13MExos@Hydrogel effectively promotes tissue repair and regeneration following SCI. Functional assessments also demonstrated notable improvements. Basso Mouse Scale (BMS) scores revealed significant restoration of motor function (Figure S33b, Supporting Information), accompanied by progressive increases in body weight (Figure S33c, Supporting Information). Open‐field footprint analysis showed improved gait coordination (Figure S33d, Supporting Information), and swim test results indicated enhanced Louisville Swim Scale scores, further supporting motor recovery (Figure S34; Extended Data Videos S2–S6, Supporting Information).

Furthermore, G12G13MExos@Hydrogel alleviated SCI‐induced bladder dysfunction. HE staining of bladder tissues revealed increased bladder wall thickness (*p* < 0.001) and more organized structural integrity (Figure S35, Supporting Information), indicating significant improvement in bladder function.

Collectively, these findings underscore the multidimensional therapeutic efficacy of G12G13MExos@Hydrogel in promoting structural, motor, and autonomic recovery after SCI. In parallel, G12G13MExos@Hydrogel exhibited significant neuroprotective effects. TUNEL staining revealed a marked reduction in apoptotic cells by day 3 post‐SCI in the G12G13MExos@Hydrogel group (*p* = 0.0036), indicating effective early suppression of neuronal apoptosis (Figure 10b).

By day 7, Nissl staining demonstrated more uniform neuronal morphology, higher neuronal density, and improved structural integrity in this group, further validating its neuroprotective potential (Figure 10d). On day 28, βIII‐tubulin immunofluorescence staining revealed the strongest signal intensity in the G12G13MExos@Hydrogel group (*p* < 0.0001), indicating enhanced neuronal regeneration (Figure 10c). Additionally, spinal cord immunofluorescence showed significantly increased VSX2 expression (*p* < 0.0001), suggesting effective promotion of neural stem cell differentiation into VSX2⁺ interneurons (Figure 6i,j).

Transcriptomic analysis of spinal cord tissue at day 28 post‐SCI revealed substantial changes in gene expression related to inflammation regulation, neuroprotection, neurotrophy, and neural stem cell differentiation in the G12G13MExos@Hydrogel group. Heatmap analyses showed marked downregulation of pro‐inflammatory genes (e.g., *Tnf, Il1b, Ccl2*) and upregulation of anti‐inflammatory genes (e.g., *Tgfb1, Il10, Pparg*), underscoring the hydrogel's potent anti‐inflammatory effects (Figure S36a, Supporting Information). In addition, key neuroprotective and neurotrophic genes (e.g., *Bdnf, Ngf, Gdnf, Map2*) were significantly upregulated, supporting enhanced neuronal survival and repair (Figure S36b, Supporting Information). Markers of neural stem cell differentiation (e.g., *Sox2, Pax6, Dcx*) were also significantly elevated, highlighting the system's regenerative potential (Figure S36c, Supporting Information). Together, these findings indicate that G12G13MExos@Hydrogel promotes functional recovery post‐SCI through coordinated modulation of inflammation, neuroprotection, and stem cell–mediated regeneration.

To further validate the regulatory mechanisms underlying the neuroprotective and reparative effects of G12G13MExos, we investigated the activation status of two key signaling pathways—PI3K‐AKT and NF‐κB—at the tissue level. Spinal cord tissues were harvested on day 7 post‐SCI, a time point marking the transition from acute inflammation to the early repair phase, providing an optimal window for detecting pathway modulation. Western blot analysis revealed that the G12G13MExos@Hydrogel group exhibited significantly elevated levels of phosphorylated PI3K and AKT1 compared to the control groups (Figure S37a–c, Supporting Information), indicating activation of the PI3K‐AKT pathway. Consistently, immunofluorescence imaging showed enhanced p‐AKT signals in the lesion area (Figure S37d,e, Supporting Information), suggesting activation of downstream signaling related to cell survival and neuroregeneration. Conversely, the expression of phosphorylated p65 and IκBα—key indicators of NF‐κB activation—was significantly reduced in the G12G13MExos@Hydrogel group (Figure S38a–c, Supporting Information). This suppression was further supported by immunofluorescence analysis, which demonstrated markedly diminished p‐p65 expression in the injured spinal cord (Figure S38d,e, Supporting Information). Together, these results confirm that the G12G13MExos‐based delivery system effectively inhibits NF‐κB–mediated pro‐inflammatory signaling while concurrently activating PI3K‐AKT–driven reparative pathways.

These in vivo findings provide both protein‐level and spatial validation of the transcriptomic trends observed on day 28, demonstrating that G12G13MExos@Hydrogel promotes functional recovery through coordinated modulation of inflammation resolution and neuroregenerative signaling in the injured spinal cord microenvironment.

## 3 Conclusion

### 3.1 Translating Engineered Exosomes into Multi‐Targeted SCI Therapies

This study systematically elucidates the critical roles of macrophage‐derived exosomes overexpressing GNA12 and GNA13 (G12G13MExos) in modulating astrocyte phenotype transition, improving the lesion microenvironment and promoting neuroprotection in SCI. Furthermore, it introduces an innovative “nasal exosome intelligent sustained‐release depot,” offering a comprehensive multi‐target strategy for SCI treatment. The overexpression of GNA12 and GNA13 significantly promotes macrophage polarization into the M2c phenotype, endowing exosomes with anti‐inflammatory properties and inflammatory targeting capabilities, which exhibit notable advantages in suppressing inflammation and modulating the neural microenvironment.

G12G13MExos exerts its effects through the activation of the PI3K‐AKT pathway and inhibition of the NF‐κB pathway, facilitating myelin debris clearance, glutamate homeostasis, synapse formation, and the differentiation of neural stem cells into V2a neurons. These mechanisms provide a novel basis for neural circuit reconstruction. In vivo experiments confirmed the efficacy of G12G13MExos in mitigating neuronal apoptosis in the early phase of SCI and enhancing neuroregeneration in the intermediate phase, leading to significant improvements in motor and bladder functions in mice and demonstrating superior overall repair capacity.

This study also introduces a groundbreaking nasal delivery strategy. Compared to traditional intravenous injection, nasal administration bypasses hepatic and renal clearance, as well as the BSCB, significantly enhancing the targeting efficiency and bioavailability of exosomes. A thermosensitive chitosan hydrogel achieves sustained targeted delivery of exosomes through a controlled‐release mechanism, further amplifying their therapeutic potential. Transcriptomic sequencing and histological analyses validated the system's significant advantages in regulating inflammation, enhancing neuroprotection, and promoting neuroregeneration.

The integration of GNA12/GNA13‐engineered exosomes with an intelligent material‐based delivery system presents a multi‐target, non‐invasive therapeutic strategy for SCI, demonstrating promising potential for clinical translation. This research not only provides new insights into SCI mechanisms and treatment but also lays a solid foundation for expanding exosome applications and optimizing delivery systems.

## Conflict of Interest

The authors declare no conflict of interest.

## Supporting information



Supporting Information

Supplemental Video 1

Supplemental Video 2

Supplemental Video 3

Supplemental Video 4

Supplemental Video 5

## Data Availability

The data that support the findings of this study are available from the corresponding author upon reasonable request.

## References

[1] J. H. Badhiwala , C. S. Ahuja , M. G. Fehlings , J. Neurosurg. Spine 2019, 30, 1. 10.3171/2018.9.SPINE18682 30611186

[2] C. S. Ahuja , J. R. Wilson , S. Nori , M. R. N. Kotter , C. Druschel , A. Curt , M. G. Fehlings , Nat. Rev. Dis. Primers 2017, 3, 17018. 28447605 10.1038/nrdp.2017.18

[3] J. W. Jeong , C. Y. Jin , G. Y. Kim , J. D. Lee , C. Park , G. D. Kim , W.‐J. Kim , W.‐K. Jung , S. K. Seo , I.‐W. Choi , Y. H. Choi , Int. Immunopharmacol. 2010, 10, 1580. 20937401 10.1016/j.intimp.2010.09.011

[4] X. Hu , W. Xu , Y. Ren , Z. Wang , X. He , R. Huang , B. Ma , J. Zhao , R. Zhu , L. Cheng , Signal Transduction Targeted Ther. 2023, 8, 245. 10.1038/s41392-023-01477-6 PMC10291001 37357239

[5] A. Verkhratsky , M. Nedergaard , Physiol. Rev. 98, 239. 29351512 10.1152/physrev.00042.2016 PMC6050349

[6] R. M. Ransohoff , Science 2016, 353, 777. 27540165 10.1126/science.aag2590

[7] K. L. Spiller , R. R. Anfang , K. J. Spiller , J. Ng , K. R. Nakazawa , J. W. Daulton , G. Vunjak‐Novakovic , Biomaterials 2014, 35, 4477. 24589361 10.1016/j.biomaterials.2014.02.012 PMC4000280

[8] S. J. Lee , J. W. Yang , I. J. Cho , W. D. Kim , M. K. Cho , C. H. Lee , S. G. Kim , Oncogene 2009, 28, 1230. 19151758 10.1038/onc.2008.488

[9] M. M. Miyake , B. S. Bleier , Am. J. Rhinol. Allergy 2015, 29, 124. 25785753 10.2500/ajra.2015.29.4149

[10] D. Li , N. Wu , Diabetes Res. Clin. Pract. 2022, 187, 109882. 35487341 10.1016/j.diabres.2022.109882

[11] G. Séjourné , C. Eroglu , Curr. Opin. Neurobiol. 2024, 89, 102925. 39357429 10.1016/j.conb.2024.102925 PMC12186082

[12] Y. Zhang , Z. Wang , F. Xu , Z. Liu , Y. Zhao , L. Z. Yang , W. Fang , Neurochem. Res. 2024, 49, 3187. 39292330 10.1007/s11064-024-04241-6

[13] W. Li , D. Wei , J. Lin , J. Liang , X. Xie , K. Song , L.’a. Huang , Front. Cell Neurosci. 2019, 13, 351. 31456664 10.3389/fncel.2019.00351 PMC6701226

[14] P. Yue , L. Gao , X. Wang , X. Ding , J. Teng , Neurochem. Res. 2017, 42, 1366. 28247332 10.1007/s11064-017-2184-1

[15] J. W. McDonald , C. Sadowsky , Lancet 2002, 359, 417. 11844532 10.1016/S0140-6736(02)07603-1

[16] S. Duan , C. M. Anderson , B. A. Stein , R. A. Swanson , J. Neurosci. 1999, 19, 10193. 10575016 10.1523/JNEUROSCI.19-23-10193.1999 PMC6782431

[17] R. Sitcheran , P. Gupta , P. B. Fisher , A. S. Baldwin , EMBO J. 2005, 24, 510. 15660126 10.1038/sj.emboj.7600555 PMC548660

[18] E. Pajarillo , A. Rizor , J. Lee , M. Aschner , E. Lee , Neuropharmacology 2019, 161, 107559. 30851309 10.1016/j.neuropharm.2019.03.002 PMC6731169

[19] M. T. Filbin , Nat. Rev. Neurosci. 2003, 4, 703. 12951563 10.1038/nrn1195

[20] H. Konishi , T. Okamoto , Y. Hara , O. Komine , H. Tamada , M. Maeda , F. Osako , M. Kobayashi , A. Nishiyama , Y. Kataoka , T. Takai , N. Udagawa , K. Ozato , T. Tamura , M. Tsuda , K. Yamanaka , T. Ogi , K. Sato , H. Kiyamaet , EMBO J. 2020, 39, 104464. 10.15252/embj.2020104464 PMC7667883 32959911

[21] W. S. Chung , L. E. Clarke , G. X. Wang , B. K. Stafford , A. Sher , C. Chakraborty , J. Joung , L. C. Foo , A. Thompson , C. Chen , S. J. Smith , B. A. Barres , Nature 2013, 504, 394. 24270812 10.1038/nature12776 PMC3969024

[22] Y. M. Morizawa , Y. Hirayama , N. Ohno , S. Shibata , E. Shigetomi , Y. Sui , J. Nabekura , K. Sato , F. Okajima , H. Takebayashi , H. Okano , S. Koizumi , Nat. Commun. 2017, 8, 28. 28642575 10.1038/s41467-017-00037-1 PMC5481424

[23] T. Xu , C. Liu , S. Deng , L. Gan , Z. Zhang , G. Y. Yang , H. Tian , Y. Tang , J. Cereb. Blood Flow Metab. 2023, 43, 325. 36324281 10.1177/0271678X221137762 PMC9941857

[24] A. Semyanov , A. Verkhratsky , Trends Neurosci. 2021, 44, 781. 34479758 10.1016/j.tins.2021.07.006

[25] M. Arizono , U. V. Nägerl , Glia 2022, 70, 607. 34664734 10.1002/glia.24103

[26] N. J. Allen , M. L. Bennett , L. C. Foo , G. X. Wang , C. Chakraborty , S. J. Smith , B. A. Barres , Nature 2012, 486, 410. 22722203 10.1038/nature11059 PMC3383085

[27] N. J. Allen , D. A. Lyons , Science 2018, 362, 181. 30309945 10.1126/science.aat0473 PMC6292669

[28] M. R. Patel , A. M. Weaver , Cell Rep. 2021, 34, 108829. 33691102 10.1016/j.celrep.2021.108829 PMC8002899

[29] C. Z. Tang , J. T. Yang , Q. H. Liu , Y. R. Wang , W. S. Wang , FASEB J. 2019, 33, 606. 30118321 10.1096/fj.201800210RR

[30] Y. Li , X. He , R. Kawaguchi , Y. Zhang , Q. Wang , A. Monavarfeshani , Z. Yang , B. Chen , Z. Shi , H. Meng , S. Zhou , J. Zhu , A. Jacobi , V. Swarup , P. G. Popovich , D. H. Geschwind , Z. He , Nature 2020, 587, 613. 33029008 10.1038/s41586-020-2795-6 PMC7704837

[31] L. D. Hachem , A. J. Mothe , C. H. Tator , Stem Cells 2020, 38, 187. 31648407 10.1002/stem.3107

[32] F. Barnabé‐Heider , C. Göritz , H. Sabelström , H. Takebayashi , F. W. Pfrieger , K. Meletis , J. Frisén , Cell Stem Cell 2010, 7, 470. 20887953 10.1016/j.stem.2010.07.014

[33] X. Li , D. Liu , Z. Xiao , Y. Zhao , S. Han , B. Chen , J. Dai , Biomaterials 2019, 197, 20. 30639547 10.1016/j.biomaterials.2019.01.012

[34] J. W. Squair , M. Milano , A. de Coucy , M. Gautier , M. A. Skinnider , N. D. James , N. Cho , A. Lasne , C. Kathe , T. H. Hutson , S. Ceto , L. Baud , K. Galan , V. Aureli , A. Laskaratos , Q. Barraud , T. J. Deming , R. E. Kohman , B. L. Schneider , Z. He , J. Bloch , M. V. Sofroniew , G. Courtine , M. A. Anderson , Science 2023, 381, 1338. 37733871 10.1126/science.adi6412

[35] G. Courtine , B. Song , R. R. Roy , H. Zhong , J. E. Herrmann , Y. Ao , J. Qi , V. Reggie Edgerton , M. V. Sofroniew , Nat. Med. 2008, 14, 69. 18157143 10.1038/nm1682 PMC2916740

[36] M. A. Anderson , T. M. O'Shea , J. E. Burda , Y. Ao , S. L. Barlatey , A. M. Bernstein , J. H. Kim , N. D. James , A. Rogers , B. Kato , A. L. Wollenberg , R. Kawaguchi , G. Coppola , C. Wang , T. J. Deming , Z. He , G. Courtine , M. V. Sofroniew , Nature 2018, 561, 396. 30158698 10.1038/s41586-018-0467-6 PMC6151128

[37] W. Y. Li , L. X. Deng , F. G. Zhai , X. Y. Wang , Z. G. Li , Y. Wang , Neural Regener. Res. 2023, 18, 933. 10.4103/1673-5374.355746 PMC9827767 36254971

[38] A. Yamamoto , T. Iseki , M. Ochi‐Sugiyama , N. Okada , T. Fujita , S. Muranishi , J. Controlled Release 2001, 76, 363. 10.1016/s0168-3659(01)00454-0 11578749

[39] R. G. Thorne , G. J. Pronk , V. Padmanabhan , W. H. 2nd Frey ., Neuroscience 2004, 127, 481. 15262337 10.1016/j.neuroscience.2004.05.029

[40] Y. Liu , Y. Tan , G. Cheng , Y. Ni , A. Xie , X. Zhu , C. Yin , Y. Zhang , T. Chen , Adv. Mater. 2024, 36, 2307081. 10.1002/adma.202307081 38395039

[41] N. Maniyamgama , K. H. Bae , Z. W. Chang , J. Lee , M. J. Y. Ang , Y. J. Tan , L. F. P. Ng , L. Renia , K. P. White , Y. Y. Yang , Adv. Sci. (Weinh) 2025, 12, 2407383. 39888252 10.1002/advs.202407383 PMC11923898

[42] Y. Hong , H. Song , Y. Gong , Z. Mao , C. Gao , J. Shen , Acta Biomater. 2007, 3, 23. 16956800 10.1016/j.actbio.2006.06.007

[43] Z. Huang , J. Li , J. Wo , C. L. Li , Z. C. Wu , X. H. Deng , Y. Liang , F. Li , B. Chen , B. Jia , L. Wang , Y. Wang , G. Sun , Z. Li , H. Zhu , J. D. Guest , K.‐F. So , Q.‐L. Fu , L. Zhou , J. Extracell Vesicles 2025, 14, 70066. 10.1002/jev2.70066 PMC11975507 40194993

[44] S. Guo , N. Perets , O. Betzer , S. Ben‐Shaul , A. Sheinin , I. Michaelevski , R. Popovtzer , D. Offen , S. Levenberg , ACS Nano 2019, 13, 10015. 31454225 10.1021/acsnano.9b01892

[45] T. Ikeda , M. Kawabori , Y. Zheng , S. Yamaguchi , S. Gotoh , Y. Nakahara , E. Yoshie , M. Fujimura , Pharmaceutics 2024, 16, 446. 38675108 10.3390/pharmaceutics16040446 PMC11053690

[46] H. Shen , N. Aggarwal , B. Cui , G. W. Foo , Y. He , S. K. Srivastava , S. Li , M. Z. X. Seah , K. S. Wun , H. Ling , I. Y. Hwang , C. L. Ho , Y. S. Lee , M. W. Chang , Cell 2025, 188, 1545. 39914382 10.1016/j.cell.2025.01.017

[47] R. Shechter , O. Miller , G. Yovel , N. Rosenzweig , A. London , J. Ruckh , Ki‐W. Kim , E. Klein , V. Kalchenko , P. Bendel , S. A. Lira , S. Jung , M. Schwartz , Immunity 2013, 38, 555. 23477737 10.1016/j.immuni.2013.02.012 PMC4115271

[48] C. Gu , X. Geng , Y. Wu , Y. Dai , J. Zeng , Z. Wang , H. Fang , Y. Sun , X. Chen , Small 2024, 20, 2305659. 10.1002/smll.202305659 37884477

[49] J. L. Zamanian , L. Xu , L. C. Foo , N. Nouri , L. Zhou , R. G. Giffard , B. A. Barres , J. Neurosci. 2012, 32, 6391. 22553043 10.1523/JNEUROSCI.6221-11.2012 PMC3480225

[50] A. Kisucká , K. Bimbová , M. Bačová , J. Gálik , N. Lukáčová , Cells 2021, 10, 1943. 34440711 10.3390/cells10081943 PMC8394075

