# Supplementary material for: Nasal Delivery of Engineered Exosomes via a Thermo‐Sensitive Hydrogel Depot Reprograms Glial Cells for Spinal Cord Repair
Source: Adv Sci (Weinh). 2025 Jun 20;12(34):e04486. doi: 10.1002/advs.202504486 (PMC12442609; doi:10.1002/advs.202504486)
Supplement: Supplementary file 1 — Supporting Information [file ADVS-12-e04486-s002.docx]

**Supporting Information**

**Supporting Figure**


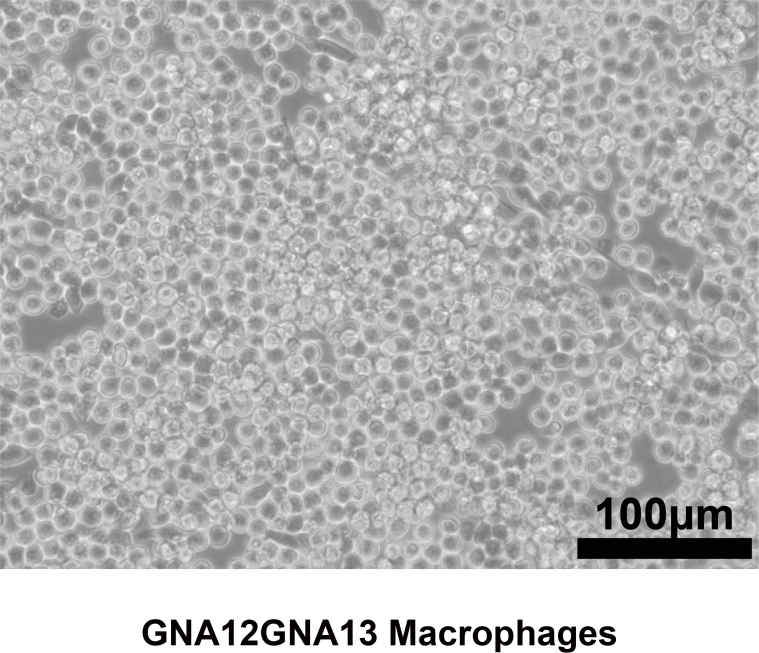


Supporting Figure 1. Morphology of GNA12GNA13-overexpressing macrophages.

Bright-field microscopy image of macrophages overexpressing GNA12 and GNA13, showing their characteristic morphology. Scale bar: 100 µm.


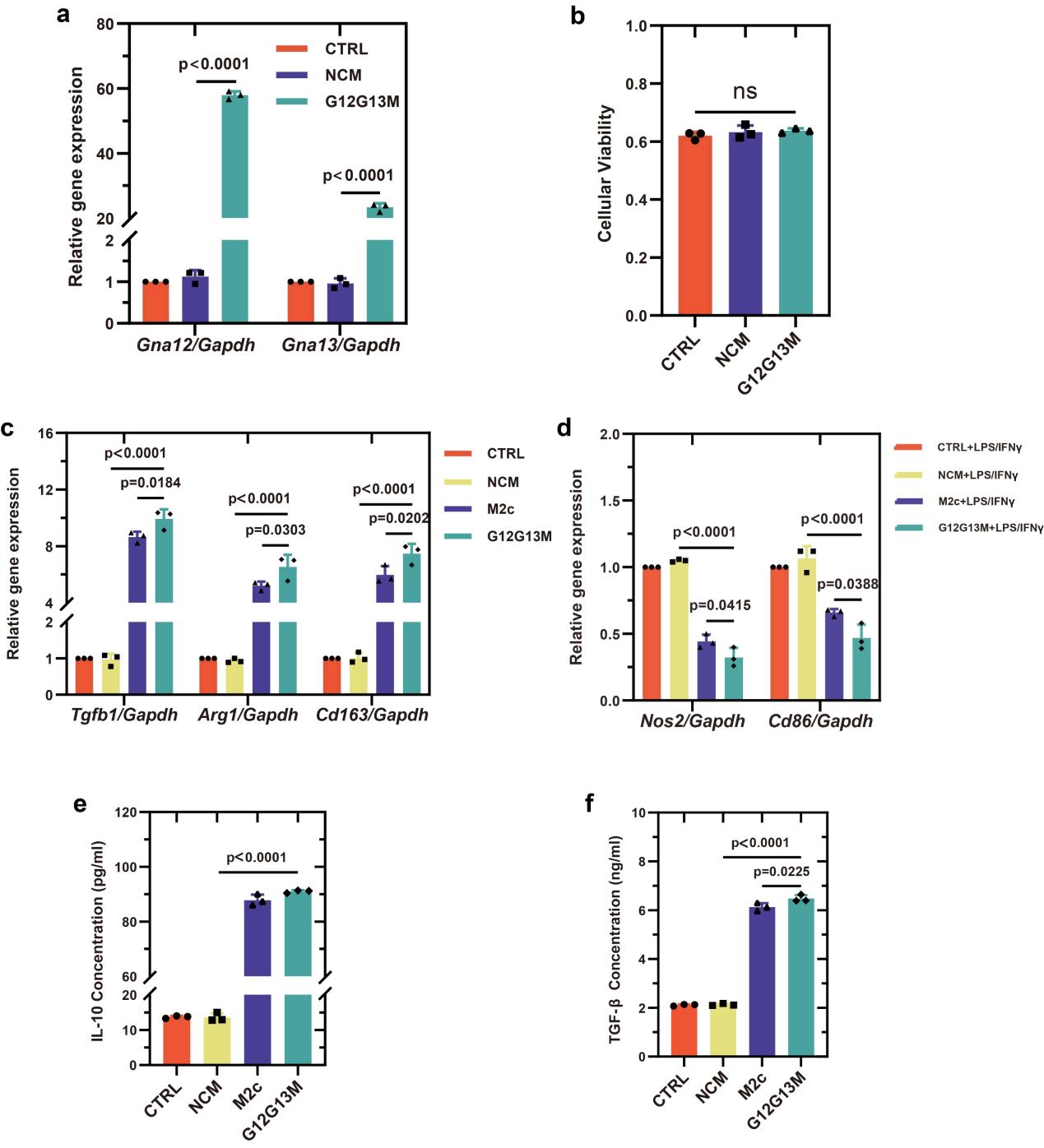


Supporting Figure 2. Characterization of GNA12/GNA13-overexpressing macrophages.

(a) Relative mRNA expression of *Gna12* and *Gna13* in control (CTRL), negative control macrophages (NCM), and GNA12/GNA13-overexpressing macrophages (G12G13M), confirming successful overexpression in the G12G13M group (n = 3).

(b) Cell viability of CTRL, NCM, and G12G13M macrophages, showing no significant differences (ns), indicating non-toxic effects of GNA12/GNA13 overexpression (n = 3).

(c) Expression of M2c-related genes (*Tgfb1, Arg1, Cd163*) under resting conditions. M2c and G12G13M macrophages exhibited significantly higher expression levels (n = 3).

(d) Expression of M1 markers (*Nos2, Cd86*) following LPS/IFN-γ stimulation. G12G13M macrophages showed suppressed M1 polarization compared to other groups (n = 3).

(e) IL-10 secretion measured by ELISA, showing elevated levels in M2c and G12G13M groups (n = 3).

(f) TGF-β secretion measured by ELISA, also increased in M2c and G12G13M macrophages (n = 3).

Data are presented as mean ± SD. Statistical analysis was performed using one-way ANOVA followed by Tukey’s multiple comparisons test. Exact *p-*values are shown in the figure. ns, not significant.


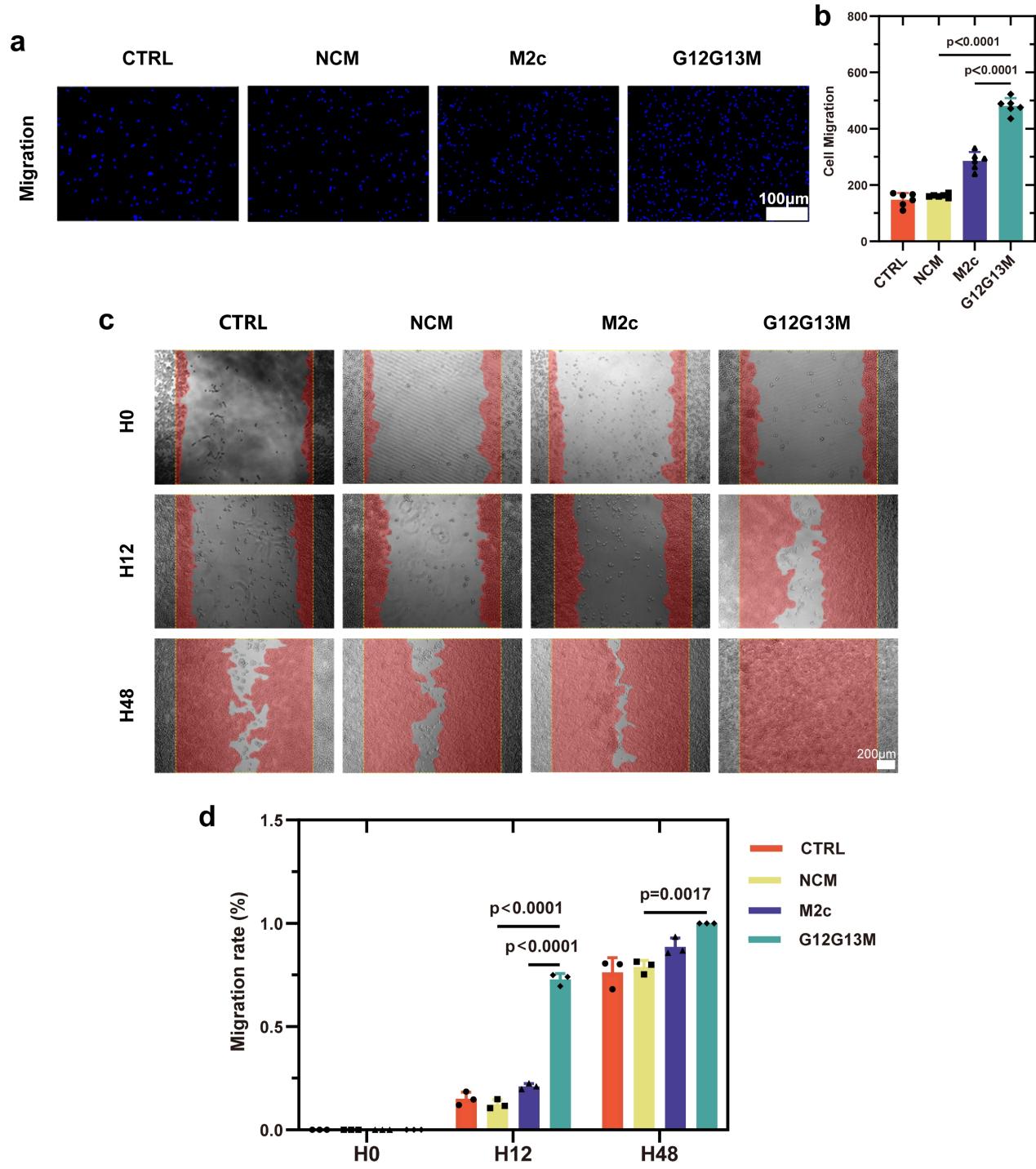


Supporting Figure 3. Enhanced migratory capacity of GNA12/GNA13-overexpressing macrophages.

(a) Representative images of transwell migration assays in CTRL, NCM, M2c, and G12G13M macrophages. G12G13M macrophages exhibit markedly increased migratory behavior. Scale bar: 100 µm.

(b) Quantification of migrated cells, showing significantly elevated migration in the G12G13M group (n = 6).

(c) Wound healing assay images at 0 h, 12 h, and 48 h. The red-shaded regions denote wound areas. G12G13M macrophages display accelerated wound closure. Scale bar: 200 µm.

(d) Migration rate analysis at different time points, confirming enhanced motility in G12G13M macrophages (n = 3).

Data are presented as mean ± SD. One-way ANOVA (b) and repeated-measures two-way ANOVA (d) followed by Tukey’s test were used for statistical analysis. Exact *p-*values are shown in the figure.


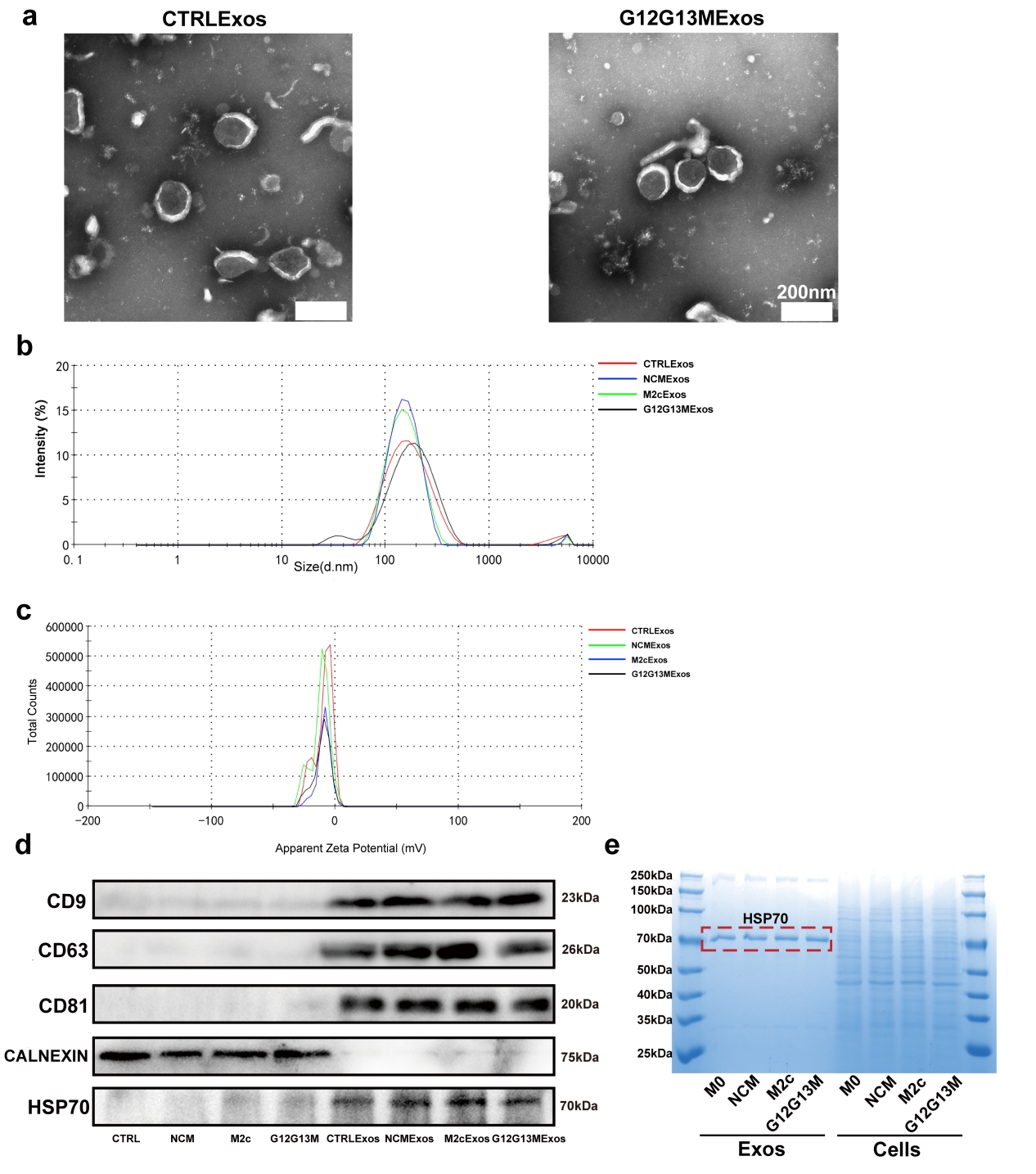


Supporting Figure 4. Characterization of exosomes derived from GNA12/GNA13-overexpressing macrophages (G12G13MExos).

(a) Transmission electron microscopy (TEM) images of exosomes isolated from control macrophages (M0Exos) and G12G13MExos, showing typical cup-shaped morphology. Scale bar: 200 µm.

(b) Nanoparticle tracking analysis (NTA) of exosome size distribution in different groups (CTRLExos, NCMExos, M2cExos, and G12G13MExos) (n = 3).

(c) Zeta potential distribution of exosomes from each group, indicating comparable surface charge profiles (n = 3).

(d) Western blot analysis of exosomal markers (CD9, CD63, CD81), cytosolic marker HSP70, and negative marker CALNEXIN. Positive expression of exosomal markers and absence of CALNEXIN confirm exosome purity. Representative results from three independent experiments are shown (n = 3).

(e) Coomassie brilliant blue staining of total protein from exosomes and corresponding cells. Enrichment of HSP70 is evident in exosome fractions but not in cellular lysates. Representative data from (n = 3).

Data are presented as mean ± SD.


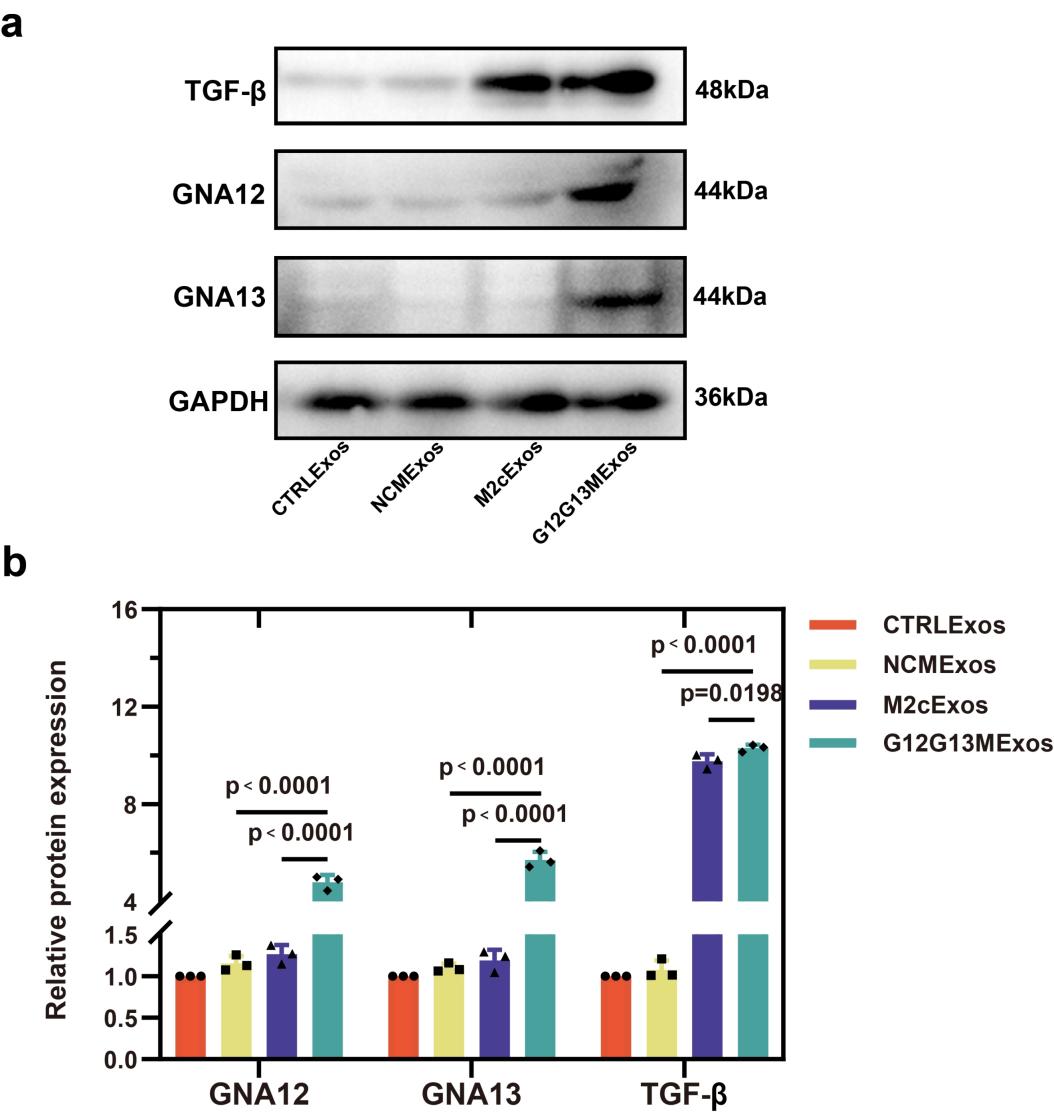


Supporting Figure 5. Western blot analysis of protein enrichment in exosomes from different macrophage groups.

(a) Representative Western blot images showing the expression of GNA12, GNA13, and TGF-β in exosomes derived from CTRL, NCM, M2c, and G12G13M macrophages. GAPDH was used as the loading control.

(b) Quantification of relative protein expression levels of GNA12, GNA13, and TGF-β. G12G13MExos showed significantly elevated levels of all three proteins compared to other groups (n = 3).

Data are presented as mean ± SD. Statistical analysis was performed using one-way ANOVA followed by Tukey’s multiple comparisons test. Exact *p-*values are shown in the figure.


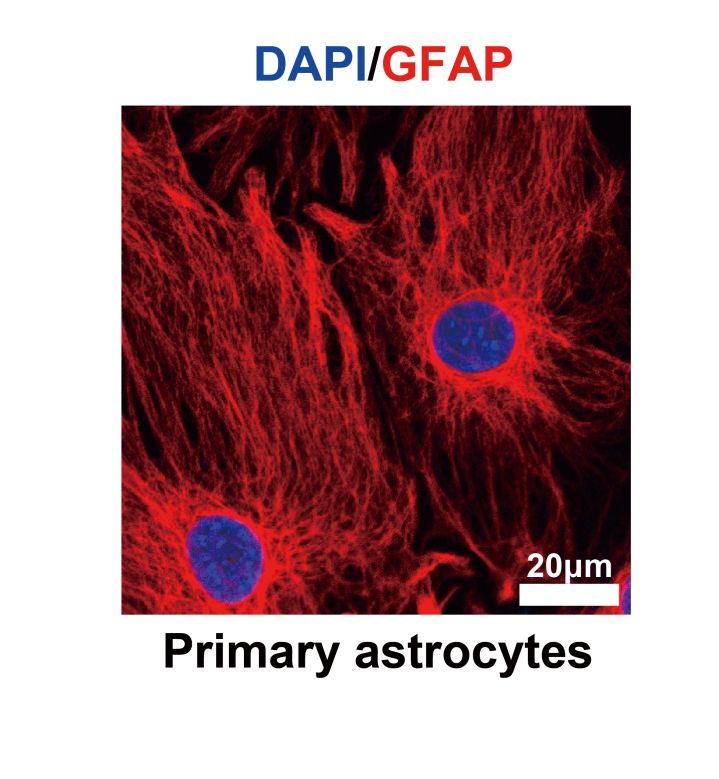


Supporting Figure 6. Immunofluorescence identification of primary astrocytes.

Representative confocal image of primary astrocytes stained with DAPI (blue) and GFAP (red), an astrocyte-specific intermediate filament protein. Robust GFAP expression confirms the purity and identity of the cultured astrocytes. Scale bar: 20 µm.


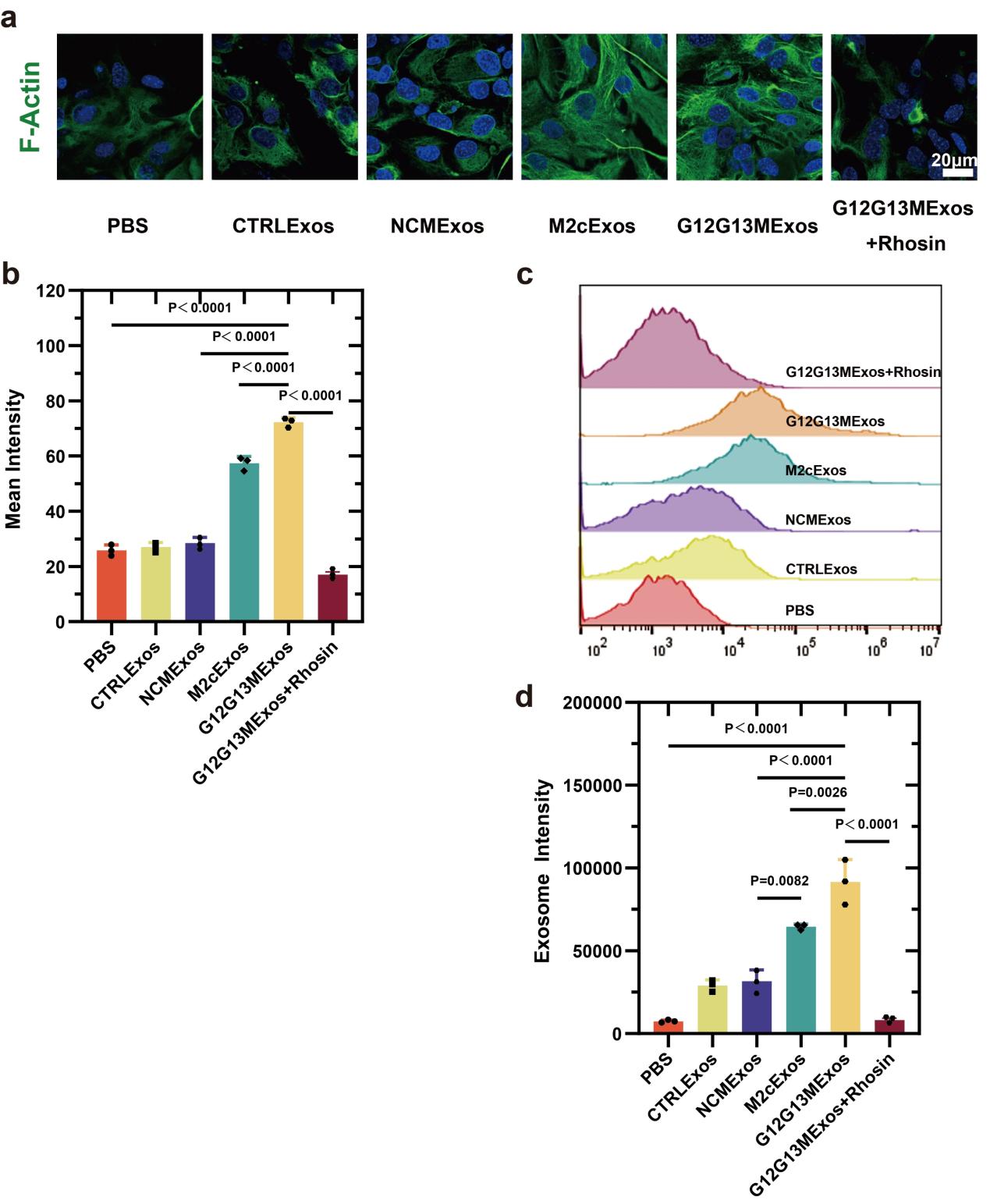


Supporting Figure 7. Enhanced astrocytic uptake of G12G13MExos and its inhibition by Rhosin.

(a) Representative confocal images of primary astrocytes treated with PBS, CTRLExos, NCMExos, M2cExos, G12G13MExos, or G12G13MExos + Rhosin. F-actin was stained with phalloidin (green) and nuclei with DAPI (blue). G12G13MExos treatment induced pronounced cytoskeletal remodeling, which was suppressed by Rhosin. Scale bar: 20 µm.

(b) Quantification of F-actin mean fluorescence intensity. G12G13MExos significantly increased actin polymerization compared to other groups, which was reversed by Rhosin treatment (n = 3).

(c) Flow cytometry histograms showing exosome uptake by astrocytes in different treatment groups. G12G13MExos exhibited the highest cellular internalization.

(d) Quantification of cellular exosome intensity from flow cytometry data, confirming enhanced uptake of G12G13MExos, attenuated by Rhosin (n = 3).

Data are presented as mean ± SD. Statistical analysis was performed using one-way ANOVA followed by Tukey’s multiple comparisons test. Exact *p-*values are shown in the figure.


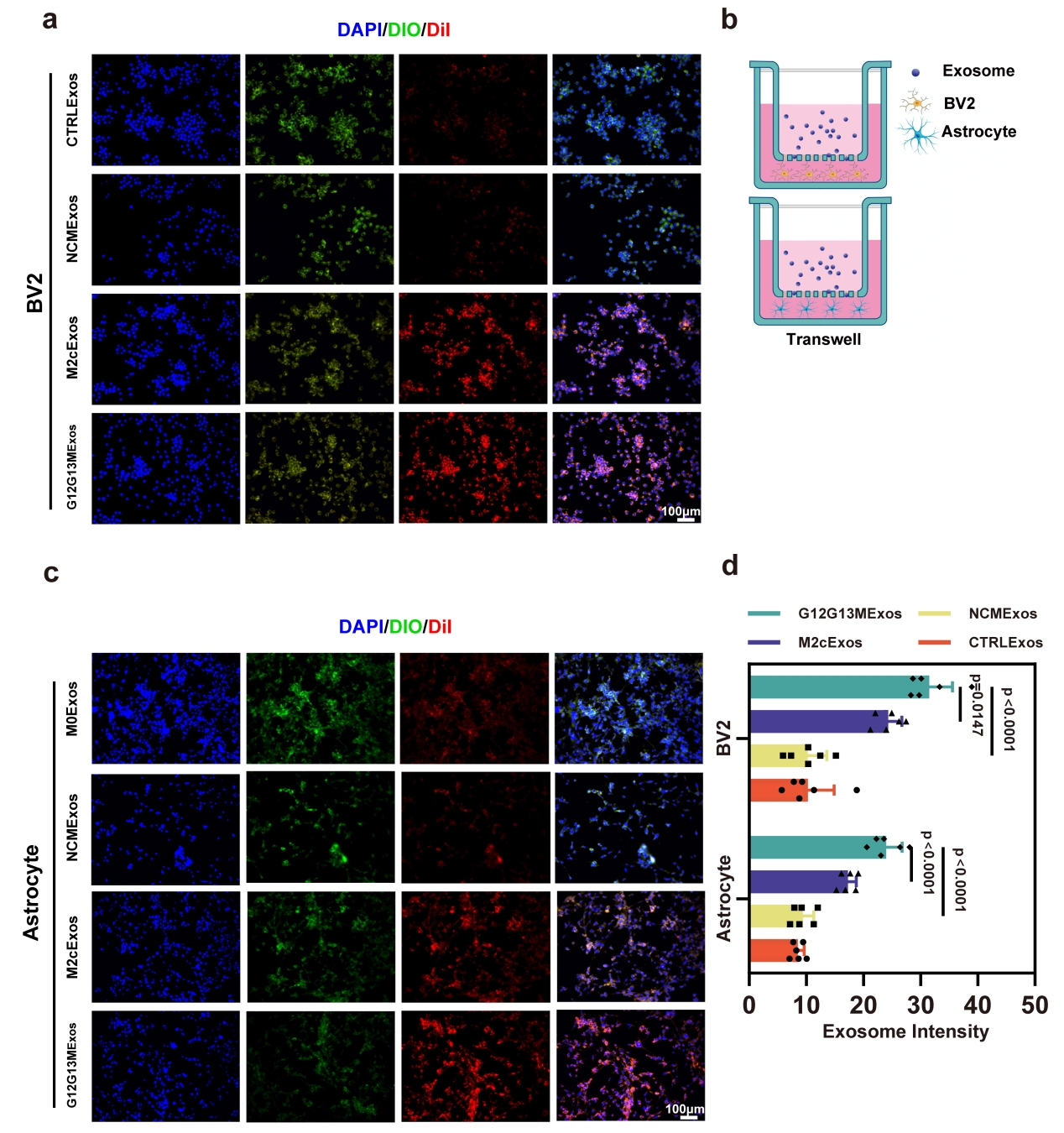


Supporting Figure 8. Uptake of G12G13MExos by BV2 microglia and primary astrocytes in a transwell co-culture system.

(a) Representative immunofluorescence images of BV2 microglia treated with DiO (green)/DiI (red)-labeled exosomes. Nuclei were counterstained with DAPI (blue). G12G13MExos-treated microglia exhibited the highest level of dual-labeled exosome uptake. Scale bar: 100 µm.

(b) Schematic diagram of the transwell co-culture model used to assess exosome transfer from upper-chamber cells to lower-chamber astrocytes or microglia.

(c) Representative immunofluorescence images of primary astrocytes showing uptake of DiO/DiI-labeled exosomes from different groups. G12G13MExos treatment resulted in the strongest exosome accumulation. Scale bar: 100 µm.

(d) Quantification of intracellular exosome fluorescence intensity in BV2 microglia and astrocytes. G12G13MExos showed significantly higher uptake in both cell types (n = 6).

Data are presented as mean ± SD. Statistical analysis was performed using one-way ANOVA followed by Tukey’s multiple comparisons test. Exact *p-*values are shown in the figure.


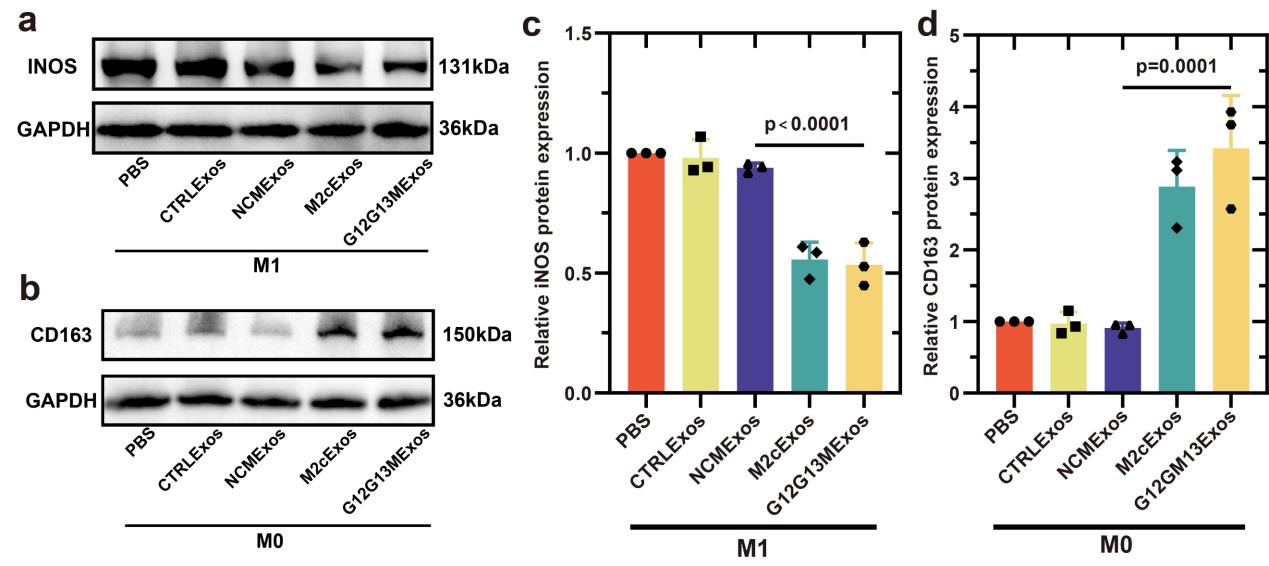


Supporting Figure 9. G12G13MExos modulate macrophage polarization by inhibiting M1 markers and promoting M2c markers.

(a) Western blot analysis of iNOS, a proinflammatory M1 macrophage marker, in macrophages treated with PBS, CTRLExos, NCMExos, M2cExos, or G12G13MExos. GAPDH was used as a loading control.

(b) Western blot analysis of CD163, a characteristic marker of M2c macrophages, under the same treatment conditions.

(c) Quantification of iNOS protein levels in M1-polarized macrophages, showing significantly reduced expression in the G12G13MExos group (n = 3).

(d) Quantification of CD163 expression in M0 macrophages, demonstrating enhanced M2c polarization following G12G13MExos treatment (n = 3).

Data are presented as mean ± SD. Statistical analysis was performed using one-way ANOVA followed by Tukey’s multiple comparisons test. Exact *p-*values are shown in the figure.


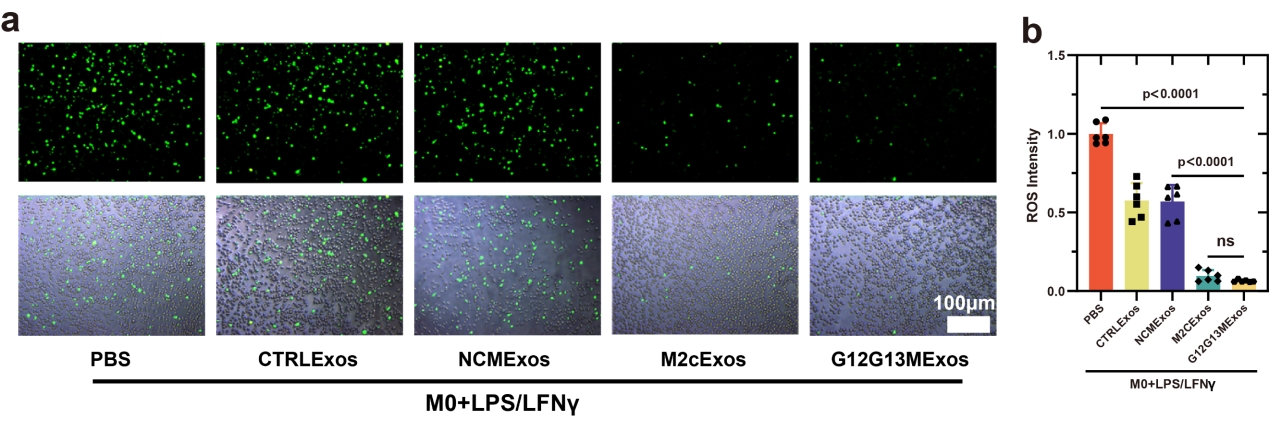


Supporting Figure 10. G12G13MExos attenuate reactive oxygen species (ROS) production in M1-polarized macrophages.

(a) Representative fluorescence and corresponding bright-field images of ROS levels detected by DCFH-DA staining in M1 macrophages (M0 + LPS/IFN-γ) treated with PBS, CTRLExos, NCMExos, M2cExos, or G12G13MExos. A marked reduction in fluorescence intensity was observed in the G12G13MExos group. Scale bar: 100 µm.

(b) Quantification of ROS fluorescence intensity across different treatment groups. G12G13MExos significantly reduced ROS levels compared to NCMExos and CTRLExos (n = 6).

Data are presented as mean ± SD. Statistical analysis was performed using one-way ANOVA followed by Tukey’s multiple comparisons test. Exact *p-*values are shown in the figure. ns, not significant.


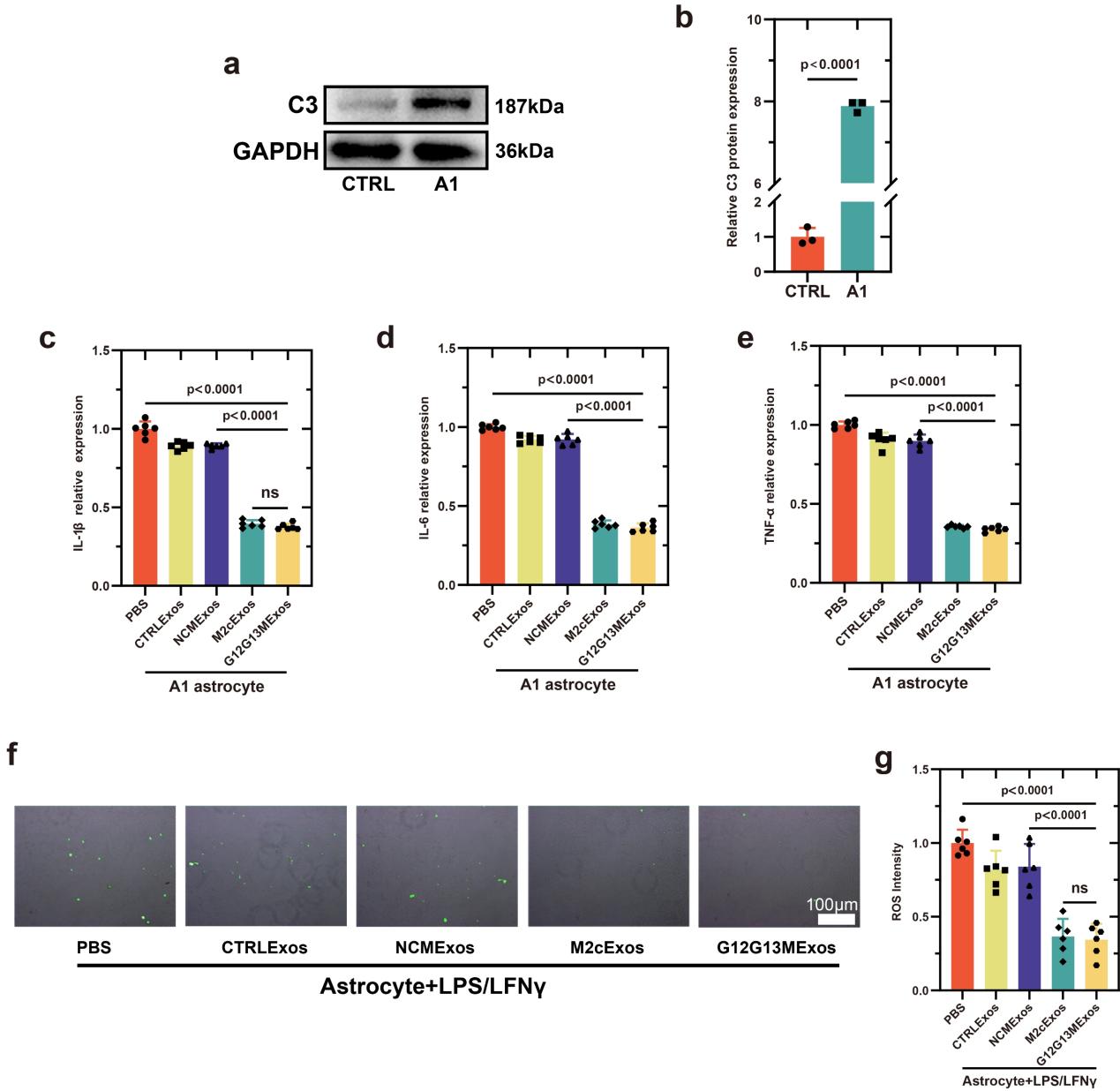


Supporting Figure 11. G12G13MExos attenuate proinflammatory responses and oxidative stress in A1 astrocytes.

(a) Western blot analysis of complement component C3 in A1 astrocytes (LPS + IFN-γ stimulated) versus control astrocytes (n = 3).

(b) Quantification of C3 protein expression, confirming successful induction of the A1 reactive astrocyte phenotype (n = 6).

(c–e) qPCR analysis of proinflammatory cytokine expression in A1 astrocytes treated with PBS, CTRLExos, NCMExos, M2cExos, or G12G13MExos. G12G13MExos significantly suppressed expression of Il-1β (c), Il-6 (d), and Tnf-α (e) (n = 6).

(f) Representative fluorescence and bright-field images of ROS levels detected by DCFH-DA staining in A1 astrocytes under various treatments. G12G13MExos markedly reduced ROS accumulation. Scale bar: 100 µm.

(g) Quantification of ROS fluorescence intensity in A1 astrocytes (n = 6).

Data are presented as mean ± SD. Statistical analysis was performed using one-way ANOVA followed by Tukey’s multiple comparisons test. Exact *p-*values are shown in the figure. ns, not significant.


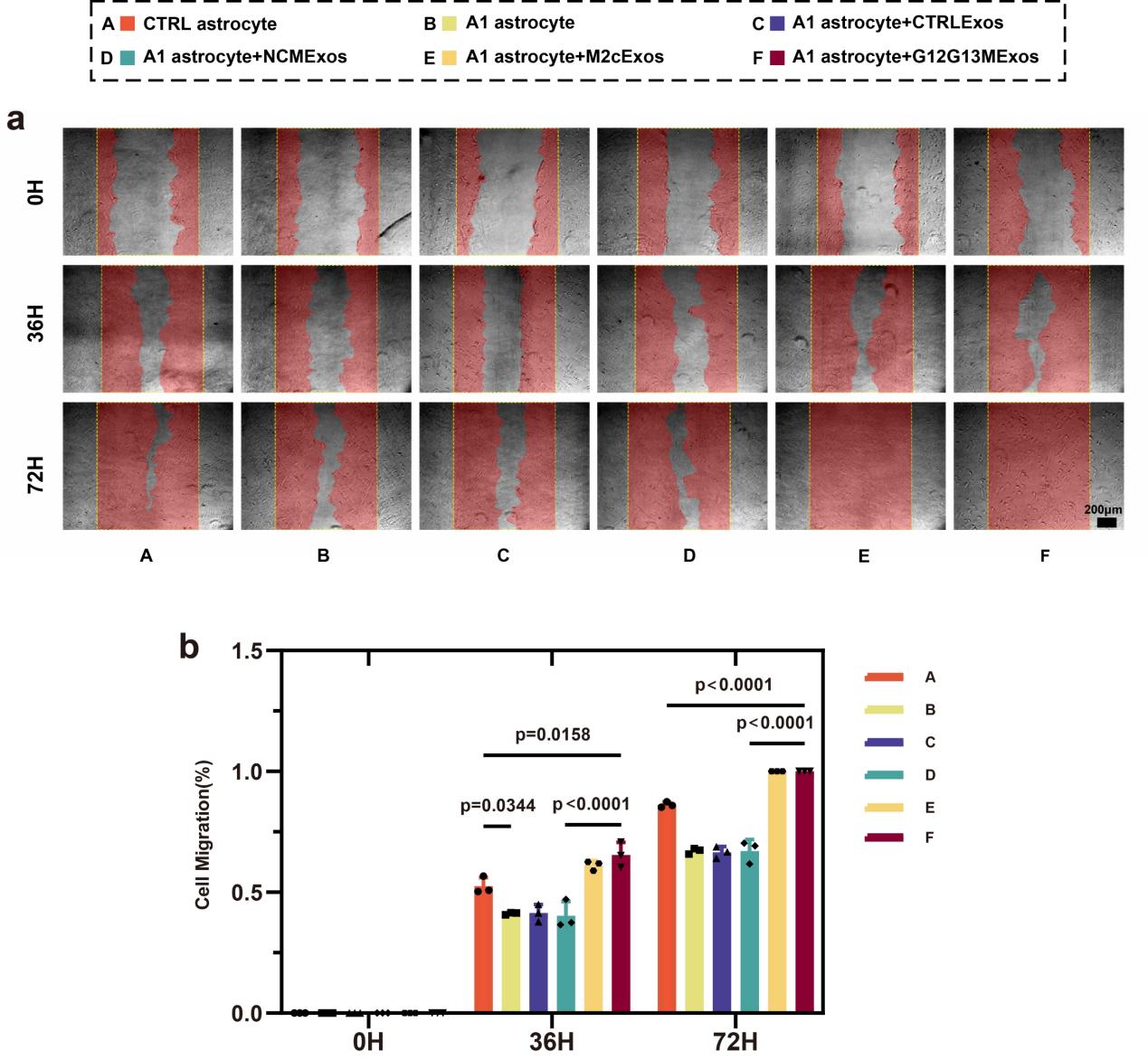


Supporting Figure 12. G12G13MExos promote the migration of A1 astrocytes in vitro.

(a) Representative wound healing assay images showing the migration of A1 astrocytes under different treatments at 0 h, 36 h, and 72 h. The red-shaded region indicates the initial wound area. A1 astrocytes treated with G12G13MExos (group F) exhibited the most pronounced wound closure. Scale bar: 200 µm.

(b) Quantification of migration rates at the indicated time points. G12G13MExos significantly enhanced migration compared to NCMExos (group D) and CTRLExos (group C) (n = 6).

Data are presented as mean ± SD. Repeated-measures two-way ANOVA followed by Tukey’s test were used for statistical analysis. Exact *p-*values are shown in the figure.


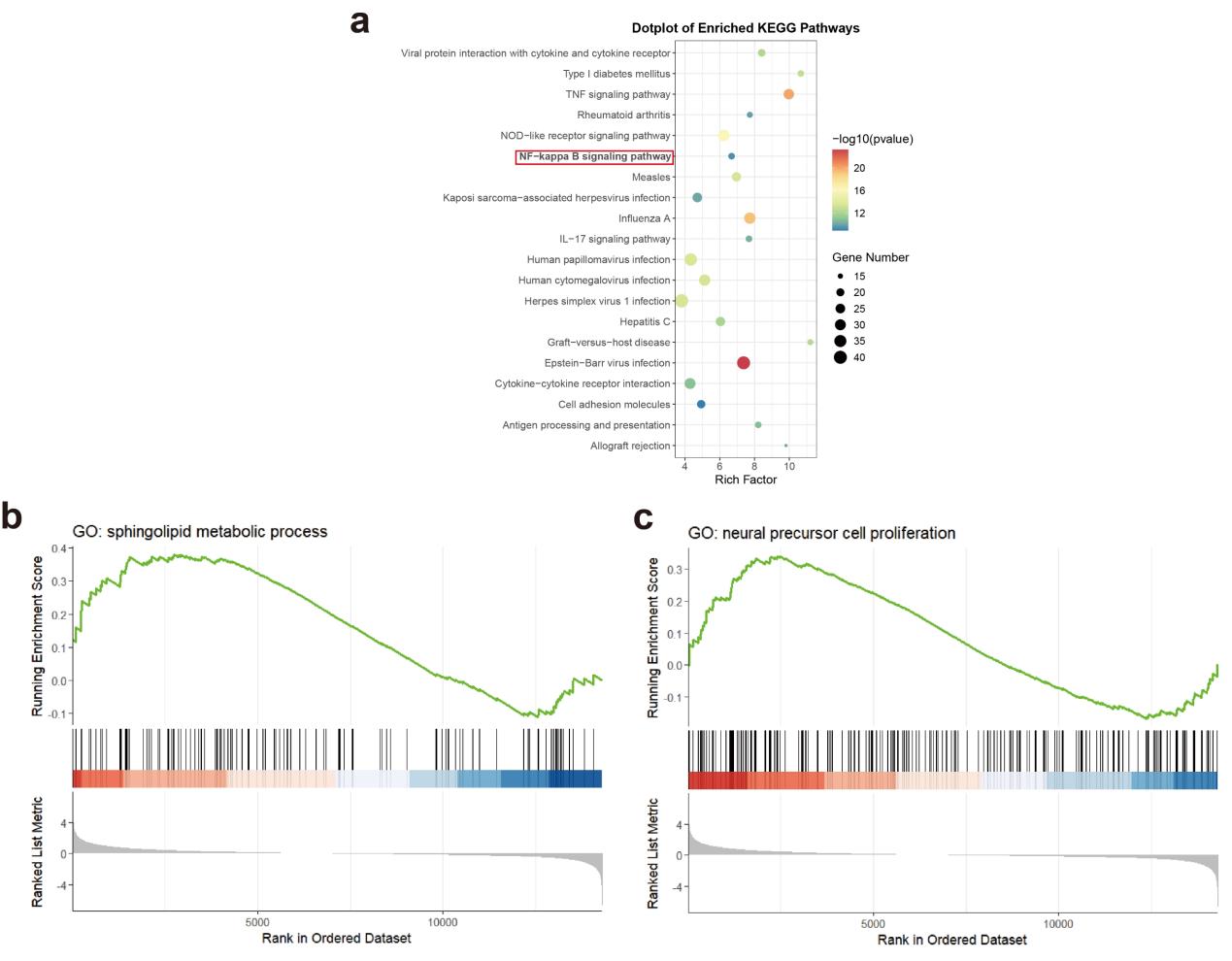


Supporting Figure 13. Transcriptomic enrichment analysis of G12G13MExos-treated astrocytes.

(a) KEGG pathway enrichment of DEGs in astrocytes treated with G12G13MExos. The NF-κB signaling pathway is significantly enriched (highlighted in red), suggesting its potential involvement in astrocyte regulation.

(b, c) GSEA of biological processes enriched in G12G13MExos-treated astrocytes.

(b) GO: Sphingolipid metabolic process, indicating possible modulation of lipid metabolic pathways.

(c) GO: Neural precursor cell proliferation, implying a potential role in neurogenesis and cellular repair mechanisms.

Statistical significance is represented by normalized enrichment score (NES) and false discovery rate (FDR) calculated by GSEA; ranked gene list metrics are shown below each plot.


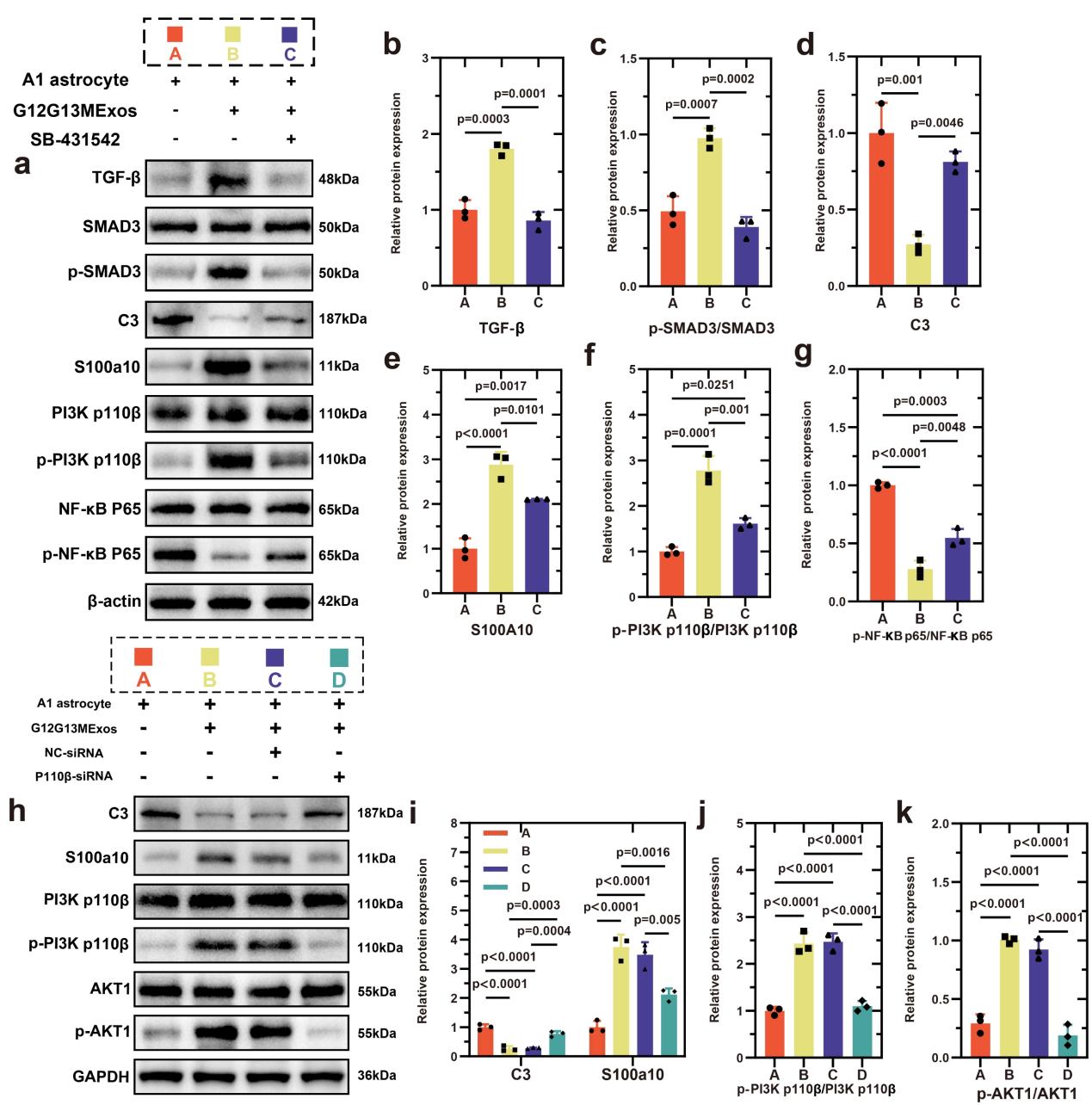


Supporting Figure 14. G12G13MExos modulate A1 astrocyte polarization through TGF-β/SMAD3 and PI3K/AKT/NF-κB signaling pathways.

(a–g) Western blot analysis of key signaling proteins in A1 astrocytes treated with G12G13MExos, with or without the TGF-β receptor inhibitor SB-431542.

(a) Representative blots showing expression levels of TGF-β, SMAD3, p-SMAD3, C3, S100A10, PI3K p110β, p-PI3K p110β, NF-κB p65, and p-NF-κB p65.

(b–g) Quantification of protein expression: TGF-β (b), p-SMAD3/SMAD3 (c), C3 (d), S100A10 (e), p-PI3K p110β/PI3K p110β (f), and p-NF-κB p65/NF-κB p65 (g) (n = 3).

(h–k) Evaluation of the effects of PI3K p110β knockdown on G12G13MExos-mediated astrocyte responses.

(h) Western blot analysis of C3, S100A10, PI3K p110β, p-PI3K p110β, AKT1, and p-AKT1 in A1 astrocytes transfected with NC-siRNA or PI3K p110β-siRNA.

(i–k) Quantification of protein expression: C3 and S100A10 (i), p-PI3K p110β/PI3K p110β (j), and p-AKT1/AKT1 (k) (n = 3).

Data are presented as mean ± SD. Statistical analysis was performed using one-way ANOVA followed by Tukey’s multiple comparisons test. Exact *p-*values are shown in the figure.


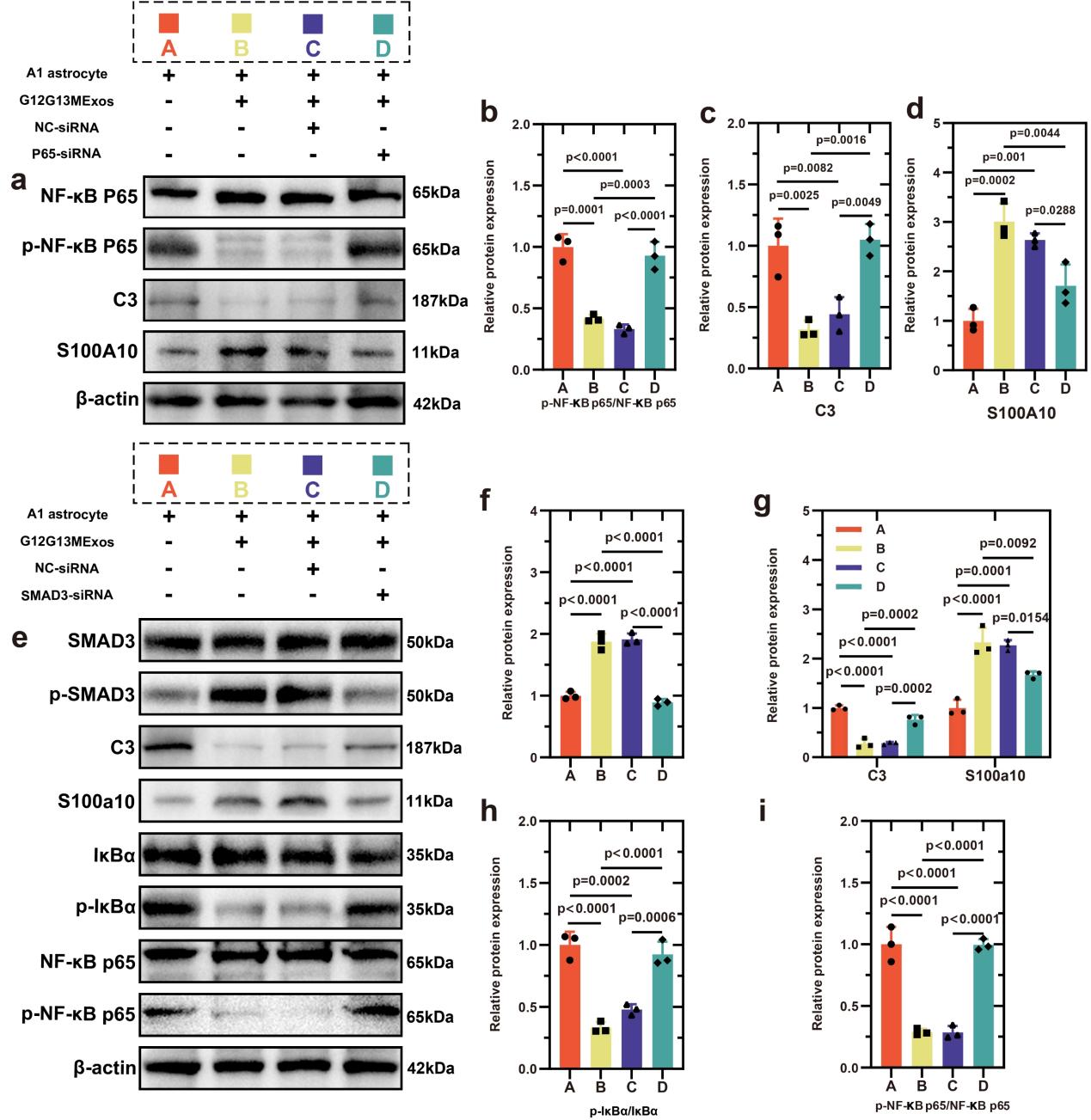


Supporting Figure 15. G12G13MExos regulate A1 astrocyte polarization via NF-κB and SMAD3 signaling pathways.

(a–d) Effects of NF-κB p65 knockdown on G12G13MExos-induced astrocyte reprogramming.

(a) Western blot analysis of NF-κB p65, p-NF-κB p65, C3, and S100A10 expression in A1 astrocytes transfected with control siRNA or p65-siRNA in the presence of G12G13MExos.

(b–d) Quantification of p-NF-κB p65/NF-κB p65 (b), C3 (c), and S100A10 (d) expression (n = 3).

(e–i) Effects of SMAD3 knockdown on G12G13MExos-mediated NF-κB pathway regulation.

(e) Western blot analysis of SMAD3, p-SMAD3, C3, S100A10, IκBα, p-IκBα, NF-κB p65, and p-NF-κB p65 in A1 astrocytes transfected with control siRNA or SMAD3-siRNA.

(f–i) Quantification of p-SMAD3/SMAD3 (f), C3 and S100A10 (g), p-IκBα/IκBα (h), and p-NF-κB p65/NF-κB p65 (i) (n = 3).

Data are presented as mean ± SD. Statistical analysis was performed using one-way ANOVA followed by Tukey’s multiple comparisons test. Exact *p-*values are shown in the figure.


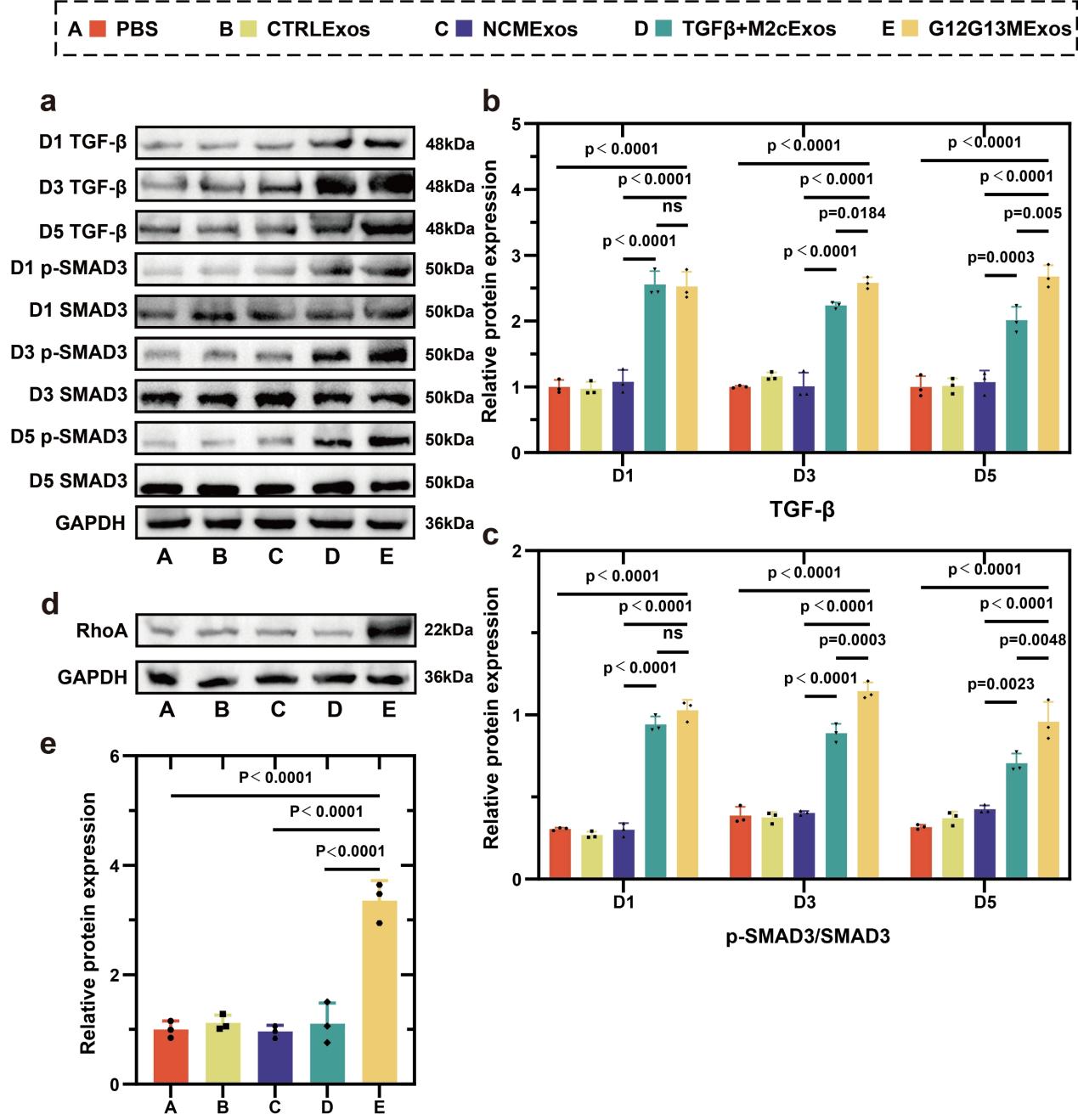


Supporting Figure 16. G12G13MExos activate the TGF-β/SMAD3 signaling pathway in astrocytes over time.

(a) Western blot analysis of TGF-β, p-SMAD3, and SMAD3 protein levels in astrocytes on days 1 (D1), 3 (D3), and 5 (D5) after treatment with PBS, CTRLExos, NCMExos, TGF-β+M2cExos, or G12G13MExos. GAPDH served as the internal control.

(b, c) Quantification of TGF-β (b) and p-SMAD3/SMAD3 (c) expression at D1, D3, and D5. G12G13MExos progressively increased activation of the TGF-β/SMAD3 axis (n = 3).

(d) Western blot analysis of RhoA protein expression under the same treatment conditions.

(e) Quantification of RhoA expression at D1, showing significantly elevated levels in G12G13MExos-treated astrocytes (n = 3).

Data are presented as mean ± SD. Statistical analysis was performed using one-way ANOVA followed by Tukey’s multiple comparisons test. Exact *p-*values are shown in the figure. ns, not significant.


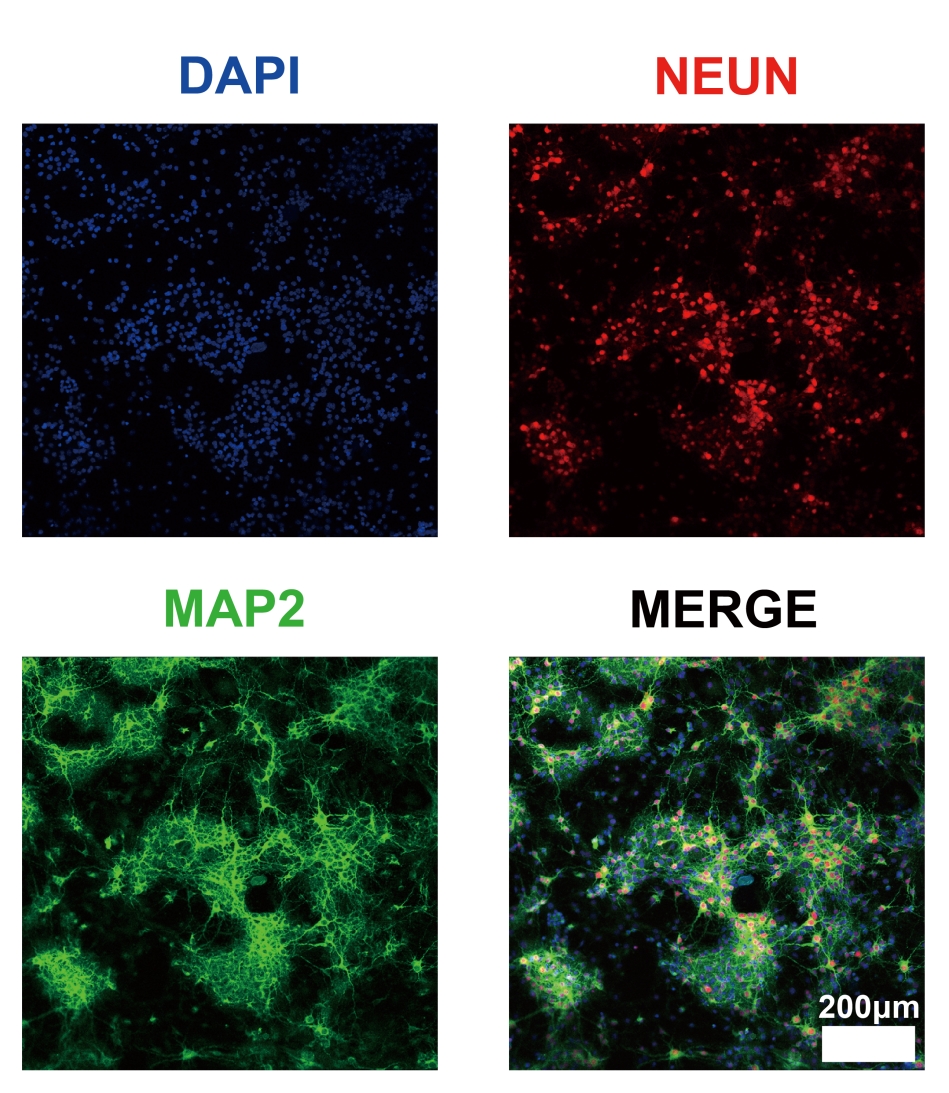


Supporting Figure 17. Immunofluorescence characterization of primary cultured neurons.

Representative images of primary neurons stained with NEUN (red, neuronal nuclei marker), MAP2 (green, dendritic marker), and DAPI (blue, nuclear stain). Co-localization of NEUN and MAP2 signals in the merged panel confirms the neuronal identity and dendritic structure of the cultured cells. Scale bar: 200 µm.


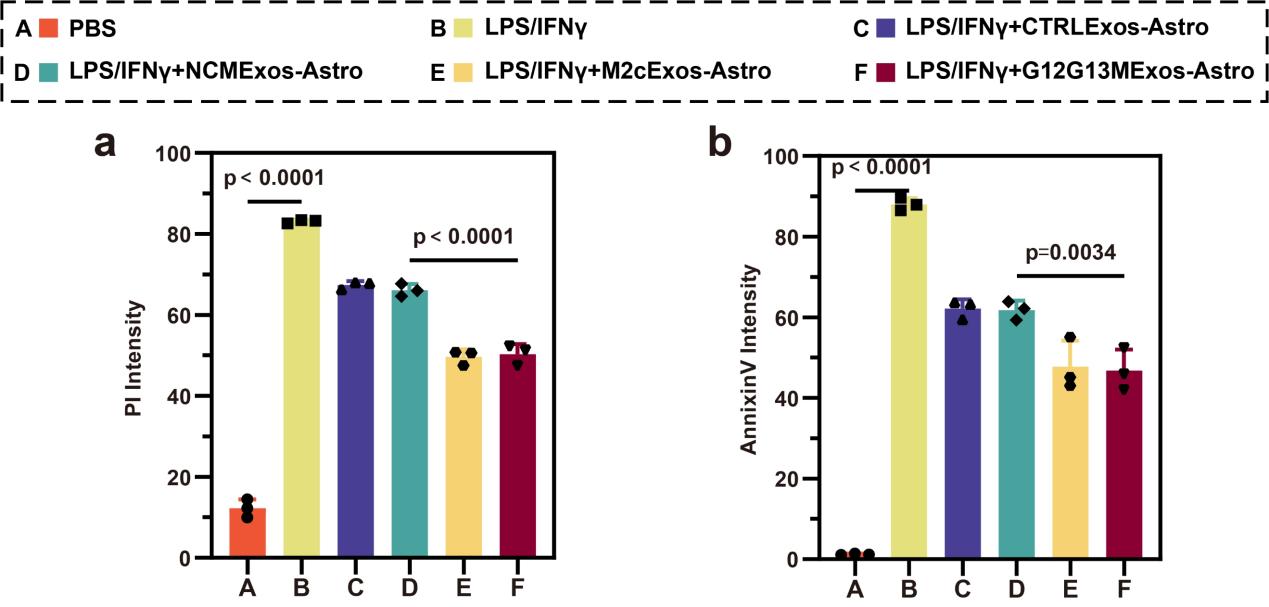


Supporting Figure 18. G12G13MExos-treated astrocytes attenuate neuronal apoptosis.

(a, b) Quantification of neuronal apoptosis in co-culture systems using propidium iodide (PI) staining (a) and Annexin V staining (b). Neurons were co-cultured with astrocytes pretreated with PBS, LPS/IFN-γ, or exosomes (NCMExos, CTRLExos, M2cExos, or G12G13MExos). Astrocytes treated with G12G13MExos significantly reduced both PI and Annexin V fluorescence intensities, indicating decreased neuronal apoptosis (n = 3).

Data are presented as mean ± SD. Statistical analysis was performed using one-way ANOVA followed by Tukey’s multiple comparisons test. Exact *p-*values are shown in the figure.


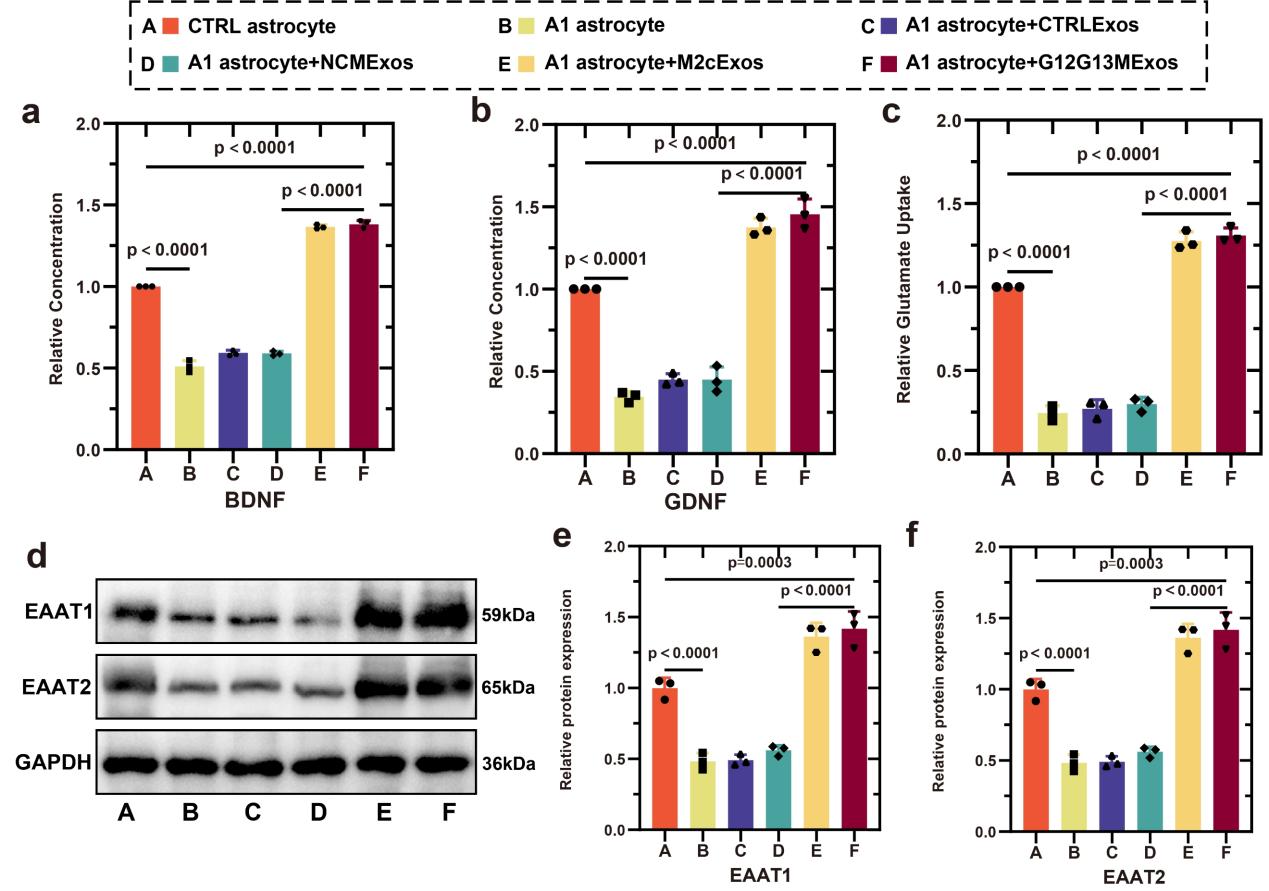


Supporting Figure 19. G12G13MExos-treated astrocytes enhance neurotrophic factor secretion and glutamate clearance.

(a, b) Quantification of neurotrophic factors in astrocyte cultures. G12G13MExos significantly increased the secretion of BDNF (a) and GDNF (b) (n = 3).

(c) Measurement of relative glutamate uptake levels in astrocytes under different treatments. G12G13MExos enhanced glutamate clearance compared to control groups (n = 3).

(d) Western blot analysis of EAAT1 and EAAT2 expression, key astrocytic glutamate transporters.

(e, f) Quantification of EAAT1 (e) and EAAT2 (f) expression confirms that G12G13MExos upregulate glutamate transporter levels (n = 3).

Data are presented as mean ± SD. Statistical analysis was performed using one-way ANOVA followed by Tukey’s multiple comparisons test. Exact *p-*values are shown in the figure.


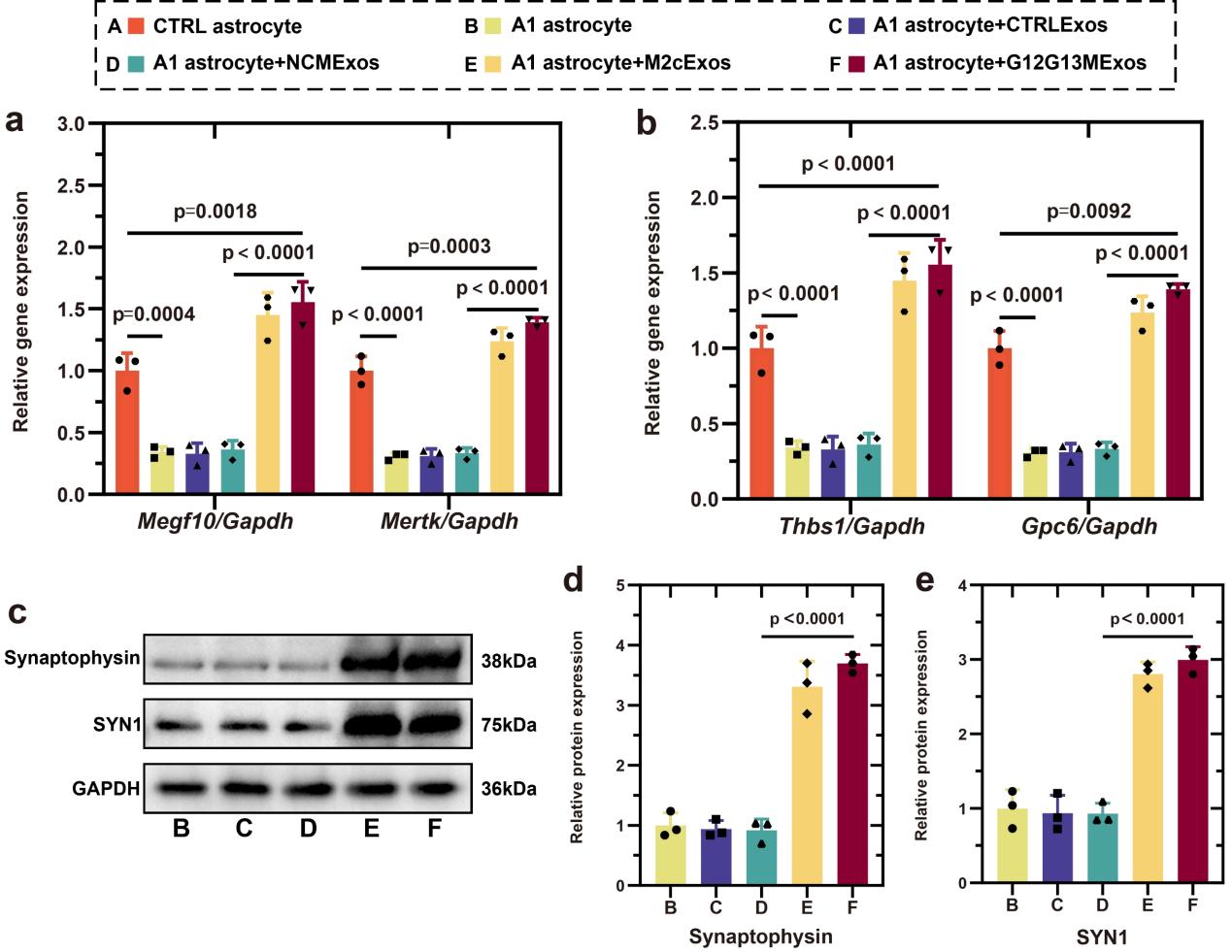


Supporting Figure 20. Supporting Figure 20. G12G13MExos promote astrocyte-mediated synaptic support and neuroprotection.

(a, b) Quantitative PCR analysis of synapse-supportive and phagocytic genes in astrocytes treated with different exosomes. G12G13MExos significantly upregulated *Megf10* and *Mertk* (a), as well as *Thbs1* and *Gpc6* (b), indicating enhanced neuroprotective potential (n = 3).

(c) Western blot analysis of the synaptic markers Synaptophysin and SYN1 across treatment groups. GAPDH was used as a loading control.

(d, e) Quantification of Synaptophysin (d) and SYN1 (e) expression levels. G12G13MExos markedly increased synaptic protein expression (n = 3).

Data are presented as mean ± SD. Statistical analysis was performed using one-way ANOVA followed by Tukey’s multiple comparisons test. Exact *p-*values are shown in the figure.


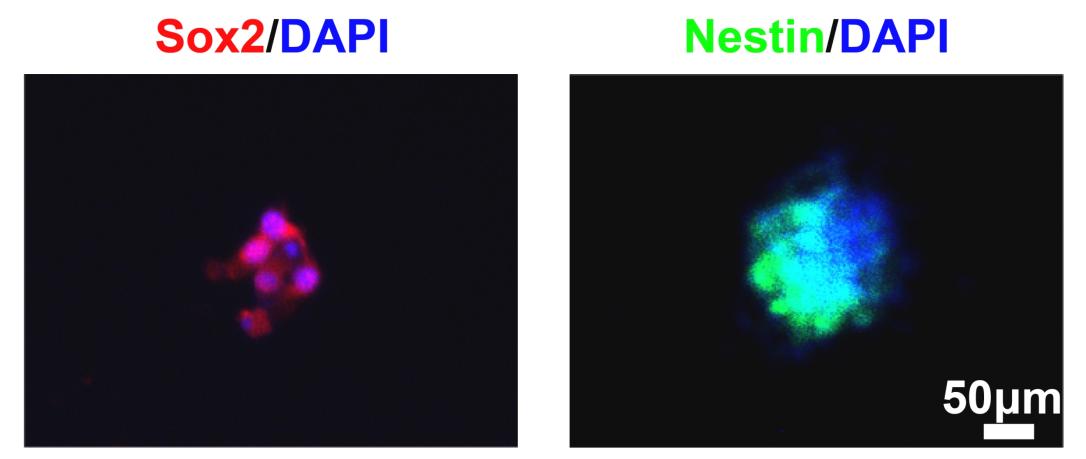


Supporting Figure 21. Immunofluorescence validation of neural stem cells (NSCs) identity.

Representative immunofluorescence images of cultured NSCs stained for Sox2 (red) and Nestin (green), both established markers of neural stemness. DAPI (blue) labels cell nuclei. Robust Sox2 and Nestin expression confirms the NSC phenotype. Scale bar: 50 µm.


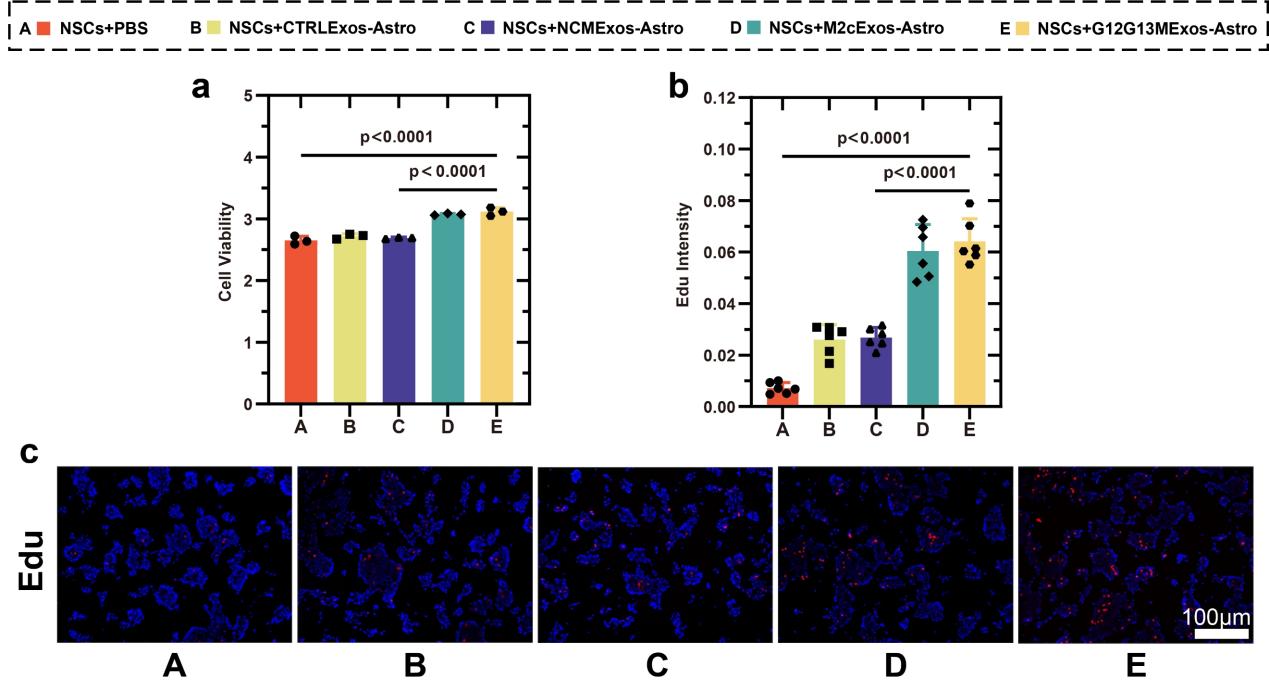


Supporting Figure 22. Astrocytes treated with G12G13MExos enhance NSC viability and proliferation

(a) Cell viability assay showing the survival of NSCs co-cultured with astrocytes treated with different exosome groups. Astrocytes treated with G12G13MExos significantly enhanced NSC viability (n = 3).

(b) Quantification of EdU incorporation in NSCs, indicating proliferative activity. G12G13MExos-treated astrocytes induced the highest EdU fluorescence intensity (n = 6).

(c) Representative immunofluorescence images of EdU (red) and DAPI (blue) staining in NSCs across different treatment groups. Scale bar: 100 μm.

Data are presented as mean ± SD. Statistical analysis was performed using one-way ANOVA followed by Tukey’s multiple comparisons test. Exact *p-*values are shown in the figure.


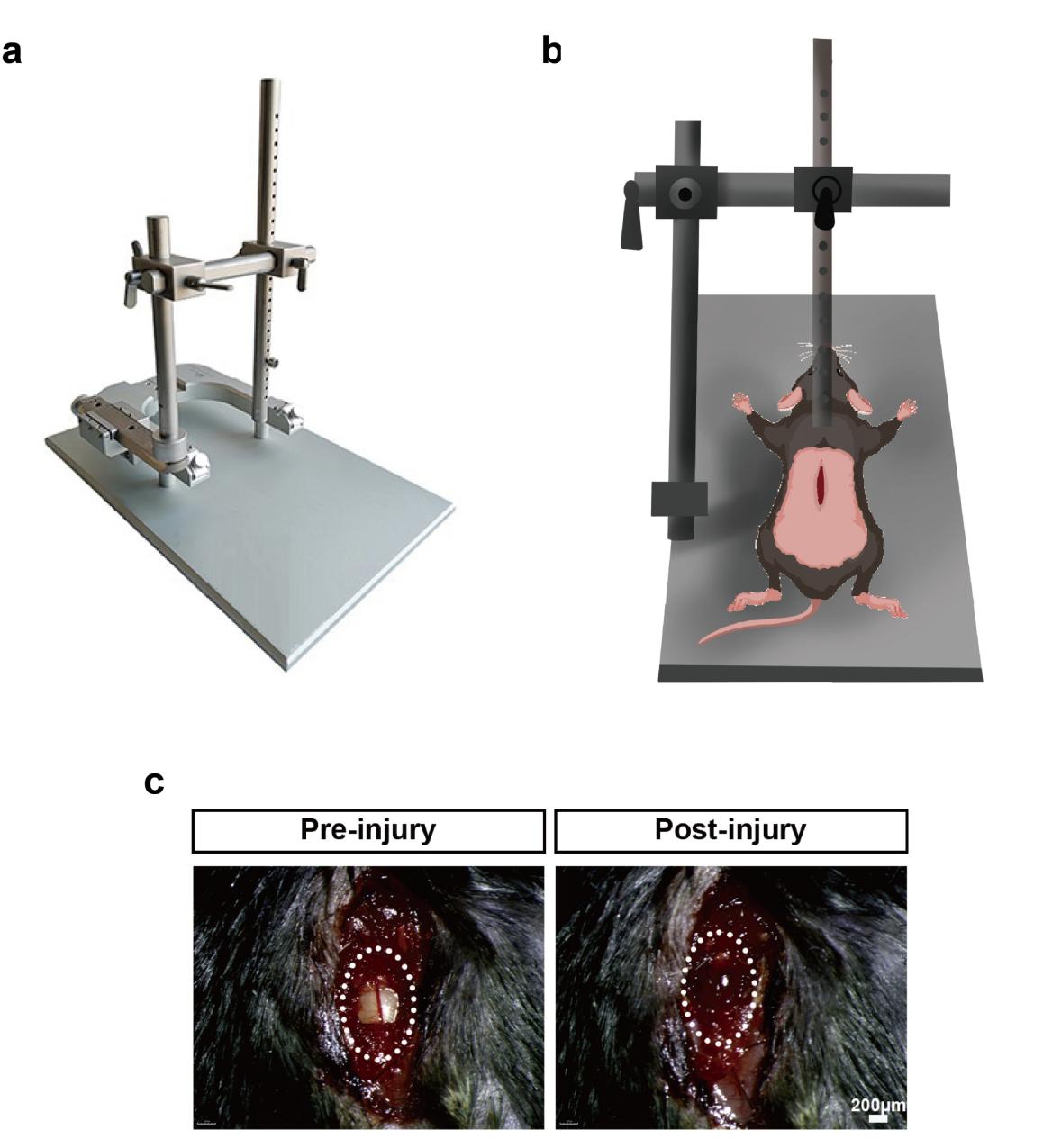


Supporting Figure 23. Establishment of the SCI model in C57BL/6 mice.

(a) Custom-built impactor device used to generate contusion SCI in mice.

(b) Schematic diagram showing mouse positioning and fixation under the impactor system prior to injury induction.

(c) Representative images of the spinal cord before (Pre-injury) and after (Post-injury) impact. The dotted lines delineate the laminectomy window and injury zone. Scale bar: 200 µm.


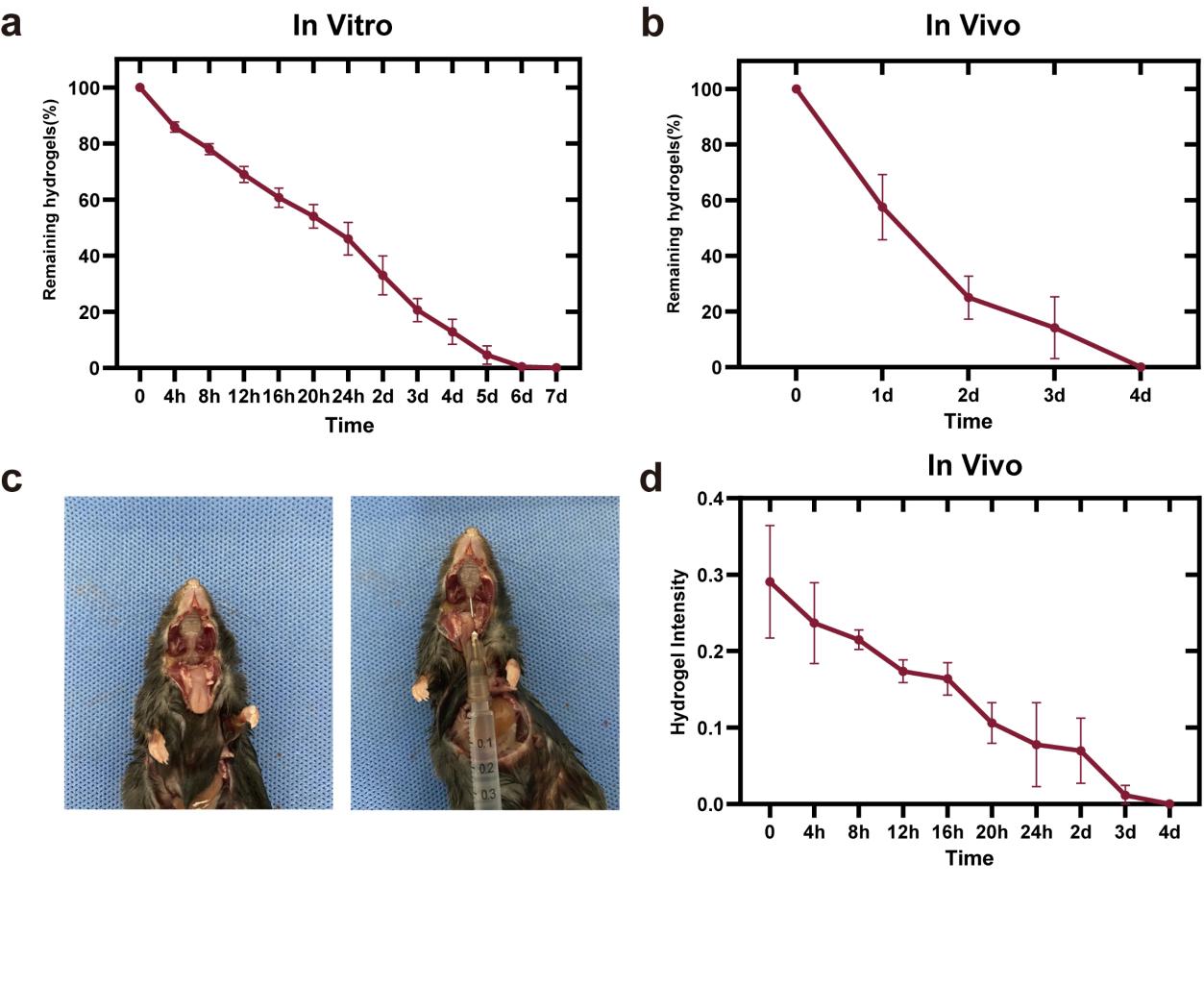


Supporting Figure 24. Degradation profile of the thermosensitive hydrogel in vitro and in vivo.

(a) In vitro hydrogel degradation curve under simulated nasal conditions (pH = 6.0, 34 °C, 1 mg/mL lysozyme), showing a time-dependent decrease in hydrogel mass over 7 days (n = 3).

(b) In vivo hydrogel degradation assessed by anatomical dissection and residual hydrogel weight following nasal administration in mice, up to 4 days (n = 3).

(c) Representative images of the nasal lavage procedure used to collect hydrogel residues from the nasal cavity.

(d) Quantification of in vivo hydrogel degradation by measuring fluorescent signal intensity from nasal lavage fluid at different time points post-administration (n = 3).

Data are presented as mean ± SD.


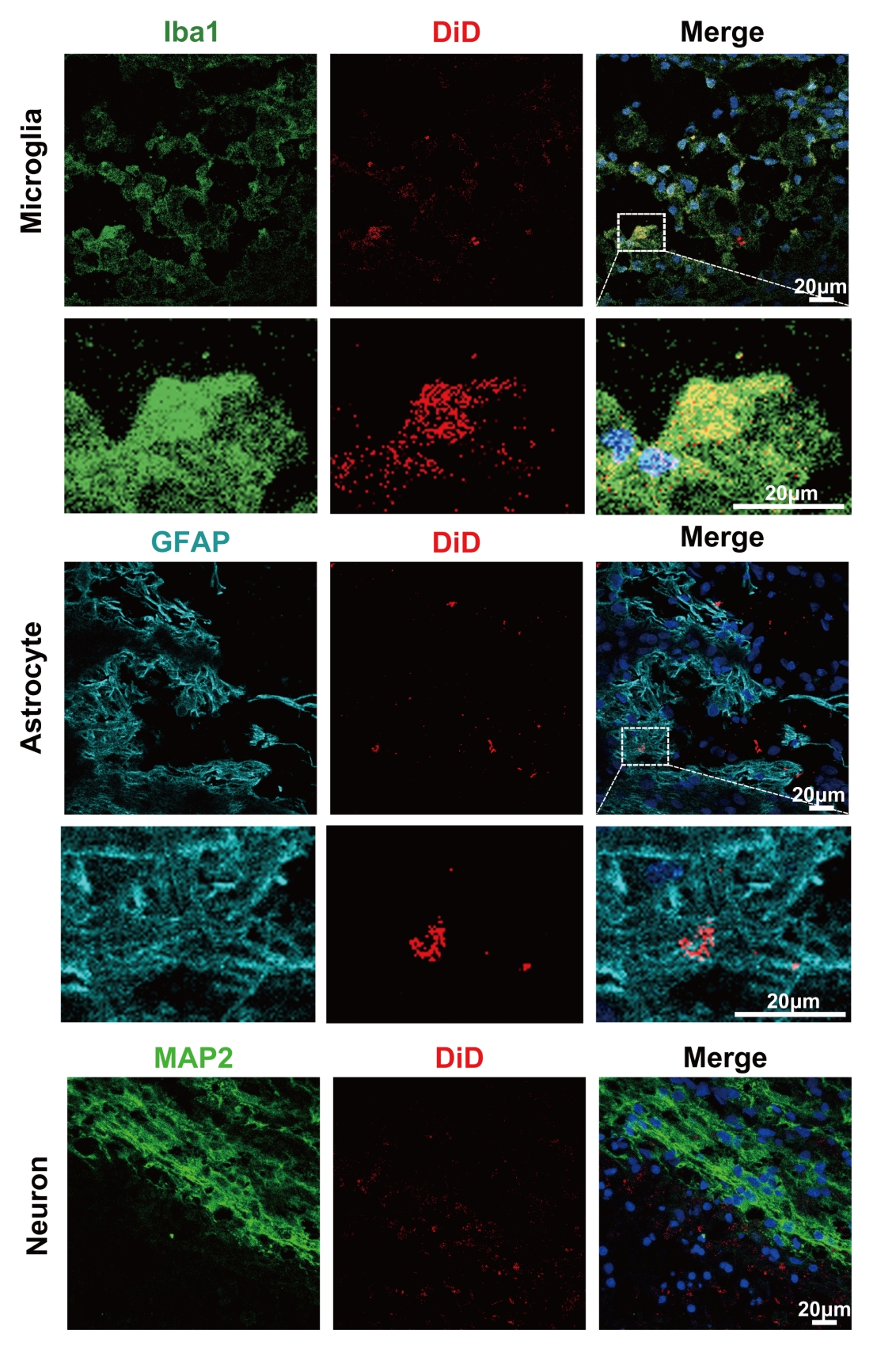


Supporting Figure 25. Cellular uptake of DiD-labeled exosomes in spinal cord tissue following nasal administration.

Representative immunofluorescence images showing co-localization of DiD-labeled exosomes (red) with specific cell markers in spinal cord tissue sections.

Top: Microglia labeled with Iba1 (green), indicating prominent exosome uptake.

Middle: Astrocytes labeled with GFAP (cyan), showing both localized and dispersed DiD signal within astrocytic domains.

Bottom: Neurons labeled with MAP2 (green), exhibiting relatively limited DiD signal co-localization.

DAPI (blue) stains cell nuclei. Scale bars: 20 μm.


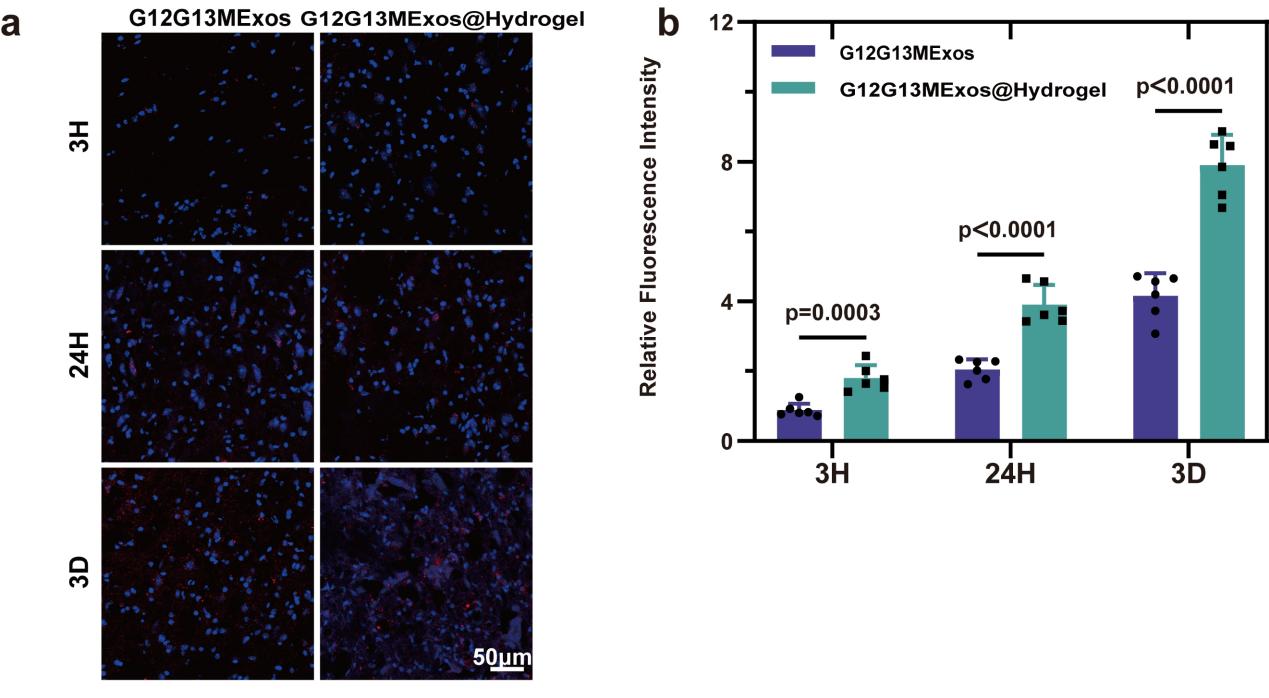


Supporting Figure 26. Time-dependent accumulation of DiD-labeled G12G13MExos in the spinal cord following nasal delivery.

(a) Representative fluorescence images showing the distribution of DiD-labeled G12G13MExos (red) in spinal cord tissue at 3 hours (3H), 24 hours (24H), and 3 days (3D) after nasal administration, with or without hydrogel encapsulation. DAPI (blue) stains nuclei. Scale bar: 50 μm.

(b) Quantification of relative DiD fluorescence intensity over time. G12G13MExos@Hydrogel exhibited significantly prolonged retention and higher fluorescence intensity in the spinal cord compared to free G12G13MExos (n = 6).

Data are presented as mean ± SD. Statistical comparisons between the two groups at each time point were performed using unpaired two-tailed t-tests. Exact *p*-values are shown in the figure.


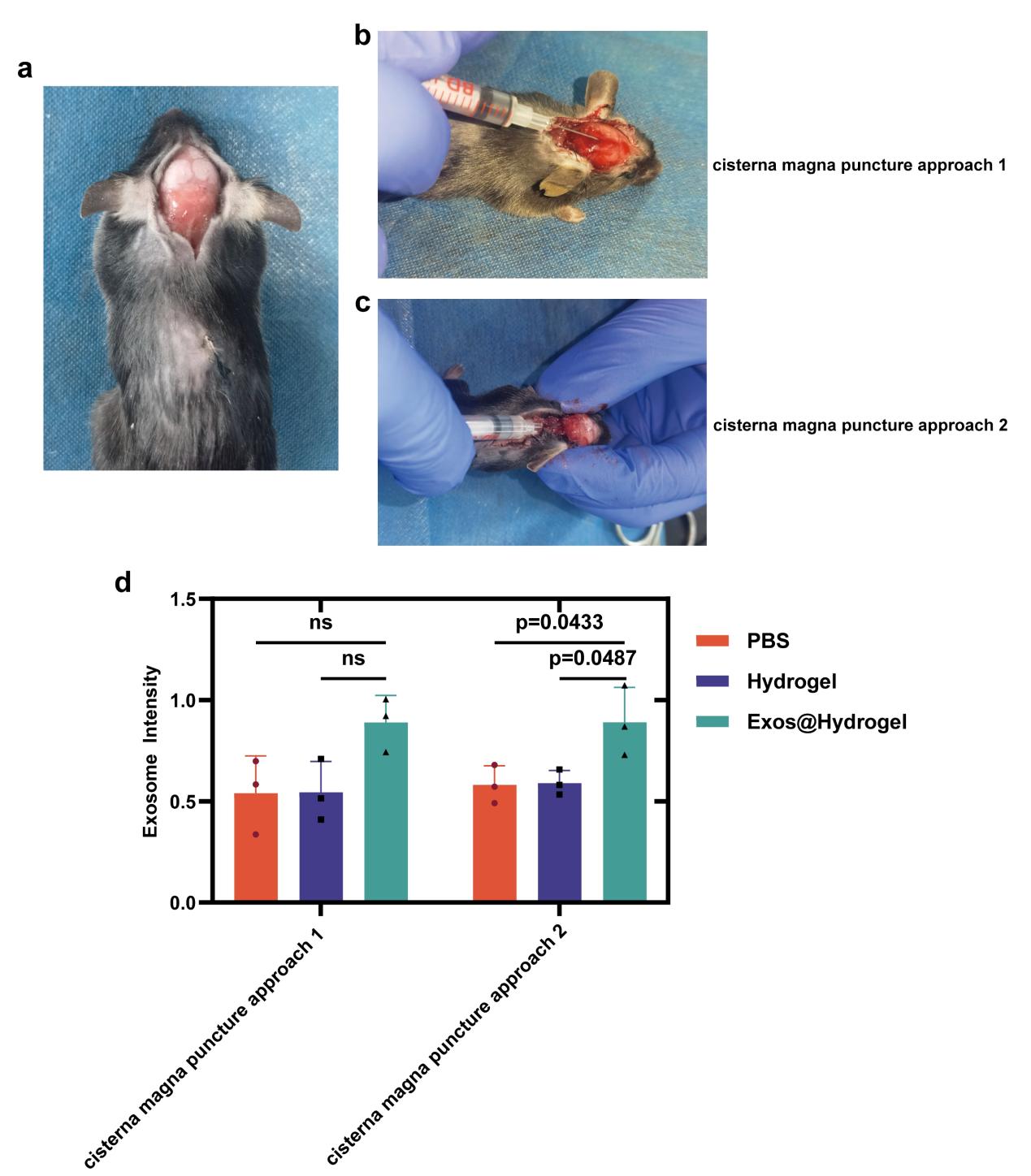


Supporting Figure 27. Comparison of cisterna magna puncture approaches for cerebrospinal fluid (CSF) sampling and exosome quantification.

(a) Dorsal view of the mouse after surgical exposure of the posterior skull for CSF access.

(b, c) Representative images of two different cisterna magna puncture techniques for CSF collection. Approach 1 (b) involves vertical puncture, while approach 2 (c) uses an angled trajectory with gentle neck extension.

(d) Quantification of DiD-labeled exosome fluorescence intensity in CSF collected via each approach, comparing PBS, hydrogel, and Exos@Hydrogel groups. Exos@Hydrogel delivered via approach 2 showed significantly enhanced exosome accumulation in CSF (n = 3).

Data are presented as mean ± SD. Statistical analysis was performed using one-way ANOVA followed by Tukey’s multiple comparisons test. Exact *p-*values are shown in the figure. ns indicates no significant difference.


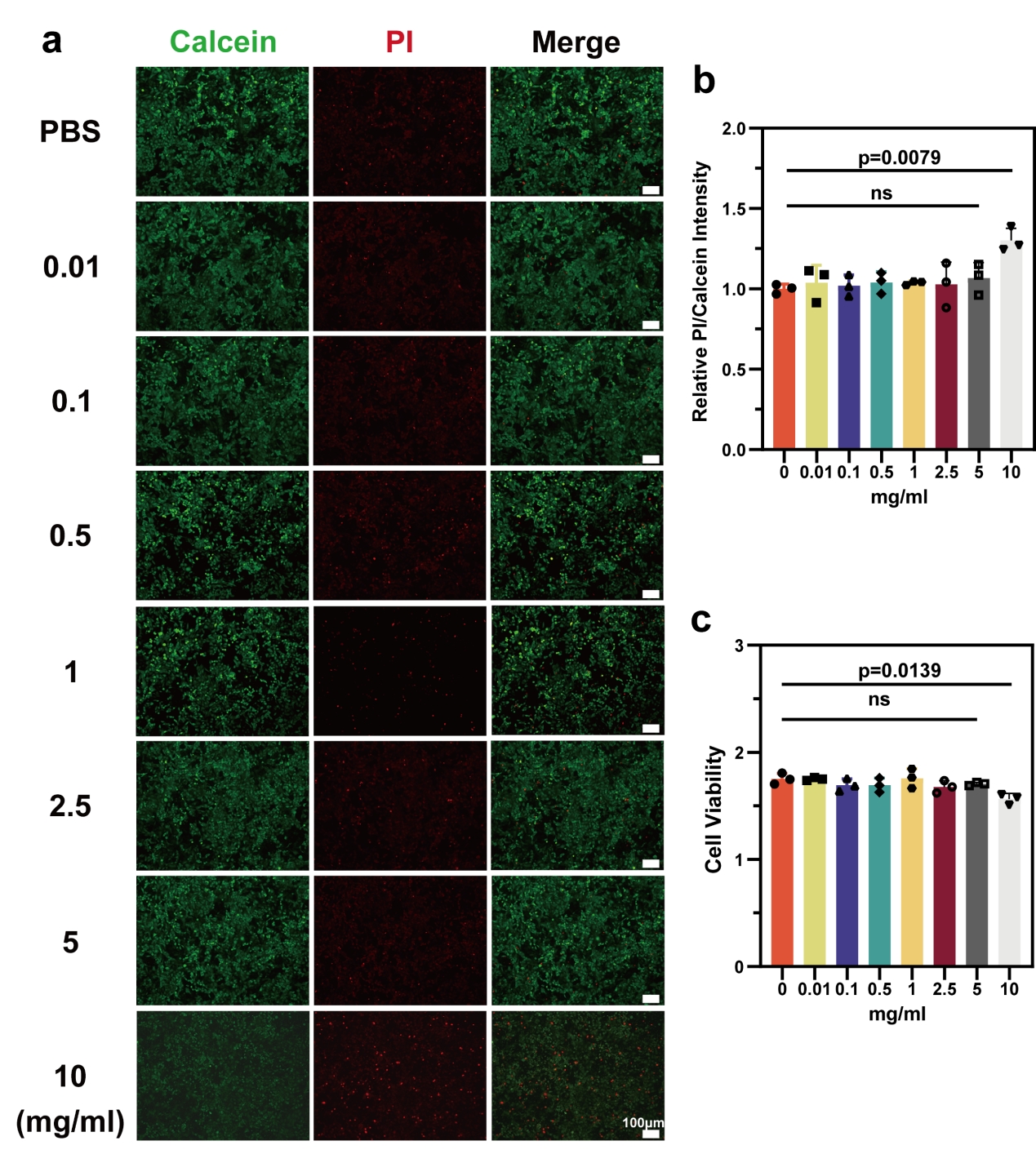


Supporting Figure 28. Evaluation of hydrogel cytocompatibility with A549 cells.

(a) Live/dead staining of A549 cells after 24 h incubation with increasing concentrations (0.01–10 mg/mL) of hydrogel. Live cells are stained green (Calcein-AM), and dead cells are stained red (PI). Scale bar: 100 μm.

(b) Quantification of PI/Calcein fluorescence intensity ratio, reflecting relative cell death. Slight increase in PI signal was observed at 10 mg/mL (n = 3).

(c) CCK-8 assay showing relative cell viability across hydrogel concentrations. No significant cytotoxicity was observed at concentrations ≤5 mg/mL (n = 3).

Data are presented as mean ± SD. Statistical analysis was performed using one-way ANOVA followed by Tukey’s multiple comparisons test. Exact *p-*values are shown in the figure. ns indicates no significant difference.


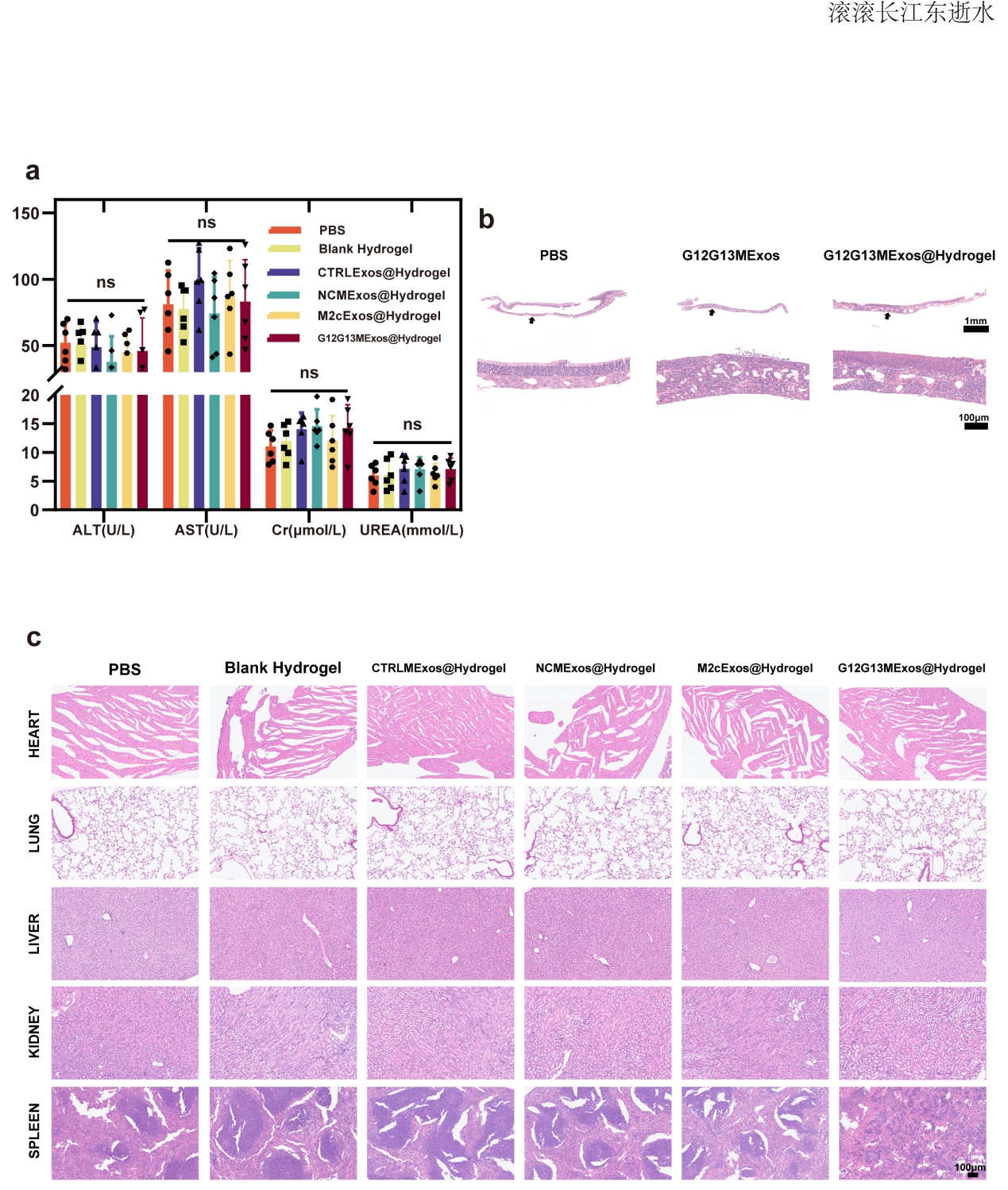


Supporting Figure 29. Comprehensive systemic biosafety evaluation of intranasally delivered exosome-loaded hydrogels.

(a) Serum biochemical parameters including alanine aminotransferase (ALT), aspartate aminotransferase (AST), creatinine (Cr), and urea (UREA) were measured across treatment groups (PBS, blank hydrogel, CTRLExos@Hydrogel, NCMExos@Hydrogel, M2cExos@Hydrogel, and G12G13MExos@Hydrogel). No significant differences were detected, indicating low systemic toxicity (n = 6).

(b) Histological examination of the nasal mucosa by H&E staining showed no apparent pathological changes in mice treated with G12G13MExos or G12G13MExos@Hydrogel compared to PBS, suggesting the intranasal delivery route is well tolerated. Scale bars: 1 mm (upper panels), 100 μm (lower panels) (n = 6).

(c) H&E staining of major organs (heart, lung, liver, kidney, spleen) from different groups revealed no visible tissue damage or inflammation, further confirming the biosafety of hydrogel-encapsulated exosome therapy. Scale bar: 100 μm (n = 3).

Data are presented as mean ± SD. Statistical analysis was performed using one-way ANOVA followed by Tukey’s multiple comparisons test. Exact *p-*values are shown in the figure. ns indicates no significant difference.


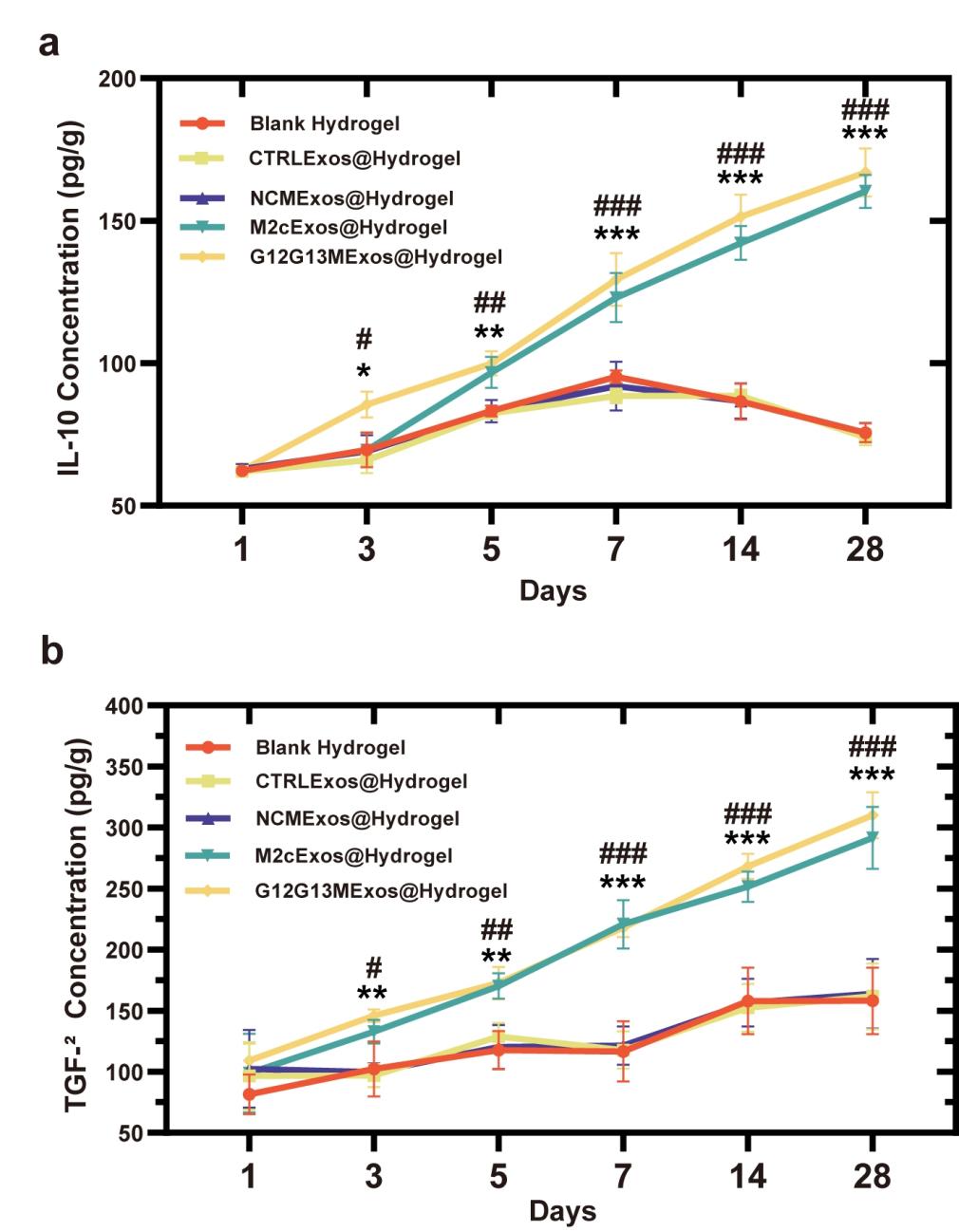


Supporting Figure 30. Time-course analysis of anti-inflammatory cytokine expression in spinal cord tissue after exosome-loaded hydrogel treatments.

(a) Quantification of IL-10 concentrations (pg/g) in spinal cord tissue on days 1, 3, 5, 7, 14, and 28 after treatment with Blank Hydrogel, CTRLExos@Hydrogel, NCMExos@Hydrogel, M2cExos@Hydrogel, and G12G13MExos@Hydrogel. G12G13MExos@Hydrogel and M2cExos@Hydrogel significantly elevated IL-10 levels, particularly from day 5 onward (n = 6).

(b) Quantification of TGF-β concentrations (pg/g) in spinal cord tissue across the same time course. A sustained increase in TGF-β expression was observed in M2cExos@Hydrogel and G12G13MExos@Hydrogel groups, with G12G13MExos@Hydrogel showing the most pronounced effect (n = 6).

Data are expressed as mean ± SD. Statistical analysis was performed using two-way ANOVA followed by Tukey’s multiple comparisons test.**p* < 0.05, ***p* < 0.01, ****p* < 0.001 versus NCMExos@Hydrogel group;#*p* < 0.05, ##*p* < 0.01, ###*p* < 0.001 versus Blank Hydrogel group.


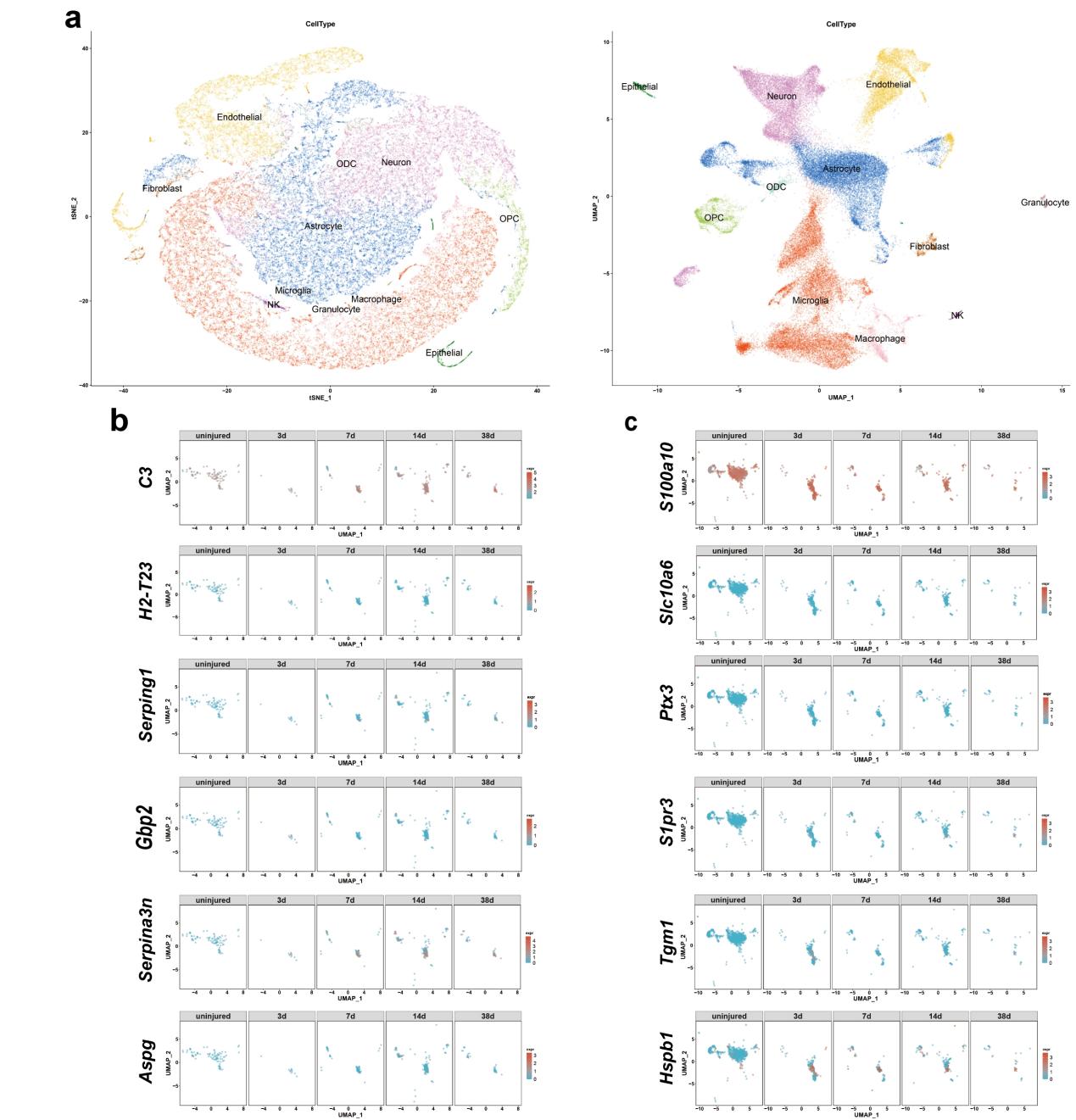


Supporting Figure 31. Single-cell transcriptomic analysis of astrocyte polarization dynamics after SCI.

(a) t-SNE (left) and UMAP (right) plots of major cell types identified by single-cell RNA sequencing from spinal cord tissue. Cell types include astrocytes, neurons, oligodendrocyte progenitor cells (OPCs), oligodendrocytes (ODCs), microglia, macrophages, endothelial cells, fibroblasts, granulocytes, epithelial cells, and natural killer (NK) cells.

(b) Feature plots showing temporal expression patterns of reactive (neurotoxic) astrocyte markers (*C3*, *H2*-*T23*, *Serping1*, *Gbp2*, *Serpina3n*, and *Aspg*) at uninjured, 3, 7, 14, and 28 days post-injury. These genes exhibit peak expression during acute or subacute phases following SCI.

(c) Feature plots of neuroprotective astrocyte markers (*S100a10, Slc1a6, Prx3, S1pr3, Tgm1,* and *Hspb1*), which show a general downregulation post-injury, indicating diminished neuroprotective astrocytic function over time.


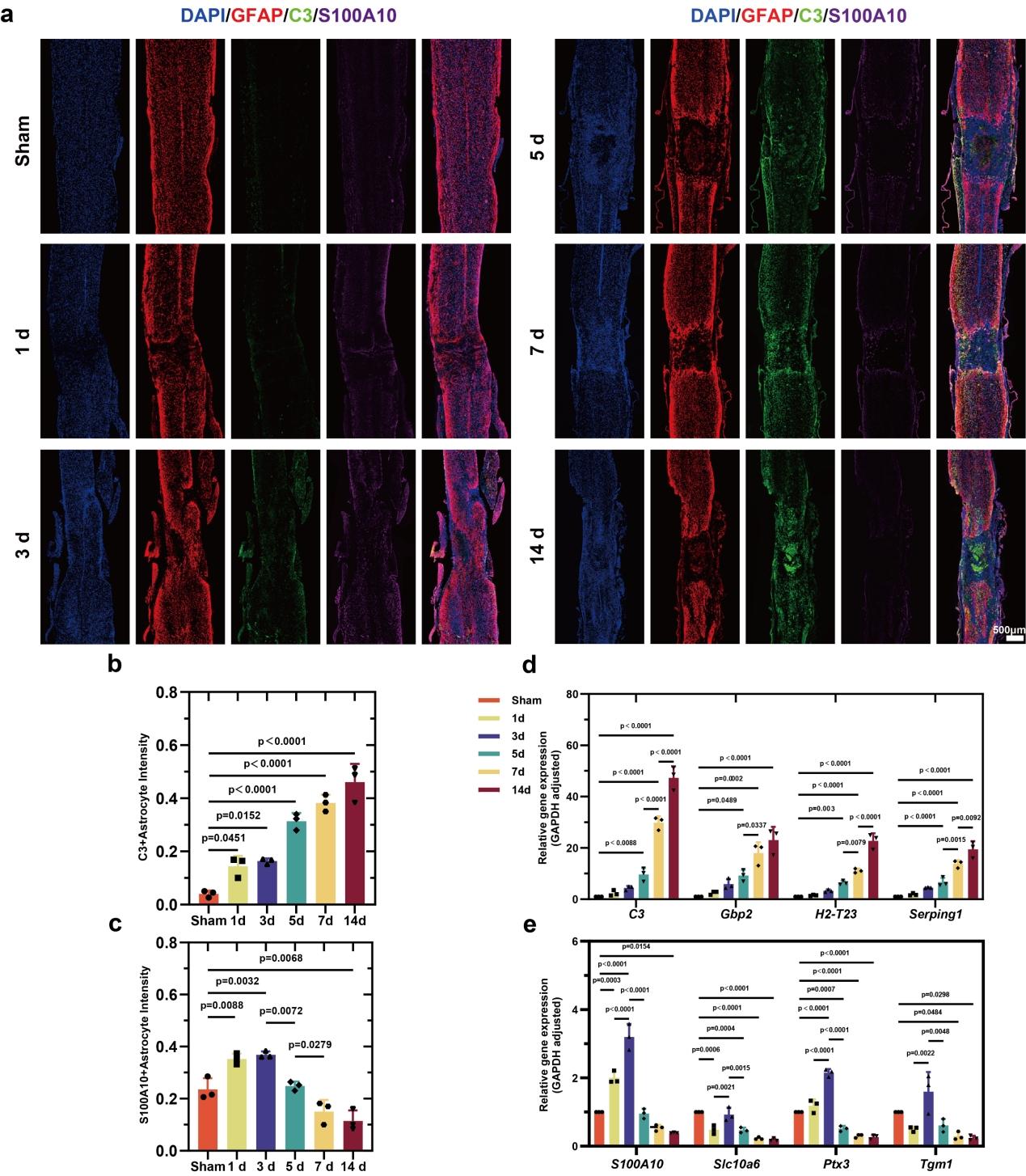


Supporting Figure 32. Temporal dynamics of neurotoxic and neuroprotective astrocyte phenotypes following spinal cord injury SCI.

(a) Representative immunofluorescence images of longitudinal spinal cord sections from Sham and SCI mice at 1, 3, 5, 7, and 14 days post-injury. Sections were stained for DAPI (blue), GFAP (red, astrocyte marker), C3 (green, neurotoxic astrocyte marker), and S100A10 (magenta, neuroprotective astrocyte marker). SCI induced progressive upregulation of C3 expression, whereas S100A10 expression increased transiently during the early phase and declined thereafter. Scale bar: 500 µm.

(b, c) Quantification of fluorescence intensity of C3⁺ and S100A10⁺ astrocytes, showing a time-dependent increase in C3 and a transient rise followed by a sharp decrease in S100A10 levels (n = 3).

(d) mRNA expression of neurotoxic astrocyte markers (C3, Gbp2, H2-T23, Serping1) detected by qPCR at various time points post-SCI, indicating a sustained transcriptional activation of the reactive astrocyte program.

(e) Expression profiles of neuroprotective astrocyte-associated genes (S100A10, Slc1a6, Ptx3, and Tgm1), showing early-phase activation with rapid decline over time (n = 3).

Data are presented as mean ± SD. Statistical analysis was performed using one-way ANOVA followed by Tukey’s multiple comparisons test. Exact *p-*values are shown in the figure. ns indicates no significant difference.


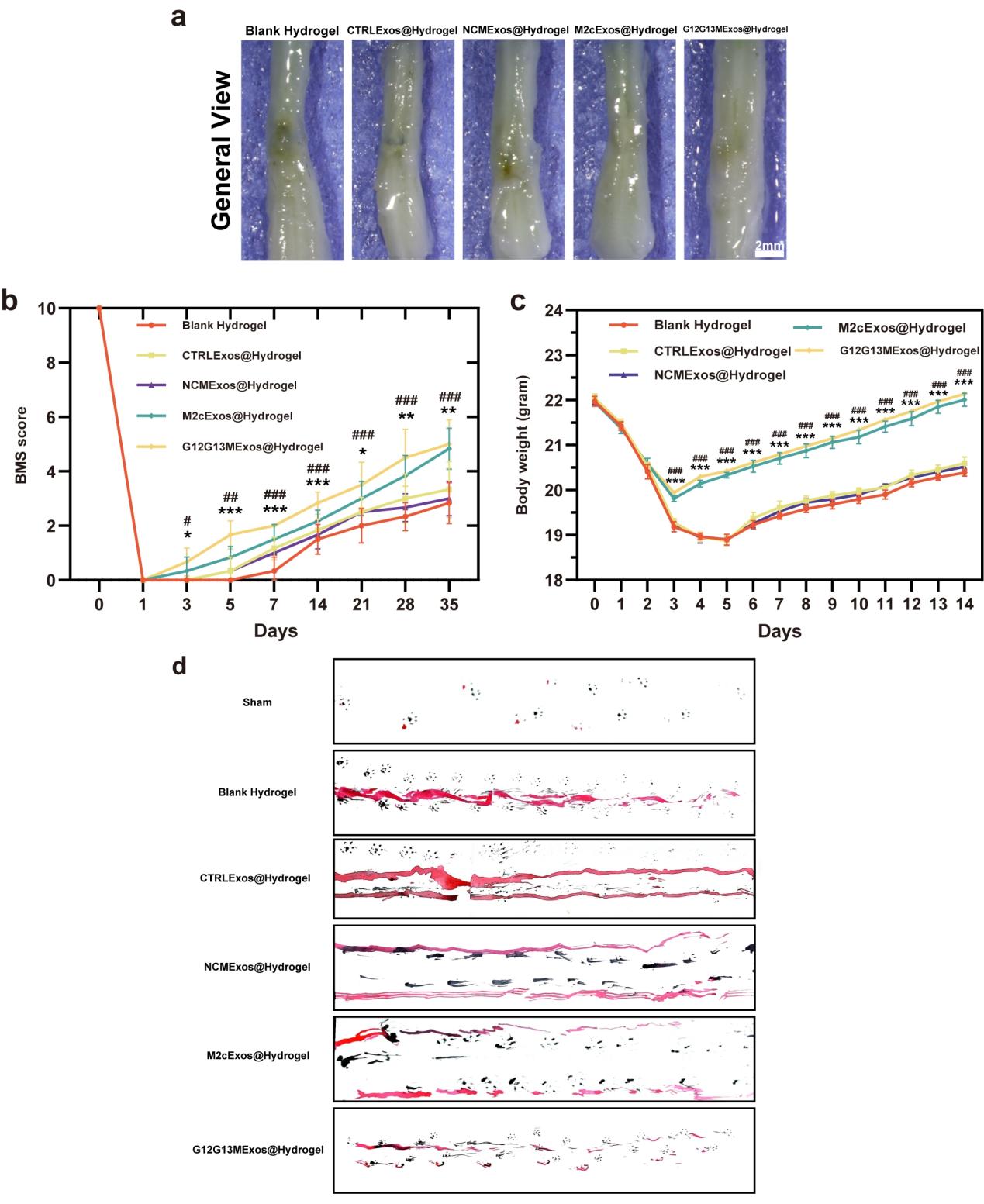


Supporting Figure 33. G12G13MExos@Hydrogel treatment promotes functional recovery and tissue preservation following SCI.

(a) Representative macroscopic images of spinal cords at 35 days post-injury from each treatment group. G12G13MExos@Hydrogel-treated mice exhibited reduced lesion areas and smoother spinal cord surfaces, indicating improved tissue preservation (n = 6). Scale bar: 2 mm.

(b) Time course of Basso Mouse Scale (BMS) locomotor scores up to 35 days post-injury. G12G13MExos@Hydrogel treatment significantly improved motor function recovery compared to control groups (n = 6).

(c) Longitudinal analysis of body weight following SCI. Mice treated with G12G13MExos@Hydrogel showed accelerated weight recovery, suggesting enhanced physiological status post-injury (n = 6).

(d) Footprint analysis from open-field locomotor assay on day 35 post-injury. G12G13MExos@Hydrogel group showed more regular, symmetric gait patterns than control groups, consistent with improved motor coordination (n = 6).

Data are presented as mean ± SD. Statistical analysis was conducted using repeated-measures two-way ANOVA followed by Tukey’s multiple comparisons test. **p* < 0.05, ***p* < 0.01, ****p* < 0.001 indicate comparisons between G12G13MExos@Hydrogel group and NCMExos@Hydrogel group; #*p* < 0.05, ##*p* < 0.01, ###*p* < 0.001 indicate comparisons between G12G13MExos@Hydrogel group and Blank Hydrogel group.


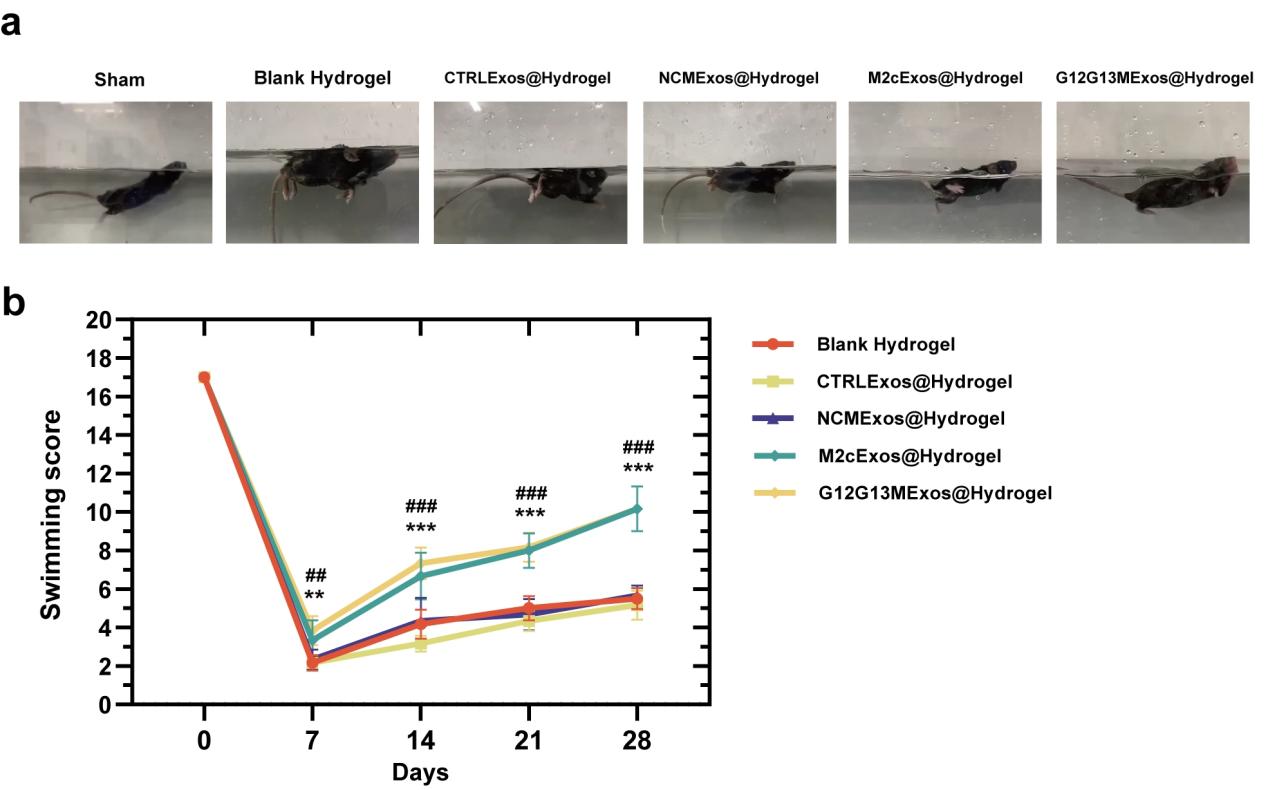


Supporting Figure 34. G12G13MExos@Hydrogel treatment enhances hindlimb coordination in the swimming test following SCI.

(a) Representative images of C57BL/6 mice performing the swimming test on day 28 post-injury. Mice treated with G12G13MExos@Hydrogel displayed more effective hindlimb propulsion, better body alignment, and more coordinated swimming postures compared to other groups.

(b) Time-course analysis of swimming scores recorded on days 0, 7, 14, 21, and 28 post-injury. G12G13MExos@Hydrogel and M2cExos@Hydrogel groups exhibited significantly higher swimming scores compared to control groups at multiple time points, indicating improved locomotor coordination (n = 6).

Data are presented as mean ± SD. Statistical analysis was performed using repeated-measures two-way ANOVA followed by Tukey’s multiple comparisons test. **p* < 0.05, ***p* < 0.01, ****p* < 0.001 versus NCMExos@Hydrogel group; #*p* < 0.05, ##*p* < 0.01, ###*p* < 0.001 versus Blank Hydrogel group.


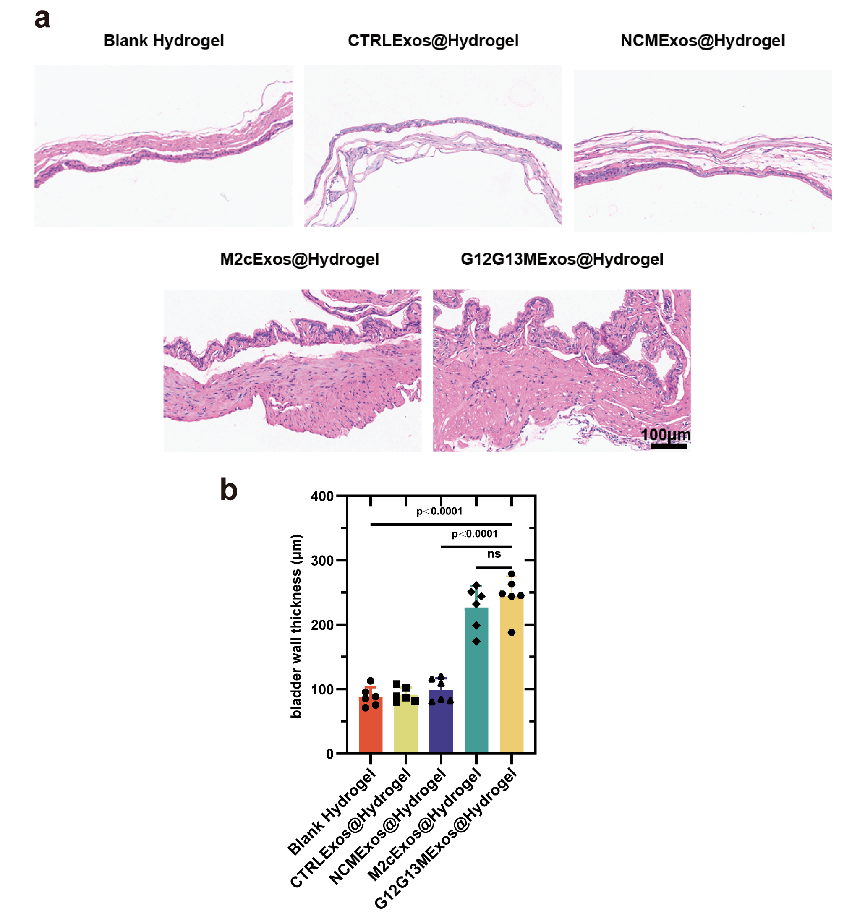


Supporting Figure 35. G12G13MExos@Hydrogel alleviates bladder wall thickening after SCI.

(a) Representative H&E-stained images of bladder tissue sections at 35 days post-SCI from mice treated with Blank Hydrogel, CTRLExos@Hydrogel, NCMExos@Hydrogel, M2cExos@Hydrogel, or G12G13MExos@Hydrogel. Marked bladder wall thickening is observed in the M2cExos and G12G13MExos groups. Scale bar: 100 µm.

(b) Quantification of bladder wall thickness. G12G13MExos@Hydrogel significantly increased bladder wall thickness compared to CTRL and NCM groups, suggesting potential involvement in bladder remodeling (n = 6).

Data are presented as mean ± SD. Statistical analysis was performed using one-way ANOVA followed by Tukey’s multiple comparisons test. Exact *p-*values are shown in the figure. ns indicates no significant difference.


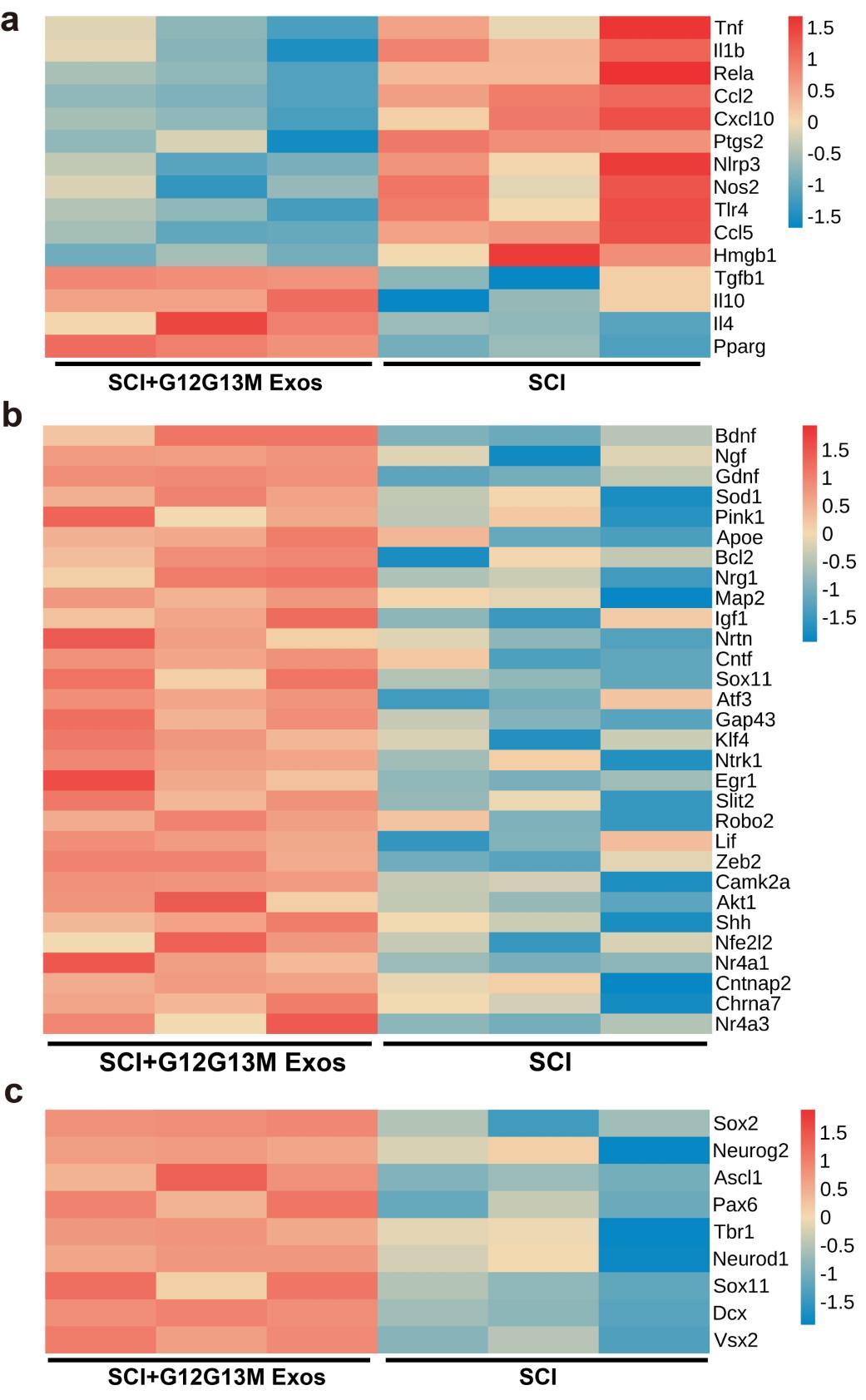


Supporting Figure 36. Transcriptomic profiling reveals G12G13MExos-mediated regulation of inflammation, neuroprotection, and neurogenesis following SCI.
(a) Heatmap showing the expression of inflammation-associated genes in spinal cord tissue from SCI and SCI+G12G13MExos-treated mice. G12G13MExos downregulated pro-inflammatory genes (e.g., *Tnf*, *Il1b*, *Rela*, *Nos2*) while upregulating anti-inflammatory markers (e.g., *Tgfb1*, *Il10*, *Il4*).
(b) Heatmap depicting neuroprotective and synaptic plasticity-related genes. G12G13MExos-treated tissue exhibited higher expression of neurotrophic factors (*Bdnf*, *Ngf*, *Gdnf*), oxidative stress regulators (*Sod1*, *Pink1*), and regeneration-associated genes (*Gap43*, *Ntrk1*, *Camk2a*, *Akt1*).
(c) Heatmap illustrating the expression of neurogenic transcription factors. Key markers including *Sox2*, *Neurog2*, *Ascl1*, and *Tbr1* were significantly upregulated in the G12G13MExos group, suggesting enhanced neural progenitor activity and neuronal differentiation potential.


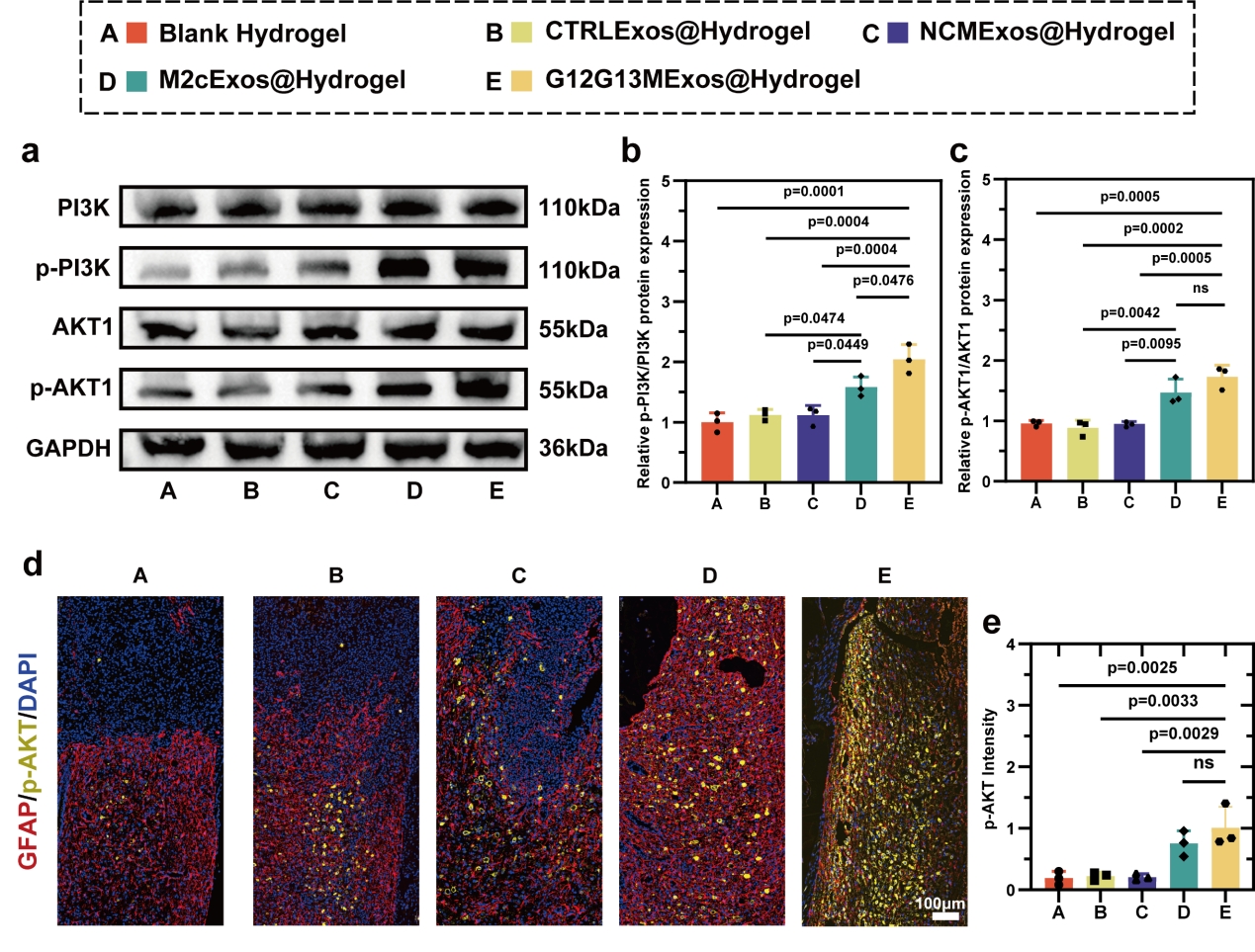


Supporting Figure 37. G12G13MExos activate the PI3K-AKT signaling pathway in spinal cord tissue after SCI.

(a) Western blot analysis of total PI3K, phosphorylated PI3K (p*-*PI3K), total AKT1, and phosphorylated AKT1 (p*-*AKT1) in spinal cord tissue at day 7 post-SCI across different treatment groups.

(b, c) Quantification of the relative expression levels of p-PI3K/PI3K (b) and p*-*AKT1/AKT1 (c), showing significant upregulation of PI3K-AKT pathway activation in the G12G13MExos@Hydrogel group compared to other groups (n = 3).

(d) Immunofluorescence staining for GFAP (red), p*-*AKT (yellow), and DAPI (blue) in spinal cord sections. G12G13MExos@Hydrogel-treated mice exhibited markedly increased p*-*AKT expression, particularly in GFAP⁺ astrocytes. Scale bar: 100 µm.

(e) Quantification of p*-*AKT fluorescence intensity in spinal cord tissue across groups confirms enhanced PI3K-AKT pathway activation in response to G12G13MExos (n = 3).

Data are presented as mean ± SD. Statistical analysis was performed using one-way ANOVA followed by Tukey’s multiple comparisons test. Exact *p-*values are shown in the figure. ns indicates no significant difference.


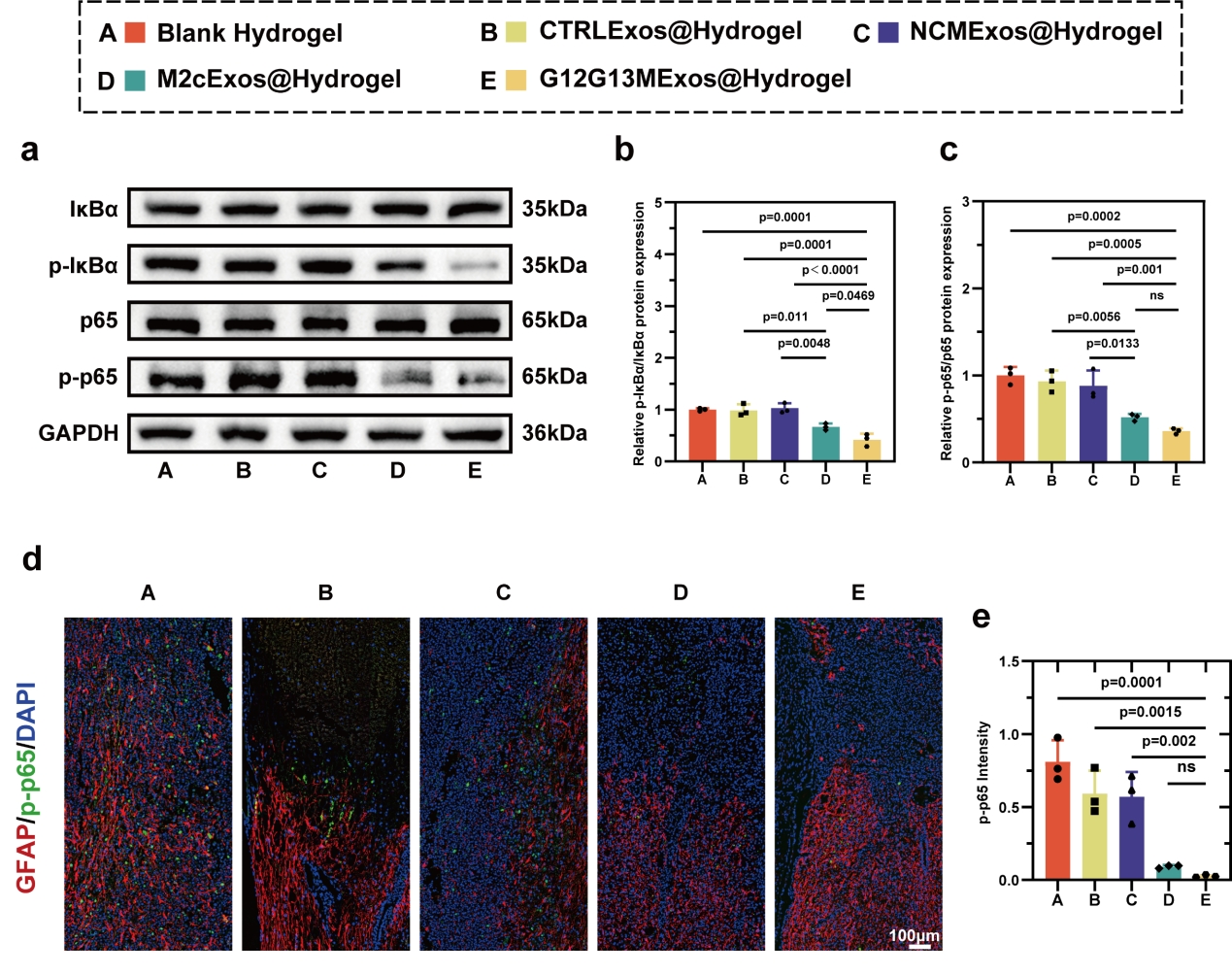


Supporting Figure 38. G12G13MExos inhibit NF-κB signaling activation in spinal cord tissue after SCI.

(a) Western blot analysis of total IκBα, phosphorylated IκBα (p*-*IκBα), total NF-κB p65, and phosphorylated p65 (p*-*p65) in spinal cord tissue collected on day 7 post-SCI across different treatment groups.

(b, c) Quantification of relative expression levels of p*-*IκBα/IκBα (b) and p*-*p65/p65 (c), demonstrating significant suppression of NF-κB pathway activation in the G12G13MExos@Hydrogel group compared to controls (n = 3).

(d) Immunofluorescence staining of spinal cord sections for GFAP (red), p-p65 (green), and DAPI (blue), showing reduced nuclear p-p65 localization in G12G13MExos@Hydrogel-treated mice. Scale bar: 100 µm.

(e) Quantification of p*-*p65 fluorescence intensity in spinal cord sections, confirming reduced NF-κB activation following G12G13MExos treatment (n = 3).

Data are presented as mean ± SD. Statistical analysis was performed using one-way ANOVA followed by Tukey’s multiple comparisons test. Exact *p-*values are shown in the figure. ns indicates no significant difference.

**Materials and Methods**

**Table S1.** Reagents

| Name | Supplier name | Catalogue number | Country |
| --- | --- | --- | --- |
| DMEM High glucose medium | Thermo Gibco | 11965092 | MA, USA |
| DMEM/F12 medium | Thermo Gibco | 11320033 | MA, USA |
| DMEM medium | Thermo Gibco | 11966025 | MA, USA |
| NEUROBASAL-A | Thermo Gibco | 10888022 | MA, USA |
| KnockOut DMEM/F-12 | Thermo Gibco | 12660012 | MA, USA |
| DMEM/F-12, no glutamine | Thermo Gibco | 21331020 | MA, USA |
| Fetal calf serum | Thermo Gibco | 10091155 | MA, USA |
| Fetal Bovine Serum exosome-depleted | Thermo Gibco | A2720801 | MA, USA |
| GlutaMAX-I | Thermo Gibco | A1286001 | MA, USA |
| StemPro Neural Supplement | Thermo Gibco | A1050801 | MA, USA |
| N-2 Supplement | Thermo Gibco | 17502048 | MA, USA |
| 0.25% Trypsin | Thermo Gibco | 15050065 | MA, USA |
| Poly-D-Lysine | Thermo Gibco | A3890401 | MA, USA |
| Triton X-100 | Thermo Gibco | HFH10 | MA, USA |
| RIPA buffer | Thermo Scientific | 89900 | MA, USA |
| Lipofectamine RNAiMAX Transfection Reagent | Thermo Scientific | 13778150 | MA, USA |
| Trizol | Thermo Invitrogen | 15596026CN | MA, USA |
| DNaseI | Thermo Invitrogen | 18068015 | MA, USA |
| LPS | Sigma-Aldrich | L5293 | MO, USA |
| Bovine serum albumin (BSA) | Sigma-Aldrich | A1933 | MO, USA |
| DAPI solution | Sigma-Aldrich | MBD0015 | MO, USA |
| Puromycin | MedChemExpress | HY-K1057 | NJ, USA |
| SB-431542 | MedChemExpress | HY-10431 | NJ, USA |
| L-Glutamic acid | MedChemExpress | HY-14608 | NJ, USA |
| glacial acetic acid | Aladdin | A116166 | Shanghai, China |
| Chitosan | Macklin | C875439 | Shanghai, China |
| β-Glycerol phosphate disodium salt | Macklin | G806967 | Shanghai, China |
| polyethylene glycol 400 | Macklin | P815616 | Shanghai, China |
| Chloroform-d | Macklin | C805077 | Shanghai, China |
| Isopropyl alcohol | Macklin | I811925 | Shanghai, China |
| Ethanol anhydrous | Macklin | E809056 | Shanghai, China |
| Papain | Solarbio | G8430 | Beijing, China |
| DiI | Beyotime | C1036 | Beijing, China |
| DiD | Beyotime | C1039 | Beijing, China |
| DiO | Beyotime | C1038 | Beijing, China |
| phalloidin-FITC | Beyotime | C1033 | Beijing, China |
| Penicillin - streptomycin | seven biotech | SC118-01 | Beijing, China |
| paraformaldehyde | Servicebio | G1101 | Wuhan,China |

**Table S2.** Antibodies

| Name | Supplier name | Catalogue number | Country |
| --- | --- | --- | --- |
| Anti-TGF-β1 antibody | Abcam | ab315254 | Cambridge, UK |
| Anti-iNOS antibody | Abcam | ab283655 | Cambridge, UK |
| Anti-CD86 antibody | Abcam | ab112490 | Cambridge, UK |
| Anti-CD163 antibody | Abcam | ab182422 | Cambridge, UK |
| Anti-Liver Arginase antibody | Abcam | ab315110 | Cambridge, UK |
| Anti-F4/80 antibody | Abcam | ab6640 | Cambridge, UK |
| Anti-CD9 antibody | Abcam | ab307085 | Cambridge, UK |
| Anti-CD63 antibody | Abcam | ab217345 | Cambridge, UK |
| Anti-CD81 antibody | Abcam | ab109201 | Cambridge, UK |
| Anti-Calnexin antibody | Abcam | ab22595 | Cambridge, UK |
| Anti-HSP70 antibody | Abcam | ab5439 | Cambridge, UK |
| Anti-Iba1 antibody | Abcam | ab178846 | Cambridge, UK |
| Anti-CD68 antibody | Abcam | ab125212 | Cambridge, UK |
| Anti-GAPDH antibody | Abcam | ab181602 | Cambridge, UK |
| Anti-MAP2 antibody | Abcam | ab300645 | Cambridge, UK |
| Anti-NeuN antibody | Abcam | ab177487 | Cambridge, UK |
| Anti-GFAP antibody | Abcam | ab68428 | Cambridge, UK |
| Anti-C3 antibody | Abcam | ab200999 | Cambridge, UK |
| Anti-Nestin antibody | Abcam | ab313787 | Cambridge, UK |
| Anti-SOX2 antibody | Abcam | ab97959 | Cambridge, UK |
| Anti-Caspase-3 antibody | Abcam | ab13585 | Cambridge, UK |
| Anti-BCL2 antibody | Abcam | ab182858 | Cambridge, UK |
| Anti-BAX antibody | Abcam | ab32503 | Cambridge, UK |
| Anti-EAAT1 antibody | Abcam | ab181036 | Cambridge, UK |
| Anti-EAAT2 antibody | Abcam | ab205248 | Cambridge, UK |
| Anti-Synaptophysin antibody | Abcam | ab32127 | Cambridge, UK |
| Anti-Synapsin I antibody | Abcam | ab254349 | Cambridge, UK |
| Anti-GAPDH antibody | Abcam | ab8245 | Cambridge, UK |
| Anti-beta Actin antibody | Abcam | ab8226 | Cambridge, UK |
| Goat Anti-Rabbit IgG H&L (Alexa Fluor^®^ 488) | Abcam | ab150077 | Cambridge, UK |
| Goat Anti-Mouse IgG H&L (Alexa Fluor^®^ 488) | Abcam | ab150113 | Cambridge, UK |
| Goat Anti-Rabbit IgG H&L (Alexa Fluor^®^ 594) | Abcam | ab150080 | Cambridge, UK |
| Goat Anti-Mouse IgG H&L (Alexa Fluor^®^ 594) | Abcam | ab150116 | Cambridge, UK |
| Goat Anti-Rabbit IgG H&L (Alexa Fluor^®^ 647) | Abcam | ab150079 | Cambridge, UK |
| Goat Anti-Mouse IgG H&L (Alexa Fluor^®^ 647) | Abcam | ab150115 | Cambridge, UK |
| Goat Anti-Rabbit IgG H&L (Alexa Fluor^®^ 555) | Abcam | ab150078 | Cambridge, UK |
| S100A10 Monoclonal antibody | Thermo Scientific | MA5-24769 | MA, USA |
| β3-Tubulin (TU-20) Mouse mAb | Cell Signaling Technology | #4466 | Massachusetts, USA |
| Neurofilament-H (E7Z7G) Rabbit mAb | Cell Signaling Technology | #30564 | Massachusetts, USA |
| SMAD3 (C67H9) Rabbit mAb | Cell Signaling Technology | #9523 | Massachusetts, USA |
| Phospho-SMAD3 (Ser423/425) (C25A9) Rabbit mAb | Cell Signaling Technology | #9520 | Massachusetts, USA |
| IκBα (44D4) Rabbit mAb | Cell Signaling Technology | #4812 | Massachusetts, USA |
| Phospho-IκBα (Ser32) (14D4) Rabbit mAb | Cell Signaling Technology | #2859 | Massachusetts, USA |
| NF-κB p65 (D14E12) Rabbit mAb | Cell Signaling Technology | #8242 | Massachusetts, USA |
| Phospho-NF-κB p65 (Ser536) (93H1) Rabbit mAb | Cell Signaling Technology | #3033 | Massachusetts, USA |
| Akt1 (C73H10) Rabbit mAb | Cell Signaling Technology | #2938 | Massachusetts, USA |
| Phospho-Akt1 (Ser473) (D7F10) XP® Rabbit mAb | Cell Signaling Technology | #9018 | Massachusetts, USA |
| PI3 Kinase p110 Beta Polyclonal antibody | Proteintech | 20584-1-AP | Wuhan,China |
| VSX2 Polyclonal antibody | Proteintech | 25825-1-AP | Wuhan,China |
| Rabbit Anti-phospho-PI3 Kinase p110 beta (Ser1070) antibody | Bioss | bs-6417R | Beijing, China |
| GNA12 Antibody | Affinity | #DF2203 | Jiangsu, China |
| GNA13 Antibody | Affinity | #DF2204 | Jiangsu, China |

**Table S3.** Kits

| Name | Supplier name | Catalogue number | Country |
| --- | --- | --- | --- |
| HE Staining Kit | Solarbio | G1120 | Beijing, China |
| Nissl Staining Kit | Solarbio | G1430 | Beijing, China |
| Mouse Transforming Growth Factor Beta (TGF-β) ELISA Kit | Jianglai | JL13959 | Shanghai, China |
| Mouse Interleukin 10 (IL-10) ELISA Kit | Jianglai | JL20242 | Shanghai, China |
| Mouse Interleukin 1 Beta (IL-1β) ELISA Kit | Jianglai | JL18442 | Shanghai, China |
| Mouse Interleukin 6 (IL-6) ELISA Kit | Jianglai | JL20268 | Shanghai, China |
| Mouse Tumor Necrosis Factor Alpha (TNFα) ELISA Kit | Jianglai | JL10484 | Shanghai, China |
| Mouse Brain Derived Neurotrophic Factor (BDNF) ELISA Kit | Jianglai | JL12317 | Shanghai, China |
| Mouse Glial Cell Line Derived Neurotrophic Factor (GDNF) ELISA Kit | Jianglai | JL10491 | Shanghai, China |
| CCK-8 | Beyotime | C0037 | Shanghai, China |
| EdU staining kit | Beyotime | C0078S | Shanghai, China |
| One Step TUNEL Apoptosis Assay Kit | Beyotime | C1089 | Shanghai, China |
| Annexin V-FITC Apoptosis Detection Kit | Beyotime | C1062 | Shanghai, China |
| [Reactive oxygen species Assay Kit](http://www.njjcbio.com/products.asp?id=819) | JianCheng | [E004-1-1](http://www.njjcbio.com/products.asp?id=819) | Nanjing,China |
| PrimeScriptTM RT reagent Kit with gDNA Eraser (Perfect Real Time) | TaKaRa | RR047A | Shiga, Japan |
| SYBR Premix Ex TaqTM II (Tli RNaseH Plus) | TaKaRa | RR820A | Shiga, Japan |
| Glutamic Acid (Glu) Content Assay Kit | Sangon Biotech | D799586 | Shanghai, China |

**Table S4.** Exogenous Protein

| Name | Supplier name | Catalogue number | Country | Origin |
| --- | --- | --- | --- | --- |
| TGF beta 1/TGFB1 protein, Mouse/Rat | MedChemExpress | HY-P70648 | NJ, USA | HEK293 Cells |
| MCP*-*1/CCL2 protein, Mouse | MedChemExpress | HY-P7764 | NJ, USA | E.coli |
| EGF Protein, Mouse | MedChemExpress | HY-P70590 | NJ, USA | E.coli |
| IFN-gamma Protein, Mouse | MedChemExpress | HY-P7071 | NJ, USA | E.coli |
| FGF-2 protein, Mouse | MedChemExpress | HY-P7066 | NJ, USA | E.coli |

**Table S5.** Primer sequences for qRT-PCR analysis

| Gene | Forward primer | Reverse primer |
| --- | --- | --- |
| Gna12 | 5′-CCAAGGTTCAGGTGTACGCA-3′ | 5′-TGAGCTTAGCAGCATCGCAT-3′ |
| Gna13 | 5’-CACCAAAGGCATCCATGAGT-3’ | 5’-GACGTTGCTGAAAACCCGAT-3’ |
| Tgfb1 | 5’-CTCCCGTGGCTTCTAGTGC-3’ | 5’-GCCTTAGTTTGGACAGGATCTG-3’ |
| Arg1 | 5’-CCAAGCCAAAGCCCATAGAG-3’ | 5’-TCCTCGAGGCTGTCCCTTAG-3’ |
| Cd163 | 5’-TGTAGTTCATCATCTTCGGTCC-3’ | 5’-CACCTACCAAGCGGAGTTGAC-3’ |
| Nos2 | 5’-CTGCCAGGGTCACAACTTTACA-3’ | 5’-AACAGCTCAGTCCCTTCACC-3’ |
| Cd86 | 5’-TAGGGATAACCAGGCTCTAC-3’ | 5’-CGTGGGTGTCTTTTGCTGTA-3’ |
| C3 | 5’-CGCAACGAACAGGTGGAGATCA-3’ | 5’-CTGGAAGTAGCGATTCTTGGCG-3’ |
| Gbp2 | 5’-AGATGCCCACAGAAACCCTCCA-3’ | 5’-AAGGCATCTCGCTTGGCTACCA-3’ |
| H2-T23 | 5’- AGTATTGGGAGCGGGAGACTT-3’ | 5’- AGCACCTCAGGGTGACTTCAT-3’ |
| Serping1 | 5’-TTGCCTGTGTCCACCAAGCACT-3’ | 5’-GCTGCTTCCATACAGGCTCTGA-3’ |
| S100a10 | 5’-GACAAAGGAGGACCTGAGAGTG-3’ | 5’-CTCTGGAAGCCCACTTTGCCAT-3’ |
| Slc10a6 | 5’-TCCTGGACTCTGACACAGAACC-3’ | 5’-CACATAGACGCCAGAAGCCACA-3’ |
| Ptx3 | 5’-CGAAATAGACAATGGACTTCATCC-3’ | 5’-CATCTGCGAGTTCTCCAGCATG-3’ |
| Tgm1 | 5’-ATCTGCCCTCAGGCTTTGATGG-3’ | 5’-CGTTCTTGACGGACTCCACAGA-3’ |
| Thbs1 | 5’-GGTAGCTGGAAATGTGGTGCGT-3’ | 5’-GCACCGATGTTCTCCGTTGTGA-3’ |
| Gpc6 | 5’-AGAGGTTGCCAACCGAGTTTCC-3’ | 5’-TGCAAGGTCTCACAGTGGGCAA-3’ |
| Megf10 | 5’-CGACAGATCCTGCCAGTGTTAC-3’ | 5’-CAAAAGGCTCCGTTGTGGCAGT-3’ |
| Mertk | 5’-ATCATCCTCGGCTGCTTCTGTG-3’ | 5’-ACGACCAGTTGGGAATCCTCCT-3’ |
| Gapdh | 5’-AATGTGTCCGTCGTGGATCTGA-3’ | 5’-AGTGTAGCCCAAGATGCCCTTC-3’ |

**Table S6.** Sequences for si-RNA

| Gene | Sense primer | Antisense primer |
| --- | --- | --- |
| MmSmad3-siRNA-886 | 5'-CCAGUGACCACCAGAUGAATT-3' | 5'-UUCAUCUGGUGGUCACUGGTT-3' |
| MmSmad3-siRNA-1684 | 5'-GAACACCGAUUCCACUCAATT-3' | 5'-UUGAGUGGAAUCGGUGUUCTT-3' |
| MmSmad3-siRNA-4135 | 5'-ACAUCUGACUUCAUUCUUATT-3' | 5'-UUAAGAAUGAAGUCAGAUGTT-3' |
| MmPik3ca-siRNA-1038 | 5'-GCACAUCUACAACAAGUUATT-3' | 5'-UAACUUGUUGUAGAUGUGCTT-3' |
| MmPik3ca-siRNA-1314 | 5'-GUACAUAAGAAGCUGUAUATT-3' | 5'-UAUACAGCUUCUUAUGUACTT-3' |
| MmPik3ca-siRNA-2601 | 5'-GCACCUGAACAGACAAGUATT-3' | 5'-UACUUGUCUGUUCAGGUGCTT-3' |
| MmRelA-siRNA-953 | 5'-AAGAAGACAUUGAGGUGUATT-3' | 5'-UACACCUCAAUGUCUUCUUTT-3' |
| MmRelA-siRNA-1202 | 5'-GGACCUAUGAGACCUUCAATT-3' | 5'-UUGAAGGUCUCAUAGGUCCTT-3' |
| MmRelA-siRNA-1863 | 5'-UGGUCUCUCAGGAGAUGAATT-3' | 5'-UUCAUCUCCUGAGAGACCATT-3' |

## 1. Experimental Materials and Reagents

### 1.1 Animals and Cell Lines

Eight-week-old female C57BL/6 mice weighing 22 ± 1 g were purchased from the Animal Experimental Center of the Second Affiliated Hospital of Harbin Medical University. The mice were housed under standard laboratory conditions (temperature: 20–22°C, humidity: 40–70%, 12-hour light/dark cycle). This study was approved by the Animal Care and Use Committee of the First Affiliated Hospital of Harbin Medical University, Harbin, Heilongjiang, China (Approval No.: 2020052).

The RAW 264.7 murine macrophage cell line was purchased from iCell Bioscience Inc. (Cat. No.: iCell-m047). The BV2 murine microglial cell line was obtained from iCell Bioscience Inc. (Cat. No.: iCell-m011). All cell lines were authenticated using short tandem repeat (STR) profiling and confirmed to be free of mycoplasma contamination. Primary astrocytes, neurons, and neural stem cells were isolated from C57BL/6 mice.

## **1.2 Key Reagents and Antibodies** Refer to Tables S1–S4 for detailed information.

## 2. Routine Experimental Methods

### 2.1 Cell Isolation and Culture

RAW 264.7 and BV2 cells were cultured in high-glucose DMEM (Thermo Gibco) supplemented with 10% fetal bovine serum (FBS), 100 U/mL penicillin, and 0.1 mg/mL streptomycin under standard conditions (37°C, 5% CO₂, humidified atmosphere).

Primary astrocytes, neurons, and neural stem cells were isolated from neonatal C57BL/6 mice. Under sterile conditions, the cerebral cortex was dissected to remove the meninges and blood vessels and finely minced. Tissue digestion was performed using 0.25% papain and 0.01% DNase I at 37°C for 20 minutes, with gentle pipetting every 5 minutes. The digestion was terminated by adding DMEM/F12 supplemented with 10% FBS, followed by centrifugation to remove the supernatant. The cells were resuspended in the appropriate medium to prepare a single-cell suspension.

Astrocytes were plated into culture flasks coated with 0.1 mg/mL poly-L-lysine at a density of 1.2×10⁶ cells/mL and cultured in DMEM/F12 medium containing 10% FBS at 37°C in a 5% CO₂ incubator for 8–9 days. High-purity astrocytes were obtained by differential adhesion to remove microglia and oligodendrocytes.

Neurons were seeded onto poly-L-lysine-coated plates and cultured in Neurobasal medium containing 2% B-27 supplement and 2 mM GlutaMAX-I. The medium was replaced with fresh medium 24 hours after plating and subsequently changed every 2–3 days to maintain cell growth and viability.

NSCs were plated in uncoated culture flasks and cultured in KnockOut D-MEM/F-12 supplemented with 20 ng/mL bFGF, 20 ng/mL EGF, 2% B-27, and 2 mM GlutaMAX at 37°C in a 5% CO₂ incubator. Neurospheres were passaged regularly to maintain stem cell properties.

### 2.2 siRNA Transfection

Cell transfection was performed using the RNAiMAX Transfection Reagent to enhance siRNA transfection efficiency. Under sterile conditions, siRNA (10 µM) and the transfection reagent were diluted in serum-free medium and mixed at a 1:1 ratio to form RNA-lipid complexes. The mixture was incubated at room temperature for 5 minutes.

The RNA-lipid complexes were added to the cell culture medium when cells reached 60–80% confluence. During the transfection process, the medium was modified to minimize interference from antibiotics and fetal bovine serum. After 6 hours of transfection, the complexes were removed, and the cells were cultured in complete medium containing 10% FBS for further incubation.

### 2.3 Western Blot Analysis

Western blot analysis was used to detect specific protein expression levels. Total protein was extracted from cells or tissues using RIPA lysis buffer, followed by lysis on ice for 30 minutes. The lysates were centrifuged at 12,000 rpm at 4°C for 20 minutes, and the supernatant was collected. Protein concentration was quantified using the BCA method.

Equal amounts of protein were mixed with loading buffer and denatured at 95°C for 5 minutes. The proteins were separated on 7.5%-15% SDS-polyacrylamide gels and transferred onto PVDF membranes using the wet transfer method. The membranes were blocked with 5% non-fat milk for 1 hour and incubated with the primary antibody at 4°C overnight. The membranes were washed three times with TBST and then incubated with HRP*-*conjugated secondary antibodies for 1 hour.

After washing, the membranes were treated with chemiluminescent substrate, and images were captured using a chemiluminescence imaging system (Tanon 5100, Tanon, China). Band intensities were quantified using image analysis software and normalized to the corresponding internal control to determine the relative expression levels of target proteins.

### 2.4 Real-Time Quantitative PCR (qPCR)

Real-time quantitative PCR (qPCR) was performed to quantify the expression levels of specific genes. Total RNA was extracted using the Trizol method, and genomic DNA contamination was removed. The purified RNA was then reverse-transcribed into cDNA.

The qPCR reactions were carried out using SYBR Green as the detection dye on a LightCycler® 96 system (Roche, Switzerland). The amplification conditions were as follows: initial denaturation at 95°C for 30 seconds, followed by 40 cycles of 95°C for 5 seconds and 60°C for 30 seconds. GAPDH was used as the internal control, and the primer sequences for qPCR amplification are provided in Supplementary Table S5.

The relative gene expression levels were calculated using the 2^-ΔΔCt method and normalized for experimental analysis requirements.

### 2.5 Immunofluorescence (IF)

Immunofluorescence (IF) was performed on both cell and tissue samples, incorporating nuclear staining. Samples were fixed with 4% paraformaldehyde for 15 minutes and washed three times with PBS. Permeabilization was carried out with 0.2% Triton X-100 for 10 minutes, followed by blocking with PBS containing 10% normal goat serum for 1 hour at room temperature.

Primary antibodies were incubated with the samples overnight at 4°C. The next day, the samples were washed three times with PBS and incubated with fluorophore-conjugated secondary antibodies for 1 hour at room temperature in the dark. After three additional PBS washes, nuclei were stained with DAPI for 5 minutes at room temperature in the dark. The samples were washed twice with PBS and mounted using anti-fade mounting medium. For cell samples, slides were prepared by transferring the specimens to glass coverslips.

Fluorescence images were captured using an LSM 980 confocal laser scanning microscope (Zeiss, Germany) under consistent settings for laser intensity, gain, and exposure time. Fluorescence intensity was quantified using ImageJ software (NIH, USA), with at least three random fields of view analyzed. Results were presented as mean ± standard deviation (SD), and statistical significance was assessed (P < 0.05).

### 2.6 Flow Cytometry (FACS)

For flow cytometry (FACS) analysis, cells were washed with PBS and resuspended in PBS containing 2% bovine serum albumin (BSA) to prepare a single-cell suspension. Samples were incubated with primary antibodies at 4°C in the dark for 30 minutes. After incubation, cells were washed twice with PBS and subsequently incubated with fluorophore-conjugated secondary antibodies under the same conditions for 30 minutes. After two additional washes, cells were resuspended in PBS for analysis.

Samples were analyzed using an Apogee Flow Cytometry System (Apogee, UK) with consistent settings for laser intensity, fluorescence channels, and compensation values across all experimental groups. At least 10,000 cells were analyzed per sample, and data were processed using FlowJo software (BD, USA). FSC/SSC scatter plots were used to gate the cell population, excluding debris and doublets. Fluorescence intensity was quantified, and results were expressed as mean ± standard deviation (SD). Statistical significance was determined using appropriate methods (P < 0.05).

### 2.7 Enzyme-Linked Immunosorbent Assay (ELISA)

ELISA was conducted on culture supernatants or tissue homogenates to measure protein levels. Supernatants were centrifuged at 1000×g for 20 minutes and stored at -80°C. Tissue homogenates were prepared by grinding samples in pre-chilled PBS, followed by sonication or freeze-thaw lysis. Homogenates were centrifuged at 5000×g for 5-10 minutes, and the supernatant was collected.

Pre-coated ELISA plates were equilibrated to room temperature for 10 minutes. Each well received 100 µL of standard, sample, or blank control. Plates were incubated at 37°C for 1 hour. After discarding the liquid, 100 µL of biotin-labeled antibody working solution was added to each well, followed by a 1-hour incubation at 37°C and three washes. Then, 100 µL of enzyme conjugate working solution was added, and the plate was incubated for 30 minutes at 37°C, followed by five washes. Subsequently, 90 µL of TMB substrate was added and incubated at 37°C in the dark for 15 minutes before stopping the reaction with 50 µL of stop solution. Absorbance was measured at 450 nm using an Infinite 200 PRO microplate reader (Tecan, Switzerland). A standard curve was generated using a four-parameter logistic model. Results were expressed as mean ± SD, with statistical significance set at P < 0.05.

### 2.8 Cell Counting Kit-8 (CCK-8) Assay

The CCK-8 assay was used to evaluate cell viability. Cells were seeded into 96-well plates and allowed to grow to the logarithmic phase. Following treatment according to experimental protocols, 10 µL of CCK-8 solution was added to each well. Plates were gently mixed and incubated for 1-4 hours at 37°C, depending on the cell type. Absorbance at 450 nm was measured using a microplate reader. Blank wells containing only medium and CCK-8 solution were used to correct background absorbance. Results were expressed as the percentage of cell viability relative to control groups and reported as mean ± SD, with statistical significance set at P < 0.05.

### 2.9 Exosome Isolation and Characterization

Exosomes were isolated from culture supernatants using differential ultracentrifugation. Sequential centrifugation steps included 300×g for 10 minutes, 2000×g for 10 minutes, and 10,000×g for 30 minutes to remove debris, followed by ultracentrifugation at 100,000×g for 70 minutes to pellet exosomes. The pellet was resuspended in sterile PBS and washed again at 100,000×g for 70 minutes. All centrifugations were performed using a high-speed centrifuge and Sorvall WX+ ultracentrifuge (Thermo Scientific, USA).

Exosome morphology was examined by transmission electron microscopy (TEM; Hitachi, Japan). Samples were applied to carbon-coated copper grids, incubated for 10 minutes, and negatively stained with 2% phosphotungstic acid (pH 7.0) for 5 minutes. Exosome size and concentration were measured using nanoparticle tracking analysis (Zetasizer Nano ZS, Malvern Panalytical, UK). Zeta potential was measured at 25°C to assess surface charge properties. Exosome composition was analyzed using Fourier-transform infrared spectroscopy (FTIR; Nicolet iS50, Thermo Scientific, USA). Freeze-dried samples were mixed with KBr (1:100) and compressed into transparent pellets for spectral analysis over the range of 4000-400 cm⁻¹.

### 2.10 TUNEL Assay

TUNEL staining was used to detect apoptotic cells. Samples were fixed with 4% paraformaldehyde for 30 minutes, washed twice with PBS, and permeabilized with 0.3% Triton X-100 for 5 minutes. TUNEL reaction mixture was prepared following the manufacturer’s instructions, and 50 µL was applied to each sample. Incubation was conducted at 37°C in the dark for 1 hour. After washing with PBS, samples were counterstained with DAPI and mounted using anti-fade mounting medium. Red fluorescent signals were visualized using a confocal laser scanning microscope (excitation at 550 nm, emission at 570 nm). Positive cells were quantified using ImageJ software, with results expressed as mean ± SD. Statistical significance was determined at P < 0.05.

### 2.11 Reactive Oxygen Species (ROS) Detection

Reactive oxygen species (ROS) were detected using the DCFH-DA fluorescent probe. Cells were washed with PBS and incubated with 10 µM DCFH-DA in serum-free medium at 37°C for 30 minutes in the dark. For tissues, homogenates were prepared and incubated similarly. After incubation, cells were washed twice with PBS to remove excess probe. Fluorescent signals (excitation at 488 nm, emission at 525 nm) were observed under a fluorescence microscope (Leica, Germany). Quantification of fluorescence intensity or the percentage of positive cells was performed using ImageJ, and results were presented as mean ± SD. Statistical significance was set at P < 0.05.

### 2.12 Scratch Wound Assay

Cells were seeded in 6-well plates and cultured until a confluent monolayer was formed. A vertical scratch was made in the cell layer using a sterile 200 µL pipette tip. Detached cells were removed by washing with PBS, and serum-free medium was added to minimize cell proliferation effects. Plates were incubated at 37°C with 5% CO₂. Images of the scratch area were captured using a fluorescence microscope (Leica, Germany) at 0 hours and subsequent time points as defined in the experimental protocol.

The scratch closure area was measured using ImageJ software. The migration rate was calculated using the following formula:

$$\text{Migration Rate=}\frac{\text{Initial Area-Final Area}}{\text{Initial Area}}\times100\%$$

Results were expressed as mean ± standard deviation (SD). Statistical analysis was performed to assess significance, with P < 0.05 considered statistically significant.

### 2.13 SCI Model Construction

A SCI model was established using Allen’s weight-drop method. Female C57BL/6 mice (8 weeks old, 22 g) were anesthetized with 2% isoflurane and positioned on a surgical platform. Following a midline dorsal incision, the T10 vertebral laminae were removed to expose the spinal cord. A 10 g weight was dropped from a height of 2.5 cm onto the exposed spinal cord to induce contusion. Immediate spasmodic twitching of the hindlimbs and tail, along with hematoma formation and tissue indentation at the impact site, indicated successful mechanical injury. Locomotor function was evaluated using the BMS, and a decline to 0–3 points within 24 hours post-surgery confirmed the establishment of a severe SCI model.

After surgery, the wound was irrigated with sterile PBS, and the muscle and skin layers were sutured. All animals received standardized postoperative care, including subcutaneous injection of gentamicin (5 mg/kg/day for 3 days), daily manual bladder expression, use of a heating pad to maintain body temperature, and provision of softened high-calorie chow to promote nutritional intake. These supportive measures were applied consistently across all groups to ensure animal welfare and avoid confounding experimental outcomes.

### 2.14 Hematoxylin and Eosin (H&E) Staining

Tissue samples were fixed in 4% paraformaldehyde, paraffin-embedded, and sectioned at 5 µm thickness. Slides were dewaxed in xylene, rehydrated through a graded ethanol series to distilled water, and stained with hematoxylin for 5 minutes. Differentiation was performed with hydrochloric acid-ethanol, followed by bluing. Slides were counterstained with eosin for 2 minutes, dehydrated through graded ethanol, cleared in xylene, and mounted. Sections were examined under a light microscope, where nuclei appeared blue and cytoplasm pink.

## 3. Key Experimental Methods

### 3.1 Construction of GNA12 and GNA13 Overexpression Macrophages Using Lentiviral Infection

The GNA12 and GNA13 overexpression lentiviral vectors were obtained from GeneChem Co., Ltd. (Shanghai, China). RAW264.7 cells were seeded in 6-well plates at a density of 5×10³ cells per well and cultured at 37°C with 5% CO₂ for 12 hours. Lentiviral particles were added at a multiplicity of infection (MOI) determined by the experimental design. After 16 hours of infection, the medium was replaced with fresh medium containing 2 μg/mL puromycin for selection. Resistant cells were cultured until all control cells died, establishing stable macrophage overexpression cell lines. The macrophages were expanded, and overexpression of GNA12 and GNA13 was verified using qPCR and WB. Further, flow cytometry was used to assess macrophage phenotype, and functional assays were conducted to confirm the biological effects of GNA12 and GNA13 overexpression.

### 3.2 Induction of Neurotoxic Astrocytes

To induce the neurotoxic phenotype in astrocytes, microglial cells were first activated to the M1 phenotype. The activated microglial cells (5×10⁵ cells per well) were seeded in the upper chamber of a Transwell insert with a diameter of 6.5 mm and a pore size of 0.4 μm, while astrocytes (1×10⁶ cells per well) were plated in the lower wells of the culture plate. Both cell types were cultured in serum-free DMEM/F12 medium for 48 hours, allowing microglial cell secretions to act on astrocytes. At the end of co-culture, astrocytes were collected, and WB analysis using a C3-specific antibody was performed. The semi-quantitative analysis of C3 expression was used to confirm whether inflammatory microglial cells had successfully induced the neurotoxic phenotype in astrocytes. This experiment was conducted with reference to the study by Liddelow et al., published in Nature, which demonstrated that activated microglial cells induce astrocytes to adopt the A1 neurotoxic phenotype through the secretion of IL-1α, TNF, and C1q.

### 3.3 Exosome-Loaded Thermosensitive Hydrogel for Nasal Delivery

To prepare the thermosensitive chitosan-β-glycerophosphate (CS/β-GP) hydrogel for nasal delivery, 1.8% (w/v) chitosan (200 kDa molecular weight) was dissolved in pH 6.0 acetic acid solution under constant stirring at 4 °C. A 50% (w/v) β-glycerophosphate sodium solution was added dropwise at a 5:1 volume ratio under ice-bath conditions with gentle stirring to avoid premature gelation. To improve sprayability, 0.1% (v/v) polyethylene glycol 400 (PEG400) was added.exosome solution (final concentration: 100 μg/mL) was then introduced into the hydrogel precursor solution. The mixture was gently vortexed and subjected to low-power ultrasonic dispersion (water bath, ~30 kHz, 1 min) in an ice bath to ensure homogeneous distribution of exosomes while preserving vesicle integrity. This process enabled effective physical encapsulation of exosomes within the hydrogel matrix during subsequent thermally induced gelation. Gelation behavior was evaluated under simulated nasal conditions. For in vivo delivery, a micro-spray pump was used to administer the exosome-loaded hydrogel into the nasal cavity of SCI model mice. Delivery efficacy and therapeutic outcomes were compared with intravenous injection and exosome-only administration to verify the advantages of nasal delivery.

### 3.4 Transcriptomics and Pathway Analysis

Total RNA was extracted and subjected to RNA sequencing. Raw sequencing reads were initially subjected to quality control using FastQC to remove low-quality reads and adapter sequences. Clean reads were then aligned to the mouse reference genome (mm10) using HISAT2. Gene expression levels were quantified as fragments per kilobase of transcript per million mapped reads (FPKM) using StringTie. Differentially expressed genes (DEGs) were identified using DESeq2 after normalization, with thresholds set at |log₂ fold change| > 0.5 and adjusted *p-*value < 0.05.

Given that astrocytes are relatively stable glial cells characterized by gradual and functionally significant transcriptional modulations in response to external stimuli, a lower fold change threshold was adopted to comprehensively capture biologically relevant gene expression alterations induced by exosome treatment. This approach is consistent with previous studies investigating astrocyte transcriptomic responses under pathological conditions, thereby ensuring the reliability and comparability of the analysis^1, 2^.

Volcano plots were generated to illustrate the distribution of DEGs, and hierarchical clustering heatmaps were constructed to compare global expression patterns between experimental and control groups. Gene Ontology (GO) functional enrichment and Kyoto Encyclopedia of Genes and Genomes (KEGG) pathway analyses were conducted to explore the biological significance of DEGs. Additionally, gene set enrichment analysis (GSEA) was performed to identify significantly activated or repressed pathways between groups.

TGF-β Pathway Inhibition Validation

Astrocytes were pretreated with SB-431542 (TGF-β receptor 1 inhibitor, final concentration 10 μM) for 1 hour before exosome treatment. After 24 hours, cell lysates were collected, and WB was performed to measure the expression of TGF-β, Smad3, p*-*Smad3, PI3K p110β, p-PI3K p110β, NF-κB p65 (Rela), p-NF-κB p65, C3, and S100a10.

PI3K-AKT Pathway Validation

Astrocytes were seeded in 6-well plates (5×10⁵ cells per well) and cultured for 24 hours. Pik3cb siRNA or NC-siRNA (final concentration 50 nM) was transfected using RNAiMAX according to the manufacturer’s protocol. After 24 hours of transfection, exosome treatment was performed, and cell lysates were collected after another 24 hours. WB was conducted to assess the expression of PI3K p110β, p-PI3K p110β, AKT1, p-AKT1, C3, and S100a10.

RELA siRNA Validation

Astrocytes were transfected with Rela siRNA or NC-siRNA (final concentration 50 nM) as described above. After exosome treatment and 24-hour incubation, cell lysates were collected. WB was used to detect Rela (p65), p-Rela (p-p65), C3, and S100a10.

Smad3 siRNA Validation

Astrocytes were transfected with Smad3 siRNA or NC-siRNA (final concentration 50 nM) following the same protocol. After exosome treatment and 24-hour incubation, cell lysates were collected. WB was used to assess Smad3, p-Smad3, IκBα, Rela (p65), p-Rela (p-p65), C3, and S100a10 expression.

## 4. Experimental Procedures and Details

### 4.1 Identification of GNA12/GNA13 Overexpression in Macrophages

RAW264.7 cells were seeded in 6-well plates and infected with GNA12 or GNA13 lentivirus. Stable macrophage lines overexpressing GNA12 or GNA13 were selected following infection. The selected cells were subsequently characterized using the following methods: First, cell morphology, uniformity, and growth status were observed under an inverted optical microscope when the cells reached 70%-80% confluence. Second, the proliferation capacity of macrophages overexpressing GNA12 or GNA13 was assessed using the CCK-8 assay, and a proliferation curve was plotted. Finally, the mRNA expression levels of Gna12 and Gna13 were measured using qPCR, while GNA12 and GNA13 protein expression levels were analyzed via Western blot. Detailed protocols for these experiments can be found in the routine experimental methods section.

### 4.2 Effects of GNA12/GNA13 on Macrophage Polarization

To evaluate the effects of GNA12 and GNA13 overexpression on macrophage polarization, the selected cells were comprehensively analyzed using qPCR, Western blot, flow cytometry, and ELISA. qPCR was performed to measure the mRNA expression levels of M1 marker genes (Nos2, Cd68) and M2 marker genes (Tgf-β, Arg1, Cd163), with Gapdh serving as the internal control. WB analysis was used to detect protein levels of iNOS, CD68, TGF-β, ARG1, and CD163 with specific primary antibodies. FACS was employed to assess the polarization phenotype of macrophages by detecting M1 markers (iNOS, F4/80) and M2 markers (CD163, F4/80) using fluorescence-labeled antibodies. ELISA was utilized to quantify the concentrations of anti-inflammatory cytokines IL-10 and TGF-β in the cell culture supernatants, providing insights into the macrophages' anti-inflammatory properties. Detailed experimental procedures are described in the routine methods section.

### 4.3 Effects of GNA12/GNA13 on Macrophage Migration

To evaluate the impact of GNA12 and GNA13 overexpression on macrophage migration ability, Transwell assays, scratch assays, and cytoskeletal fluorescence staining were performed. In the Transwell assay, transfected macrophages were prepared as single-cell suspensions and seeded into the upper chamber of Transwell inserts with an 8 μm pore size. The lower chamber was filled with culture medium containing 10% fetal bovine serum (FBS) as a chemoattractant. After 24 hours of incubation, cells that migrated to the lower surface of the membrane were fixed with 4% paraformaldehyde and stained with DAPI. Migrated cells were then quantified under a fluorescence microscope. For the scratch assay, macrophages were seeded into 6-well plates, and vertical scratches were made using a sterile 200 μL pipette tip. After replacing the medium with serum-free culture medium, the cells were incubated at 37°C in a 5% CO₂ atmosphere, and images of the scratch area were captured at 0, 12, and 48 hours. For cytoskeletal fluorescence staining, macrophages were cultured on glass-bottom culture dishes until fully adherent. F-actin was stained with Phalloidin fluorescent dye, and cells were fixed with 4% paraformaldehyde. The cytoskeletal structures were observed and recorded using a confocal laser scanning microscope. These methods collectively assessed the effects of GNA12 and GNA13 overexpression on macrophage migration capacity.

### 4.4 Characterization and Identification of Exosomes

Exosomes were extracted from the culture supernatants of macrophages overexpressing GNA12 or GNA13. Detailed extraction procedures are described in the general experimental methods section.

### 4.5 Detection of Exosomal Marker Proteins and Overexpressed Proteins

Western blot was employed to analyze the expression of characteristic exosomal markers, including CD9, CD63, CD81, and HSP70, as well as the negative marker CALNEXIN, to verify the quality of exosome isolation. Additionally, the expression levels of GNA12, GNA13, and TGF-β in the exosomes were assessed to confirm the presence of specific proteins derived from overexpressing macrophages. Coomassie Brilliant Blue staining was performed on electrophoretically separated exosomal protein gels to visualize protein distribution and further validate exosomal characteristics. Detailed WB procedures are described in the general experimental methods section.

### 4.6 Analysis of Exosome Phagocytosis and Inflammatory Tropism

To evaluate the targeted phagocytosis and inflammatory tropism characteristics of macrophage-derived exosomes, DiI-labeled exosomes (final concentration 100 μg/mL) were co-cultured with microglial cells and astrocytes. After 12 hours, cells were washed with PBS, fixed, and stained with Iba1 and GFAP antibodies. Confocal microscopy was used to observe intracellular DiI signals, assessing the phagocytic targeting capacity of the exosomes. For inflammatory tropism analysis, microglial cells and astrocytes were seeded in the lower chambers of a 24-well plate with medium containing 50 ng/mL CCL2 to simulate an inflammatory environment. DiI-labeled exosomes were added to the upper Transwell chambers. After 12 hours, upper chamber cells were labeled with DiO and fixed. Fluorescence microscopy was employed to observe the distribution of DiI and DiO signals in lower chamber cells, evaluating the inflammatory tropism properties of the exosomes.

For flow cytometry-based quantification of exosome uptake, astrocytes were seeded in 6-well plates and treated with DiI-labeled exosomes from different groups under standard conditions. Where indicated, cells were pretreated with 10 μM Rhosin for 30 minutes before exosome administration to assess the role of RhoA signaling. After 12 hours of incubation, cells were washed thoroughly with PBS to remove unbound exosomes, trypsinized, and resuspended in FACS buffer. DiI fluorescence intensity was quantified using a BD flow cytometer, and mean fluorescence intensity (MFI) was calculated to assess relative exosome uptake efficiency.

### 4.7 Regulation of Microglial Cells by Exosomes

To evaluate the regulatory effects of macrophage-derived exosomes on microglial polarization and ROS levels, microglial cells were cultured to 70% confluence and treated with exosomes extracted from Gna12- and Gna13-overexpressing macrophages (final concentration 100 μg/mL) for 24 hours. Polarization was assessed by measuring the mRNA expression levels of Nos2 and Cd163 using qPCR and the protein expression levels of iNOS and CD163 using WB. IF was employed to analyze phenotypic polarization by evaluating the co-localization of INOS and CD163 with Iba1. Flow cytometry was used to quantify the proportions of INOS/Iba1 and CD163/Iba1 double-positive cells. ROS levels were measured using the DHE fluorescent probe, and fluorescence microscopy was utilized to observe and quantify the fluorescence intensity in exosome-treated microglial cells. Detailed experimental procedures are provided in the routine methods section.

### 4.8 Regulation of Astrocytes by Exosomes

To comprehensively evaluate the effects of exosomes on astrocyte polarization, inflammatory responses, ROS levels, and migration capacity, astrocytes were treated with exosomes at a final concentration of 100 μg/mL and subjected to multiple analyses. After 48 hours of treatment, WB was used to measure the protein expression levels of C3 and S100A10, and IF was employed to analyze the co-localization of C3 and S100A10 with GFAP to assess astrocyte polarization. WB was also performed to determine the expression levels of inflammatory-related proteins, including TNF-α, IL6, NLRP3, and IL10, to evaluate their dynamic changes. Following 24 hours of treatment, ROS levels were measured using the DHE fluorescent probe, and fluorescence microscopy was used to observe changes in ROS levels. Migration ability was assessed through scratch assays, with images of scratch areas captured at 0, 36, and 72 hours post-treatment. Detailed experimental procedures are provided in the routine methods section.

### 4.9 Validation of Enriched Pathways from Astrocyte Transcriptome Sequencing

Based on DEGs identified through transcriptome sequencing, key signaling pathways were explored using GO, KEGG, and GSEA. The TGF-β, PI3K-AKT, and NF-κB pathways were validated using the inhibitor SB-431542 and siRNAs targeting Pik3cb, Rela, and Smad3. Protein expression levels related to these pathways were assessed as described in the critical experimental methods section.

### 4.10 Crosstalk Between Astrocytes and Neurons

To investigate the crosstalk between astrocytes and neurons, a Transwell-based co-culture system was established. A1 astrocytes were generated as described above and treated with different exosome groups (100 µg/mL).

For apoptosis-related assays (Fig. 5a–i, Fig. S16), neurons were seeded into the lower chambers of 6.5 mm Transwell inserts (0.4 µm pore size) and cultured in Neurobasal medium supplemented with 2% B27 and 2 mM GlutaMAX under standard conditions (37 °C, 5% CO₂). Unstimulated astrocytes, A1 astrocytes, and exosome-treated A1 astrocytes were seeded into the upper chambers and co-cultured with neurons for 48 hours. Neuronal apoptosis was evaluated by Western blotting for Caspase-3, BAX, and BCL2, TUNEL staining, and Annexin V-FITC/PI flow cytometry.

After 24 hours of exosome treatment, BDNF and GDNF secretion by astrocytes was quantified using ELISA.

To assess glutamate clearance, astrocytes were exposed to 100 µM glutamate, and extracellular glutamate concentrations were measured using a glutamate assay kit. EAAT1 and EAAT2 protein expression levels were analyzed by Western blotting.

Phagocytic capacity was assessed by co-culturing astrocytes with DiI-labeled myelin debris (20 µg/mL) for 24 hours, followed by confocal microscopy and qPCR analysis of Megf10 and Mertk expression.

For synaptogenesis assays, astrocytes were co-cultured with neurons for 48 hours. Synaptic formation was assessed by qPCR analysis of Thbs1 and Gpc6 expression, Western blotting for Synaptophysin and SYN1, and confocal imaging of Synaptophysin and MAP2 colocalization.

Detailed experimental procedures are provided in the Methods section.

### 4.11 Crosstalk Between Astrocytes and NSCs

To investigate the regulatory effects of exosome-treated astrocytes (final concentration: 100 µg/mL) on the proliferation and differentiation of neural stem cells, a Transwell co-culture system was employed. A1 astrocytes were seeded into the upper chambers of 6.5 mm Transwell inserts (0.4 µm pore size), while NSCs were cultured in the lower chambers. NSCs were maintained under two conditions: proliferation medium (KnockOut DMEM/F-12 supplemented with 20 ng/mL bFGF, 20 ng/mL EGF, 2% B27, 1% N2, and 2 mM GlutaMAX) for 48 hours, and differentiation medium (DMEM/F-12 supplemented with 10 ng/mL bFGF, 10 ng/mL EGF, and 2% B27) for 7 days. The Transwell inserts allowed secreted factors from astrocytes to interact with NSCs without direct cell-cell contact.

For proliferation analysis, astrocytes and NSCs were co-cultured for 48 hours, followed by the addition of EdU working solution (final concentration: 10 µM) to the NSC medium. After an additional 24 hours, cells were fixed, and the proportion of EdU-positive cells was quantified by confocal microscopy. In the CCK-8 assay, NSC spheres were transferred to 96-well plates after 48 hours of co-culture, and CCK-8 reagent was added to measure absorbance, providing an evaluation of proliferation levels.

For differentiation analysis, astrocytes and NSCs were co-cultured for 7 days. NSCs were then fixed and immunolabeled with anti-MAP2 and anti-GFAP antibodies to identify neurons and glial cells, respectively. Differentiation ratios were assessed by confocal microscopy. Additionally, total protein was extracted from NSCs for Western blotting to detect NF200, VSX2, PI3K p110, and AKT1, key proteins involved in neural differentiation pathways.

To further validate the therapeutic effects of exosomes in vivo, spinal cord tissues were harvested from mice 28 days post-injury. VSX2 immunofluorescence staining was performed on tissue sections to evaluate NSC differentiation.

Detailed protocols for the CCK-8 assay, immunofluorescence staining, and Western blotting are provided in the routine methods section.

### 4.12 Hydrogel Characterization, Sustained Release Performance and Hydrogel Degradation

To characterize the properties and sustained release performance of exosome-loaded hydrogels, multiple analytical methods were employed. For freeze-drying treatment, the hydrogel samples were frozen at -80°C for 4 hours, followed by vacuum freeze-drying under low pressure at -50°C to -60°C for 48 hours to preserve their porous structure. Rheological analyses, including gelation temperature, frequency response, and stress response tests, were conducted to evaluate the gelation properties and dynamic mechanical behavior of the hydrogels.

Scanning electron microscopy (SEM) combined with energy-dispersive X-ray spectroscopy (EDS) was used to observe the microstructural pores of the freeze-dried hydrogels and analyze the distribution of elements such as C, N, O, and P. Alexa Fluor 594-conjugated chitosan was used to label the hydrogel matrix, and DiO fluorescent dye was applied to label exosomes. The labeled exosomes were then loaded into the hydrogel. After gelation, the samples were sectioned, and confocal microscopy was used to acquire fluorescence images, followed by three-dimensional reconstruction to visualize the spatial distribution of exosomes within the fluorescently labeled hydrogel network.

Fourier-transform infrared spectroscopy (FTIR) was employed to analyze the chemical composition and functional groups of the freeze-dried hydrogels. Spray performance was evaluated by capturing high-resolution images of the spray morphology to assess symmetry and uniformity.

For sustained release testing, exosome-loaded hydrogel samples were placed in dialysis bags, fully immersed in 1 mL of PBS (pH 6.0) to simulate the nasal mucosal environment, and incubated at 34 °C in a shaking incubator at 50 rpm^3, 4, 5^ . Dialysate samples were collected at predetermined intervals, and the exosome concentration was quantified using the BCA assay. Cumulative release curves were plotted to assess the sustained release behavior of the hydrogels.

To evaluate the biodegradability of the hydrogel, both in vitro and in vivo degradation studies were conducted. For in vitro assessment, pre-formed CS/β-GP hydrogels (~100 μL) were incubated in PBS containing 1 mg/mL lysozyme (pH 6.0) at 34 °C. Degradation was monitored at multiple time points (4 h, 8 h, 12 h, 16 h, 20 h, and 24 h) by freeze-drying the samples and calculating mass loss. For in vivo testing, fluorescently labeled hydrogel was administered intranasally to mice, and degradation was evaluated at 24 h intervals (Day 1 to Day 4) by two methods: (1) dissection and weighing of residual hydrogel from the nasal cavity after freeze-drying, and (2) nasal lavage followed by fluorescence quantification. A time-lapse of the lavage procedure was recorded and provided as Supplementary Video S1.

### 4.13 Biocompatibility and Targeting of the Hydrogel

To assess the cytocompatibility of the hydrogel, A549 cells were seeded into 96-well plates at a density of 1 × 10⁴ cells per well and cultured overnight. Preformed CS/β-GP hydrogel was added to each well at final concentrations of 0, 1, 2.5, 5, and 10 mg/mL, followed by incubation for 24 hours. For viability assessment, a Live/Dead Cell Viability Assay Kit was used. After 24 hours of hydrogel exposure, cells were washed with PBS and stained with Calcein-AM and EthD-1 according to the manufacturer’s instructions. Fluorescence images were acquired using a confocal microscope (excitation/emission: 488 nm/530 nm for live cells and 530 nm/620 nm for dead cells).

To evaluate the biocompatibility of the CS/β-GP hydrogel loaded with exosomes, H&E staining of the nasal mucosa and major organs, along with blood biochemical analysis, was performed. For nasal biocompatibility analysis, 50 μL of hydrogel was evenly sprayed into the nasal cavity of mice using a micro-spray pump daily for seven consecutive days. The mice were euthanized, and the nasal mucosa tissues were collected for H&E staining to observe mucosal morphology, inflammatory cell infiltration, and tissue integrity under a microscope. For the major organ biocompatibility analysis, organs were collected on day 28 after administration, and H&E staining was performed to examine cell morphology, structural integrity, and inflammatory infiltration. Details of the H&E staining procedure are provided in the routine methods section. Additionally, blood biochemical analysis was conducted on day 14 after administration. Blood samples were collected via cardiac puncture, and serum was separated by centrifugation to assess major physiological indicators and evaluate systemic toxicity and safety of the hydrogel.

To evaluate whether intranasally delivered exosomes enter CSF, DiD-labeled exosomes were administered to SCI mouse via nasal instillation using a microspray device. At 24 hours post-administration, mice were euthanized, and CSF samples were collected using two standard approaches: cisterna magna puncture and occipital foramen access. All procedures were performed under a surgical microscope with strict aseptic technique to minimize blood contamination.Collected CSF samples were transferred to black 96-well plates, and fluorescence intensity was directly measured using a microplate reader (excitation/emission: 644/665 nm for DiD). PBS-treated animals served as negative controls. Fluorescence signal intensities were recorded and compared between groups to determine exosome presence in the CSF. All procedures were conducted with care to preserve sample integrity due to the limited CSF volume (~30 μL per mouse). Measurements were performed in triplicate at each time point.

To investigate the in vivo targeting capability of the exosome-loaded hydrogel, small-animal imaging and frozen tissue sectioning were used to detect the distribution of exosomes. First, the targeting differences between intranasal administration and intravenous injection of exosomes were compared. DiD-labeled exosomes (100 μg per dose) were administered once daily for three consecutive days via intranasal or intravenous routes to mice with SCI. After 7 days of treatment, the mice were euthanized, and tissues from the SCI region and major organs were collected. Fluorescence images were captured using a small-animal imaging system, and fluorescence intensity was quantified to assess exosome accumulation in the SCI region and distribution in other organs.

To further evaluate the time-dependent tissue distribution and retention of exosomes, DiD-labeled exosome-loaded hydrogel was administered intranasally, and both in vivo and ex vivo fluorescence imaging were performed at 3 hours, 24 hours, 3 days, 5 days, and 7 days post-administration. Ex vivo imaging of freshly isolated spinal cords was conducted to eliminate potential imaging artifacts caused by fur or surgical incisions. Fluorescence intensities in the nasal and spinal regions were quantified using Living Image software to assess delivery specificity and retention dynamics.

For tissue-level localization analysis, frozen tissue sections were prepared. On day 7 post-administration, SCI spinal cord tissues were harvested, fixed in 4% paraformaldehyde, cryoprotected with 30% sucrose, embedded in OCT, and sectioned using a cryostat. To identify cell types that internalized exosomes, immunofluorescence staining was performed using primary antibodies against Iba1 (microglia), GFAP (astrocytes), and NeuN (neurons), followed by fluorescent secondary antibodies. Co-localization of DiD-labeled exosomes with different cell types was observed using a laser scanning confocal microscope. Z-stack imaging and colocalization analysis were performed using ImageJ software to confirm exosome uptake patterns. These data revealed prominent exosome internalization in microglia and astrocytes, with minimal uptake detected in NeuN⁺ neurons.

### 4.14 Functional Evaluation SCI

To comprehensively evaluate the therapeutic effects of exosome-loaded hydrogel in a mouse model of SCI, parameters including motor function, body weight changes, and bladder function were analyzed. The BMS scoring system was used to assess hindlimb motor function, with scores recorded on days 1 through 35 post-SCI and treatment. The double-blinded scoring results were presented as mean ± standard error of the mean (SEM), and recovery trends were visualized with line graphs.

Body weight was measured before injury and at designated time points post-injury to reflect overall health status. On day 28, an open-field footprint test was performed to analyze hindlimb gait characteristics. Additionally, swimming scores were recorded on days 7, 14, and 28 to evaluate hindlimb motor ability, coordination, and propulsion.

Bladder function was assessed by H&E staining of bladder tissue collected on day 28 post-treatment, examining structural integrity and thickness changes to provide a comprehensive evaluation of the hydrogel's therapeutic potential.

### 4.15 Histological and Apoptotic Analysis SCI

To investigate the effects of exosome-loaded hydrogel on histological repair and apoptosis regulation in SCI, analyses including H&E staining, TUNEL staining, Nissl staining, and IF were performed. For histological observation, spinal cord tissue from the SCI region was collected on day 28 post-treatment and analyzed for structural changes via H&E staining (detailed protocol in routine methods).

For apoptosis evaluation, TUNEL staining combined with DAPI counterstaining was performed on SCI tissue collected on day 2 post-injury to observe and quantify the proportion of apoptotic cells. Neuronal survival was assessed on day 7 post-treatment using Nissl staining to record the morphology and number of Nissl-positive neurons.

IF analysis was conducted on day 28 post-treatment using antibodies against GFAP and Tuj1 to examine the morphology and distribution of neurons. These combined methodologies provided a comprehensive assessment of neuronal density and apoptosis in SCI, elucidating the mechanistic role of exosome-loaded hydrogel in SCI repair. Detailed procedures are outlined in the routine methods section.

### 4.16 Transcriptomic Analysis SCI

On day 28 post-injury, SCI tissue was collected, flash-frozen in liquid nitrogen, and stored at -80°C. Total RNA was extracted using TRIzol, and RNA quality was ensured before proceeding with transcriptomic sequencing by a certified sequencing company.

The sequencing results were analyzed to compare differential gene expression between experimental groups. Key genes related to SCI repair and inflammation regulation were identified, and their expression patterns were visually presented using heatmaps, with gene names and sample groups clearly labeled.

## 5. Statistical Analysis

All experiments were performed in triplicate or more. Data are presented as mean ± standard deviation (mean ± SD). Statistical analyses were conducted using GraphPad Prism 9.0. One-way analysis of variance (ANOVA) followed by Tukey’s multiple comparisons test was used for comparisons among multiple groups. For experiments involving time-course or dose-response variables across different treatments, two-way ANOVA followed by Tukey’s post hoc test was applied to assess main effects and interactions.Statistical significance was considered at P < 0.05. Significance levels are indicated in the figures as follows: P < 0.05 (*, #), P < 0.01 (**, ##), and P < 0.001 (***, ###). Further statistical details are specified in the corresponding figure legends.

1. Dejanovic B, Wu T, Tsai MC, Graykowski D, Gandham VD, Rose CM*, et al.* Complement C1q-dependent excitatory and inhibitory synapse elimination by astrocytes and microglia in Alzheimer's disease mouse models. *Nat Aging* 2022, **2**(9)**:** 837-850.

2. Slota JA, Sajesh BV, Frost KF, Medina SJ, Booth SA. Dysregulation of neuroprotective astrocytes, a spectrum of microglial activation states, and altered hippocampal neurogenesis are revealed by single-cell RNA sequencing in prion disease. *Acta Neuropathol Commun* 2022, **10**(1)**:** 161.

3. Maniyamgama N, Bae KH, Chang ZW, Lee J, Ang MJY, Tan YJ*, et al.* Muco-Penetrating Lipid Nanoparticles Having a Liquid Core for Enhanced Intranasal mRNA Delivery. *Adv Sci (Weinh)* 2025, **12**(11)**:** e2407383.

4. Liu Y, Tan Y, Cheng G, Ni Y, Xie A, Zhu X*, et al.* Customized Intranasal Hydrogel Delivering Methylene Blue Ameliorates Cognitive Dysfunction against Alzheimer's Disease. *Adv Mater* 2024, **36**(19)**:** e2307081.

5. Hong Y, Song H, Gong Y, Mao Z, Gao C, Shen J. Covalently crosslinked chitosan hydrogel: properties of in vitro degradation and chondrocyte encapsulation. *Acta Biomater* 2007, **3**(1)**:** 23-31.
